# An Improved Whale Migration Optimization Algorithm for Cooperative UAV 3D Path Planning

**DOI:** 10.3390/biomimetics10100655

**Published:** 2025-10-01

**Authors:** Zhanwei Liu, Shichao Li, Hong Xu

**Affiliations:** 1 School of Information Science and Technology, Shijiazhuang Tiedao University, Shijiazhuang 050043, China; whoso4408@gmail.com; 2 Hebei Key Laboratory of Electromagnetic Environmental Effects and Information Processing, Shijiazhuang 050043, China; 3 School of Information Science and Technology, Sifang College, Shijiazhuang Tiedao University, Shijiazhuang 050228, China; xuhong@stdu.edu.cn

**Keywords:** Improved Whale Migration Algorithm, cooperative search, CEC2017, path planning, Multi-UAVs

## Abstract

This study proposes an Improved Whale Migration Algorithm (IWMA) to overcome the shortcomings of the original Whale Migration Algorithm, which suffers from premature convergence and insufficient local exploitation in high-dimensional multimodal optimization. IWMA introduces three enhancements: circle chaotic initialization to improve population diversity, a three-layer cooperative search framework to achieve a stronger balance between exploration and exploitation, and a dynamic adaptive mechanism with t-distribution re-exploration to reinforce both global escaping and local refinement. On the CEC2017 benchmark suite, IWMA demonstrates clear superiority over seven representative algorithms, delivering the best results on 27 out of 29 functions by best, 25 by mean, and 23 by standard deviation in 30 dimensions, and on 25, 18, and 18 functions, respectively, in 50 dimensions. Compared with other migration-based optimizers, its average rank improves by more than 30 percent, while runtime analysis shows only a small additional overhead of 7 to 12 percent. These outcomes, supported by convergence curves, boxplots, radar charts, and Wilcoxon tests, confirm the effectiveness of the proposed improvements. In six multi-UAV path planning scenarios, IWMA reduces the average cost by 14.5 percent compared with WMA and achieves up to 32.1 percent reduction in the most complex case. Overall, its average cost decreases by 27.4 percent across seven competitors, with a 23.6 percent improvement in the best solutions. These results demonstrate that the proposed modifications are effective, enabling IWMA to transfer its performance gains from benchmark tests to practical multi-UAV cooperative mission planning, where it consistently produces safer and smoother trajectories under complex constraints.

## 1. Introduction

In recent years, unmanned aerial vehicles (UAVs) have gained wide adoption in monitoring, communication, mapping, and inspection owing to their small footprint, strong maneuverability, and flexible deployment [1,2,3,4]. During autonomous flight, three-dimensional (3D) path planning is central to ensuring safety, mission efficiency, and energy economy; its goal is to generate an optimal or near-optimal trajectory that satisfies mission constraints in complex airspace [5,6]. An ideal path must avoid obstacles while accounting for flight dynamics, energy limitations, and time constraints, thereby balancing global optimality with local feasibility [7,8,9]. As UAV mission environments evolve toward higher dimensionality, multiple constraints, and dynamic uncertainty, the complexity of path planning increases markedly [10,11,12]. Against this backdrop, a core scientific challenge in autonomous flight is to maintain a strong global search capability without sacrificing convergence accuracy and computational efficiency [13,14,15].

UAV path-planning algorithms can be broadly divided into conventional methods and metaheuristics. Among classical global planners, the A* algorithm guarantees optimality in static environments but incurs rapidly escalating computational and memory costs as the search space grows, limiting performance in large-scale 3D planning [16,17,18,19,20]. In continuous spaces, rapidly-exploring random trees (RRT) and their variant RRT* efficiently produce feasible trajectories, yet they struggle to reconcile path smoothness, global optimality, and computational complexity [21,22]. The artificial potential field (APF) method is simple and efficient but susceptible to local minima, offering no guarantee of global optimality [23].

With advances in intelligent optimization, metaheuristic algorithms have become indispensable tools for UAV path planning [24], aiming to solve complex optimization problems by mimicking natural or social phenomena [25]. Based on their sources of inspiration, metaheuristic algorithms are generally categorized into four groups: (1) evolutionary algorithms (EAs), such as Genetic Algorithms (GAs) [26] and Differential Evolution (DEs) [27],which rely on natural selection and genetic operators for evolutionary search; (2) Swarm Intelligence Algorithms (SIAs), including Particle Swarm Optimization (PSO) [28], Grey Wolf Optimizer (GWO) [29], and Whale Optimization Algorithm (WOA) [30], which simulate collective cooperation and competitive behaviors for global search; (3) Physics-Inspired Algorithms (PIAs), such as Simulated Annealing (SA) [31] and the Gravitational Search Algorithm (GSA) [32], which leverage physical laws or dynamic principles for optimization; (4) Human/Social-Inspired Algorithms (HSAs), such as Teaching-Learning-Based Optimization (TLBO) [33], which draws on human learning and social interactions to enhance search efficiency.

Despite their widespread use, these algorithms often exhibit insufficient population diversity, limited local exploitation, and a tendency to become trapped in local optima in high-dimensional or complex tasks [34,35]. To address these limitations, numerous enhancements have been proposed. For example, Shao [36] introduced adaptive linear acceleration coefficients and maximum-velocity regulation in PSO. LiG [37] developed an adaptive-learning PSO that automatically selects among four update strategies to improve trajectory quality. Xue H. [38] incorporated sine-cosine operators to further enhance particle update performance. Hybrid strategies have also been widely investigated: Korashy A. et al. [39] integrated the GWO leadership structure into WOA’s exploitation phase; Luo et al. [40] combined DE with WOA to improve both local and global search; Kaur et al. [41] employed chaotic maps to adjust WOA parameters and balance exploration and exploitation; Prasad et al. [42] used a logistic map to mitigate premature convergence. While these improvements enhance performance, they generally suffer from structural complexity, limited scalability, or high parameter sensitivity.

Despite these improvements, conventional metaheuristics continue to face fundamental challenges in maintaining population diversity and avoiding premature convergence in complex optimization landscapes. This has led researchers to explore novel bio-inspired paradigms, among which migration-based algorithms have emerged as a particularly promising approach. In this context, migration-based algorithms, a novel subclass of swarm intelligence methods, have gained increasing attention. Inspired by large-scale migratory behaviors in nature, these algorithms emphasize collective coordination, dynamic adaptation, and long-range exploration, effectively addressing limitations of conventional approaches through their inherent ability to maintain population diversity across vast search spaces. Representative migration-based methods include the Artificial Lemming Algorithm (ALA) [43], Grey Goose Optimizer (GGO) [44], Seagull Optimization Algorithm (SOA) [45], and Walrus Optimization Algorithm (WaOA) [46], which demonstrate the potential of migration-inspired strategies while highlighting challenges in efficiency, robustness, and adaptability.

To address these challenges, the Whale Migration Algorithm (WMA) [47] was recently proposed, inspired by the collective migratory behavior of humpback whales. Among migration-based algorithms, WMA was selected as the foundational framework for several compelling reasons: (1) algorithmic novelty—as a recently proposed migration-inspired method WMA represents the current state-of-the-art in this paradigm while offering substantial potential for further enhancement; (2) structural efficiency—WMA employs a streamlined leader-follower mechanism with minimal parameter requirements, providing greater extensibility and robustness compared to more complex alternatives such as GGO [44] or WaOA [46]; (3) algorithmic advantages—WMA’s collective migration strategies and adaptive mechanisms possess theoretical advantages in handling high-dimensional search spaces, with its balanced exploration-exploitation design philosophy aligning well with the requirements of complex optimization problems; (4) implementation practicality—WMA’s lightweight design facilitates integration with enhancement strategies without introducing excessive computational overhead.

Nevertheless, in-depth analysis reveals that the original WMA still exhibits several critical deficiencies: initialization limitations—the random initialization strategy struggles to ensure uniform population distribution and adequate coverage in high-dimensional spaces; population diversity degradation—the lack of effective diversity maintenance mechanisms during iterations leads to premature population convergence; insufficient local exploitation—the leader-follower mechanism demonstrates limited fine-grained search capability in multimodal environments, failing to fully exploit local optima potential; exploration-exploitation imbalance—fixed search strategies cannot dynamically adjust the balance between exploration and exploitation according to search progress; and parameter sensitivity—critical parameter settings significantly impact algorithmic performance without adaptive adjustment mechanisms. These limitations constrain WMA’s performance in complex optimization problems and provide clear directions for further improvement.

Based on these considerations, this study proposes an Improved Whale Migration Algorithm (IWMA) with three key innovations. First, chaotic initialization using Circle mapping enhances population diversity and distribution coverage. Second, a three-layer cooperative search framework divides the population into elite, intermediate, and follower layers, where the elite layer applies Gaussian perturbations for intensified local exploitation, the intermediate layer performs mean-guided hybrid search, and the follower layer conducts large-step differential-mutation exploration, achieving a dynamic balance between exploration and exploitation. Third, an adaptive mechanism with elite re-exploration regulates search intensity via a decreasing exploration coefficient, periodically tuned differential weights, and t-distribution perturbations to improve both global escape and fine-grained refinement.

To validate its effectiveness, IWMA was benchmarked against seven representative algorithms, including PSO [28], GWO [29], Dung Beetle Optimizer (DBO) [48], Giant Trevally Optimizer (GTO) [49], Ivy Optimizer (IVY) [50], the original WMA, the advanced evolutionary baseline Success-History based Adaptive Differential Evolution with Linear Population Size Reduction (L-SHADE) [51], hereafter referred to as LSHADE in experimental figures and tables for consistency with implementation. For empirical validation, we first evaluate IWMA on the CEC2017 benchmark suite at 30 and 50 dimensions, where it significantly outperforms six classical metaheuristics in convergence accuracy, robustness, and adaptability; Wilcoxon signed-rank tests further confirm statistical superiority. We then apply IWMA to multi-UAV cooperative 3D path planning across six representative scenarios. The results show that IWMA yields smoother, safer trajectories under complex constraints, demonstrating strong practicality and application potential. The main contributions of this paper are summarized as follows:

1. An improved algorithm design. We propose the Improved Whale Migration Algorithm (IWMA), a WMA variant that integrates chaotic initialization, a three-layer cooperative search framework, and adaptive mechanisms, thereby addressing the deficiencies of the original WMA in terms of convergence accuracy and local exploitation.
2. Comprehensive benchmark evaluation. We conduct systematic experiments on the CEC2017 benchmark set (30D and 50D), and employ multi-metric visualizations (e.g., radar charts and histogram-line plots) to comprehensively demonstrate the superiority of IWMA in convergence accuracy, robustness, and adaptability.
3. Application to UAV path planning. We apply IWMA to multi-UAV three-dimensional path planning and validate its effectiveness and robustness in six representative constrained scenarios, showing its strong potential for practical deployment.

The rest of this paper is organized as follows. Section 2 introduces the original WMA. Section 3 presents the proposed IWMA and analyzes its computational complexity. Section 4 reports benchmark experiments on the CEC2017 test suite, including comparisons, visual analyses, runtime evaluation, ablation studies, and further tests against other migration-based optimizers. Section 5 applies IWMA to multi-UAV 3D path planning in six complex scenarios. Section 6 concludes the paper and discusses future research directions.

## 2. The Original Whale Migration

The Whale Migration Algorithm (WMA) is a newly emerging bio-inspired intelligent optimization algorithm, whose design is motivated by the cooperative migratory behavior of humpback whales. In nature, whales exhibit complex and efficient cooperative strategies during seasonal migration; some whales act as leaders, responsible for identifying suitable habitats and feeding areas, while the rest follow and learn to achieve stable group migration and optimal energy allocation. This “leader-follower” mechanism reflects a dynamic balance between global exploration and local exploitation, providing new insights for addressing complex optimization problems.

### 2.1. Initialization

WMA first generates an initial population of size $N_{pop}$ within the search space [*L*,*U*], where the position vector of each individual is defined as shown in Equation (1):

(1) $$W_{i}=L+rand\left(1,D\right)⨀\left(U-L\right),i=1,2,\ldots ,N_{pop}$$

Among them, *D* represents the problem dimension, *L* and *U* denote the lower and upper bounds of the search space, respectively, and ⨀ indicates the Hadamard product. This process ensures randomness and diversity in the search, laying the foundation for subsequent exploration and exploitation. Then, the algorithm evaluates the fitness of each individual ${f(W}_{i})$ and selects the best solution as the global best $g_{Best}$, where $N_{L}\approx N_{pop}/2$.

### 2.2. Exploration Phase

During the iteration process, WMA sorts the population according to fitness in descending order and selects the top $N_{L}\approx N_{pop}/2$ individuals as the leader whale group. The leader individuals play an exploratory role during migration, and their average position is used to guide the direction of the entire whale population:

(2) $$W_{Mean}=\frac{1}{N_{L}}\sum_{j=1}^{N_{L}}W_{j}$$

In Equation (2), $W_{j}$ denotes the position of the *j*, the leader individual, while $W_{Mean}$ represents the “average direction” of the entire leader group. It provides a global reference point for subsequent individual updates, reflecting the role of collective intelligence in guiding the migration process.

#### 2.2.1. Leadership Behavior

The leader individuals are responsible for global exploration, and their position update formula is given as follows:

(3) $$W_{i}^{t+1}=W_{i}+r_{1}⨀L+r_{2}⨀r_{1}⨀(U-L),i=1,\ldots ,N_{L}$$

In Equation (3), $r_{1}$ and $r_{2}$ are random vectors, while L and U denote the lower and upper bounds, respectively. The first term of the formula represents a perturbation based on the original position, whereas the second term guides individuals to resample within the entire search space. As a result, the leader individuals can continuously explore new regions, effectively “paving the way” on a global scale and helping the algorithm escape from local optima.

#### 2.2.2. Following Behavior

The position update of ordinary individuals follows the rule:

(4) $$W_{i}^{t+1}=W_{mean}+r_{1}⨀\left(W_{i-1}-W_{i}\right)+r_{2}⨀\left(W_{Best}-W_{Mean}\right),i=N_{L+1},\ldots ,N_{pop}$$

In Equation (4), $W_{Best}$ denotes the current global best solution, and $r_{1}$ and $r_{2}\in [0,1]$ are random vectors. This formula indicates that ordinary individuals are guided not only by the mean of the leader group but also by the positional difference of the previous individual, as well as the gap between the global best and the leader mean. On the one hand, this enhances the population’s local learning ability, while on the other hand, it maintains a connection to the global best, thereby preventing the group from becoming overly concentrated.

### 2.3. Exploitation Phase

After completing the position update in the exploration phase, WMA enters the exploitation phase. At this stage, the algorithm evaluates the fitness of all individuals. If the new position outperforms the original one, the update is retained; otherwise, the individual reverts to its previous state. This mechanism ensures that the search process always proceeds toward better solutions, avoiding performance degradation caused by ineffective updates. Meanwhile, the algorithm continuously maintains the global best solution in each generation. Whenever an individual’s fitness surpasses the current global best, it is immediately updated and recorded. This guarantees that the historically best solution is preserved, allowing the algorithm to gradually converge toward the optimal region.

From a biological perspective, the exploitation phase models the whales’ tendency to “settle in superior habitats” during migration, that is, to remain at and utilize a favorable site once it is identified. This mechanism helps the population maintain a balance between exploration and exploitation, with a stronger emphasis on fine-grained local search in the later stages, thereby improving the stability of global convergence.

### 2.4. Boundary Handling and Termination Conditions

During the iterative updates, individuals may exceed the feasible search space due to excessive step size or directional deviations. To ensure the feasibility of solutions and the effectiveness of the optimization process, WMA introduces a boundary correction mechanism, as defined in Equation (5):

(5) $$W_{i,d}=\left\{\begin{array}{c} U_{d},W_{i,d}>U_{d}\\ L_{d},W_{i,d}<L_{d}\\ W_{i,d},otherwise \end{array}\right.$$

The strategy projects infeasible solutions that exceed the search boundaries directly onto the corresponding boundary positions through truncation, ensuring that individuals always remain within the feasible search space and avoiding invalid solutions. In practical applications, besides truncation, alternative approaches such as reflection mapping (reflecting the infeasible position back into the domain) or random re-initialization (resampling within the feasible range) can also be applied to balance feasibility and population diversity.

Regarding termination conditions, WMA typically halts once the maximum number of iterations $T_{max}$ is reached. In addition, an early stopping criterion may be introduced when the global best solution fails to improve over a number of consecutive generations, thereby avoiding unnecessary computational costs. For specific engineering optimization tasks, problem-dependent thresholds or computational resource limits can also be incorporated as supplementary termination criteria.

In summary, boundary handling ensures solution feasibility and integrity of the search space, while termination conditions control the convergence and computational efficiency of the optimization process. Together, these mechanisms enable WMA to maintain robustness while ensuring adaptability and practical applicability in real-world optimization tasks.

### 2.5. Summary and Characteristics

In summary, the workflow of WMA can be outlined as follows: population initialization, division into leader and follower individuals, follower imitation learning (exploitation), leader-driven global exploration (exploration), fitness evaluation and global best updating, boundary correction, and convergence checking. This “leader exploration + follower exploitation” dual mechanism effectively integrates global search and local refinement capabilities. Compared with traditional optimization algorithms, WMA maintains population diversity while exhibiting strong convergence performance, making it well-suited for solving high-dimensional optimization problems with complex constraints.

## 3. Improved Whale Migration Algorithm

Although the Whale Migration Algorithm (WMA) establishes a two-layer mechanism of “leader exploration + follower imitation” to balance global search and local exploitation, it still suffers from several limitations. On one hand, the random distribution of the initial population may not sufficiently cover the search space, resulting in low early-stage search efficiency. On the other hand, in WMA, individuals are simply divided into leaders and ordinary members, leading to a lack of fine-grained division of labor. Consequently, it is difficult to simultaneously achieve high-precision local exploitation and large-step global exploration. Moreover, the search behaviors in the original algorithm are relatively fixed and lack dynamic regulation, which may cause premature convergence to local optima in later iterations. To address these drawbacks, this paper proposes an Improved Whale Migration Algorithm (IWMA), which introduces three innovative strategies.

### 3.1. Circle Chaotic Initialization Strategy

Previous studies [52] have indicated that when the number of iterations is sufficiently large, most evolutionary algorithms become largely insensitive to different initialization strategies, including those involving chaotic maps such as the Circle chaotic mapping. However, the Whale Migration Algorithm (WMA), guided by a “leader-follower” cooperative mechanism, differs markedly from conventional evolutionary algorithms in its individual behavior and population coordination. In WMA, the initial population distribution directly determines potential leaders and their guiding influence, thereby shaping the early-stage exploration landscape. To leverage this property, the Improved Whale Migration Algorithm (IWMA) incorporates Circle chaotic mapping into its initialization, aiming to enhance population coverage and uniformity under limited sample sizes. This approach strengthens early global exploration and supplies more representative candidate leaders for the subsequent three-layer cooperative search.

In IWMA, a chaotic mapping is first introduced to enhance population diversity during initialization. The initialization formula is defined as Equation (6):

(6) $$W_{i}=L+ChaosMap(N_{pop},D)⨀\left(U-L\right)$$

where the chaos map adopts the Circle chaotic mapping [53], whose iterative formula is given by Equation (7):

(7) $$x_{i}\left(t+1\right)=mod\left(x_{i}\left(t\right)+a-\frac{b}{2\pi x_{i}\left(t\right)},1\right)$$

Compared with traditional uniform random initialization, the Circle chaotic sequence possesses both ergodicity and determinism, which enables a more uniform coverage of the search space. This avoids clustering of initial solutions in local regions and improves early exploration ability. Thus, IWMA can obtain more potential high-quality solutions in the early stage, accelerating the expansion of global search.

### 3.2. Three-Layer Cooperative Search Framework

In the original WMA, the population is divided into a leader group and a follower group, with leaders guiding the search based on their mean position. This two-layer structure is limited in complex problems: leaders must handle both global exploration and local exploitation, while followers search passively, often causing insufficient local refinement or restricted global exploration in later iterations.

Several metaheuristics have introduced hierarchical or cooperative population structures to balance exploration and exploitation. For instance, HADE-LS partitions the population into four fitness-based layers with layer-specific local search and adaptive DE operators [54]; HMS-GWO employs a multi-step hierarchical decision framework among wolf ranks to enhance diversity and avoid stagnation [55]; MUDE divides the population into multiple subpopulations with different mutation strategies and soft-island migration combined with uniform local search [56]; HRIME-MSP applies a superior–borderline–inferior layered structure within the RIME framework, assigning exploitative, expert, and explorative operators to each layer [57]. Despite these advances, challenges remain in inter-layer coordination, search diversity, and elite re-exploration.

To address these issues, IWMA introduces an elite–intermediate–follower three-layer framework. The elite layer performs Gaussian perturbations for high-precision local search; the intermediate layer bridges exploration and exploitation via mean attraction and random mixed search; the follower layer executes differential-based large-step perturbations to preserve global diversity. A dynamic t-distribution-based elite re-exploration strategy and time-varying control parameters allow adaptive role adjustment across layers without significant computational overhead. This hierarchical design enhances WMA, maintaining fine-grained local exploitation and broad global exploration throughout iterations. Its modular structure also allows integration into other multi-layer or cooperative population-based algorithms (e.g., multi-population DE or multi-layer GWO), further improving exploration–exploitation balance and adaptability.

#### 3.2.1. Elite Layer—Gaussian Perturbation Search

Elite individuals are responsible for local exploitation. As shown in Equation (8), their update rule is:

(8) $$W_{i}^{t+1}=W_{i}^{t}+a\cdot N(0,1)⨀(g_{Best}-W_{i}^{t}),i\in N_{Elite}$$

where *N*(0,1) denotes the standard Gaussian distribution, a is an adaptive scaling factor, $N_{Elite}\approx N_{pop}/3$, and $g_{Best}$ is the global best solution after initialization. This fine-grained perturbation around the global best allows elites to refine solutions with higher precision.

#### 3.2.2. Middle Layer—Mean-Attracted Hybrid Search

Intermediate individuals adopt a “mean-guided + random sampling” strategy, balancing convergence toward leaders and random exploration. The update rule of middle-layer individuals is given in Equation (9):

(9) $$W_{i}^{t+1}=W_{i}^{t}+a\cdot rand\cdot \left(W_{Mean}-W_{i}^{t}\right)+rand\cdot (RandVec-W_{i}^{t}),i\in N_{Mid}$$

where $W_{Mean}$ is the mean position of elites, and RandVec is a random vector. With $N_{Mid}\approx N_{pop}/3$, this strategy maintains convergence toward leaders while introducing random perturbations, thereby balancing exploration and exploitation.

#### 3.2.3. Follower Layer—Differential Mutation Exploration

Follower individuals perform differential-evolution-based updates to enhance global exploration and prevent premature convergence. As shown in Equation (10), the updated rule is as follows:

(10) $$W_{i}^{t+1}=g_{Best}+F\left(x_{1}-x_{2}\right)+rand\cdot (x_{3}-W_{Mean}),\;i\;\in N_{Follower}$$

where $x_{1}$, $x_{2}$, and $x_{3}$ are randomly selected individuals, and *F* is a dynamic scaling factor. This mechanism introduces differential vectors for large-step jumps in the search space, which prevents premature convergence and strengthens global exploration.

Through this three-layer framework, IWMA establishes a cooperative structure of fine-grained search and balanced transition. A computational efficiency analysis is then conducted to examine the large-step jumps, achieving a better trade-off between global exploration and local exploitation compared with the original WMA.

### 3.3. Dynamic Adaptation and Elite Re-Exploration

To further enhance IWMA’s global exploration ability and local convergence accuracy, dynamic adaptation and elite re-exploration mechanisms are introduced. The scaling factor decreases exponentially with iterations, allowing wide exploration early and refined exploitation later, as shown in Equation (11):

(11) $$a=2\cdot e^{1-\frac{t}{T_{Max}}}$$

where *t* is the current iteration and $T_{max}$ is the maximum number of iterations. This allows IWMA to maintain a wide search radius in early stages for global exploration, while gradually narrowing toward the global optimum in later stages for refined exploitation.

To prevent monotonous search paths, the differential weight *F* oscillates sinusoidally, as given in Equation (12):

(12) $$F=0.5+0.3\cdot sin(2\pi \frac{t}{T_{Max}})$$

This periodic adjustment alternates between exploration and exploitation, preventing monotonous search paths and maintaining population diversity. Finally, Elite individuals are further perturbed according to the t-distribution. As shown in Equation (13), this allows large early-stage jumps and fine later-stage exploitation:

(13) $$W_{elite}^{t+1}=g_{Best}+t(v)\cdot g_{Best}$$

where the degrees of freedom v increase with iterations. In the early stages, the heavy-tailed property of the t-distribution [58] enables large perturbations to escape local optima, while in later stages it approximates a Gaussian distribution, providing finer exploitation near the global best.

This adaptive “coarse-to-fine” dual-phase mechanism allows elite solutions to maintain strong global exploration early on, and refined convergence in later iterations.

Through these three innovations, IWMA effectively overcomes the limitations of WMA in terms of insufficient initial diversity, simplistic search mechanisms, and a lack of dynamic control. As a result, IWMA achieves faster convergence speed and higher solution accuracy in complex optimization problems. The pseudocode of IWMA is presented in Algorithm 1.
**Algorithm 1 Pseudo-code of IWMA**Input: $N_{pop}$, $T_{max}$, *L*, *U*, *D*, f(x).Output: The global best solution $g_{Best}$ and f($g_{Best}$).
1:  Circle chaotic map initialize population *W* = {$W_{1}$, $W_{2}$,..., $W_{N}$} using Equations (6) and (7).2:  Evaluate the fitness of all whales.3:  while *t* < $T_{max}$ do4:          Sort whales by fitness in ascending order.5:          Divide population into three layers: Elite, Middle, Follower.6:          Compute mean position of elites $W_{Mean}$ (Equation (2))7:          Update adaptive parameter a using Equation (11).8:          for *i* = 1 to $N_{pop}$ do9:                  if *i* ∈ Elite layer then10:                         Update Wi using Gaussian disturbance (Equation (8)).11:                  else if *i* ∈ Middle layer then12:                         Update *W_i_* using mean−attracted mixed search (Equation (9)).13:                  else14:                         Update *W_i_* using differential mutation (Equation (10)).15:                  end if16:                  Check the bounds of $W_{i}$. (Equation (5)).17:                  Evaluate fitness f($W_{i}$).18:                  if f($W_{i}$) < f($g_{Best}$) then19:                         Update $g_{Best}$= $W_{i}$.20:                  end if21:          end for22:          Perform elite re−exploration using Student−t distribution (Equation (13)).23:          Update differential weight *F* using Equation (12).24:          Record Iter Curve(t) = f($g_{Best}$).25:          Update iteration counter *t* = *t* + 1.26:  end while27:  Return $g_{Best}$, f($g_{Best}$), IterCurve.

### 3.4. Complexity Analysis

The time complexity of the original Whale Migration Algorithm (WMA) primarily depends on the population size *N*, problem dimensionality *D*, and the maximum number of iterations *T*. Its main computational operations include population initialization, which requires *O*(*N*·*D*), fitness evaluation of all individuals in each iteration, also *O*(*N*·*D*), and position updates for both leader and follower whales, again *O*(*N*·*D*). Sorting operations performed per iteration, *O*(*N* log *N*), are comparatively negligible in large-scale problems. Consequently, the overall time complexity of WMA can be expressed as *O*(*N*·*D*·*T*).

The proposed Improved Whale Migration Algorithm (IWMA) incorporates several enhancements, including a three-layer cooperative search strategy, adaptive elite local search based on the t-distribution, and an improved Circle chaotic mapping for population initialization. Although these modifications introduce additional steps, all operations are either linear with respect to *N* and *D* or incur only minor constant overheads relative to the main iterative loop. Therefore, IWMA maintains the same asymptotic time complexity as WMA, i.e., *O*(*N*·*D*·*T*). This indicates that the improvements substantially enhance convergence behavior and global search capability without increasing the theoretical computational cost.

In subsequent sections, we will present empirical comparisons of IWMA against other benchmark algorithms on a GPU platform, highlighting both convergence performance and actual runtime efficiency to demonstrate its practical applicability in complex optimization scenarios.

## 4. Simulation and Results Analysis

The CEC2017 benchmark suite serves as the primary evaluation platform, encompassing diverse problem types and complexity levels that provide a robust foundation for assessing algorithmic convergence performance, global search capability, and robustness. Subsequently, IWMA undergoes systematic comparison against seven representative classical optimization algorithms to establish its performance superiority across multiple evaluation criteria. Statistical significance of the observed performance differentials is rigorously validated through Wilcoxon signed-rank testing to ensure reliable conclusions. Computational efficiency analysis is then conducted to examine the runtime characteristics and dimensional scalability of IWMA relative to comparative methodologies.

To elucidate the individual contributions of each enhancement strategy, comprehensive ablation experiments are performed, systematically isolating and evaluating the impact of each algorithmic component. The experimental framework concludes with a comparative assessment against contemporary migration-based algorithms, positioning IWMA within the current algorithmic landscape and highlighting its distinctive advantages within this specialized optimization paradigm. Throughout the comprehensive experimental evaluation, results are visualized through multiple representation methods, including box plots, radar charts, and integrated histogram-line plots, providing intuitive yet thorough illustrations of algorithmic performance patterns across diverse experimental settings.

### 4.1. Experimental Environment and Benchmark Tests

To validate the performance advantages of the proposed IWMA algorithm in solving complex optimization problems, the CEC2017 benchmark set was selected as the evaluation platform. This benchmark suite, proposed by the IEEE CEC conference and widely adopted in the literature, has become a unified standard in swarm intelligence and evolutionary algorithm research, providing a reliable basis for fair comparison and reproducibility.

All algorithms were executed under the Windows 10 (64-bit) operating system on hardware equipped with an Intel(R) Core(TM) i5-9300H processor (2.40 GHz) and 8 GB RAM. MATLAB R2024b was used as the software platform. This configuration ensures sufficient computational capacity for high-dimensional optimization problems while maintaining both efficiency and result stability.

The CEC2017 suite contains 29 single-objective real-parameter optimization functions (excluding F2 due to instability). These functions cover a wide range of problem types: unimodal functions (F1–F3) are simple convex problems for testing global convergence capability; multimodal functions (F4–F10) include many local optima, mainly evaluating global search ability; hybrid functions (F11–F20) combine different base functions to form medium-complexity problems for assessing adaptability in multi-feature environments; and composition functions (F21–F30) are highly complex, nonlinear, and difficult high-dimensional problems for testing robustness. All functions are subject to shifting and rotation transformations, with the search domain uniformly set to ${\left[-100,100\right]}^{d}$, forcing algorithms to explore a large solution space for comprehensive evaluation.

### 4.2. Comparison with Mainstream Optimization Algorithms

To evaluate the convergence speed, global search ability, and robustness of IWMA, comparative experiments were conducted on the CEC2017 benchmark set. Eight algorithms were tested, including WMA, PSO, GWO, GTO, IVY, DBO, and L-SHADE. For fair comparison, the population size for all algorithms was set to 30, and the maximum iterations to 1000, ensuring the same number of fitness evaluations. Each experiment was repeated 30 times. Problems with dimensions 30 and 50 were used to assess both standard and high-dimensional performance. Experimental parameters are summarized in Table 1.

**Figure 1:**
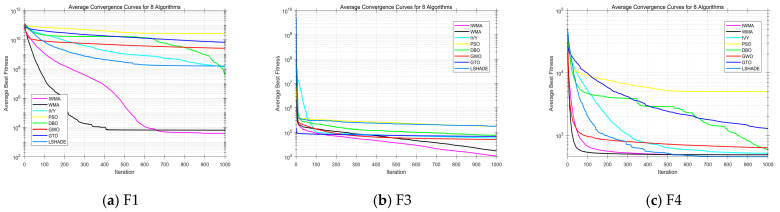

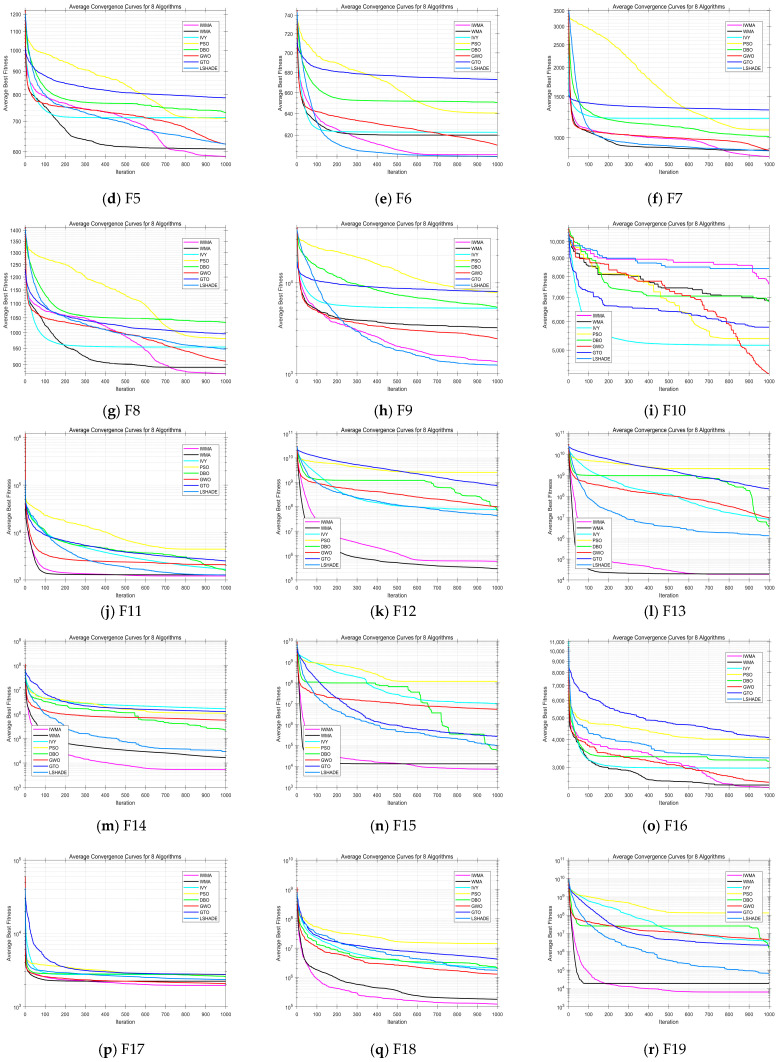

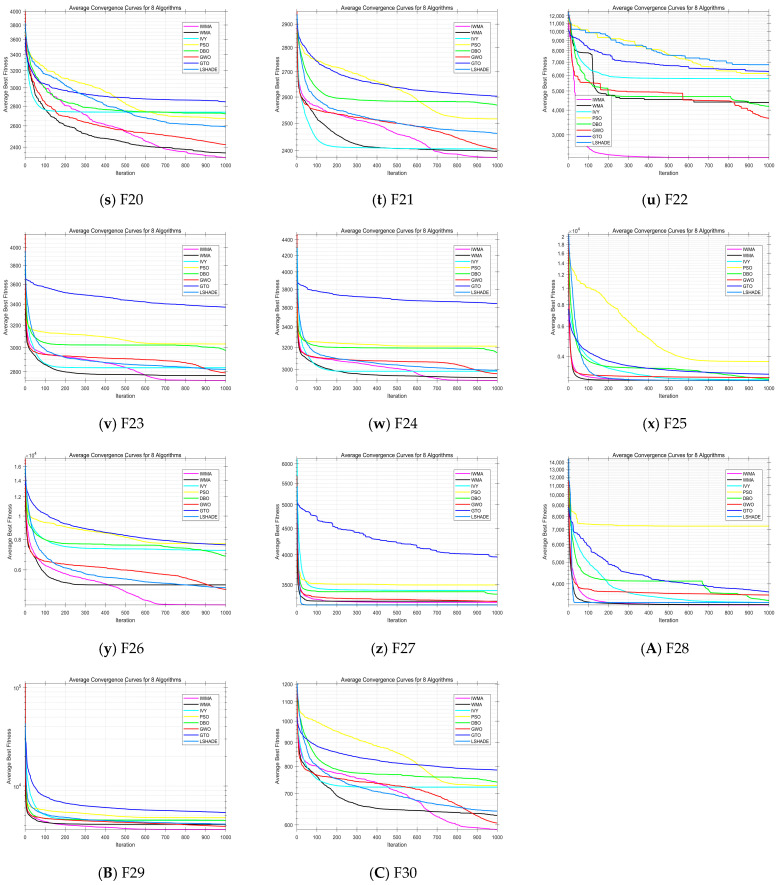
Convergence curves of the algorithms on test functions in 30 dimensions.

As shown in Figure 1 and Table 2, IWMA demonstrates clear superiority across the 30-dimensional CEC2017 benchmark functions. In terms of key metrics, IWMA dominates most functions, achieving the best value in 27 out of 29 functions, the best mean in 25 functions, and the lowest standard deviation in 23 functions. While a few functions show slightly better performance by other algorithms, such as LSHADE, GWO, WMA, and IVY, these exceptions are limited, and IWMA maintains a clear advantage across the majority of test problems.

The convergence curves in Figure 1 further highlight IWMA’s performance. Across most functions, IWMA converges faster and more smoothly than its competitors, reflecting efficient exploration and stable exploitation. For instance, on F3, IWMA quickly reaches high-quality solutions, with a mean value far superior to other algorithms. On the multimodal function F4, LSHADE achieves a slightly lower mean value than IWMA, but IWMA still outperforms the remaining competitors. Even in functions where other algorithms occasionally excel, IWMA maintains competitive convergence and reliable performance throughout the iterations.

Overall, IWMA shows excellent convergence, solution quality, and robustness, consistently dominating most functions as indicated by numerical results and convergence curves, providing a solid basis for evaluation on higher-dimensional problems.

As shown in Table 3, IWMA continues to demonstrate strong overall performance in the 50-dimensional CEC2017 benchmark tests.

For the Best indicator, IWMA achieves the best results on 25 out of 29 functions, with slightly lower performance on F1, F10, F12, and F27. These minor differences do not compromise IWMA’s overall dominance.

Regarding the Mean indicator, IWMA maintains superiority on 18 functions. Although other algorithms achieve better mean values on some functions, IWMA exhibits excellent convergence accuracy on the remaining functions, particularly on complex high-dimensional problems.

For the Std indicator, IWMA demonstrates strong stability, achieving the best results on 18 functions. While a few functions show slightly better stability for other algorithms, IWMA consistently maintains low variance across the majority of functions.

Combining the results from the 30-dimensional and 50-dimensional CEC2017 benchmark functions, IWMA demonstrates outstanding overall performance. In the 30-dimensional experiments, IWMA achieves the best value in 27 out of 29 functions, the best mean in 25 functions, and the lowest standard deviation in 23 functions, indicating its comprehensive superiority in convergence accuracy, solution quality, and stability. Although a few functions show slightly better performance by other algorithms such as LSHADE, GWO, WMA, and IVY, these exceptions are limited and do not undermine IWMA’s overall dominance across the majority of test problems.

In the 50-dimensional high-dimensional setting, IWMA continues to maintain strong performance. It achieves the best results on 25 out of 29 functions, the best mean on 18 functions, and the lowest standard deviation on 18 functions. While some functions show marginally better performance by other algorithms in terms of mean or standard deviation, IWMA consistently maintains low variance and excellent convergence on the remaining functions, highlighting its robustness and reliability in high-dimensional, complex problems.

The convergence curves further emphasize IWMA’s efficiency. Across most functions, IWMA converges faster and more smoothly than its competitors, reflecting a well-balanced mechanism between global exploration and local exploitation. Even in functions where other algorithms occasionally perform better, IWMA maintains competitive convergence and reliable performance throughout the iterations. Notably, in complex functions such as F12, F15, and F16, IWMA significantly outperforms most competitors, demonstrating strong global search capability and precise convergence in high-dimensional optimization.

To compare the performance of IWMA with six other algorithms on the CEC2017 benchmark functions, radar charts and combined histogram–line plots were used to visualize overall and average performance. For the radar charts, the mean values from Table 2 and Table 3 were normalized per function by dividing the minimum mean among the eight algorithms by each algorithm’s mean. This scales the scores between 0 and 1, where higher values indicate better performance, effectively highlighting the relative strengths of each algorithm. The combined histogram–line plots were generated based on the aggregated average rankings of all eight algorithms across the 29 test functions, with smaller ranks representing superior performance.

**Figure 2:**
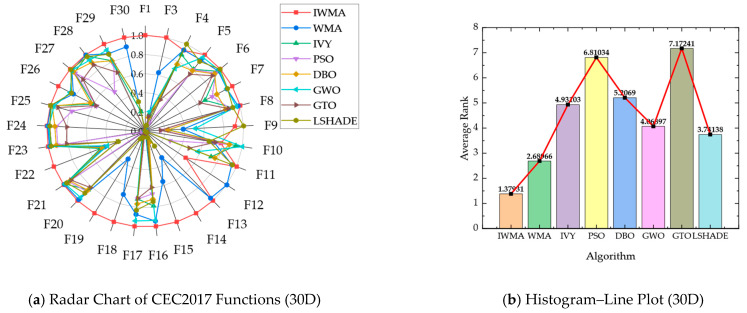
Performance comparison at 30D.

**Figure 3:**
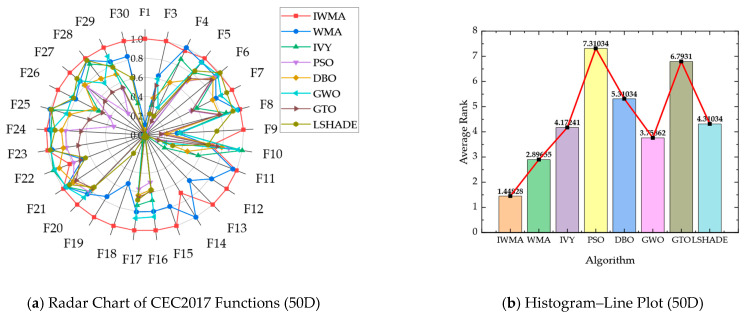
Performance comparison at 50D.

Figure 2 and Figure 3 present these visualizations for the 30-dimensional and 50-dimensional cases, respectively, allowing clear comparison of algorithm performance across different dimensionalities and problem complexities:

In the 30-dimensional scenario, as shown in Figure 2, IWMA demonstrates balanced and stable performance across all test functions. The radar chart indicates that IWMA consistently lies near the outer boundary for most benchmark functions, reflecting its robust performance across diverse problems. Quantitatively, IWMA achieves the best average rank of 1.38, followed by WMA at 2.69 and L-SHADE at 3.71, whereas PSO and GTO are the weakest performers, with average ranks of 6.81 and 7.17, respectively. The combination of radar chart visualization and quantitative ranking confirms that IWMA not only surpasses all competitors in terms of average performance but also maintains a high degree of robustness and consistency across different functions, as evidenced by its balanced radar profile and superior ranking metrics. Furthermore, in Figure 2b, the red line and black square are incorporated as supplementary graphical elements to the histogram. Specifically, the red line illustrates the overall performance trend, while the black square serves to indicate the IWMA algorithm.

The radar chart’s consistent outer-boundary positioning indicates that IWMA maintains stable performance across diverse functions, minimizing the risk of degradation in complex, high-dimensional search spaces. Low variance in average ranks confirms that its performance is uniformly robust, rather than dominated by simpler functions. Combining visual and numerical evidence, this analysis highlights IWMA’s strengths and provides a solid basis for evaluations on higher-dimensional benchmarks. Overall, IWMA effectively balances convergence speed, solution quality, and robustness, demonstrating its potential as a reliable optimization tool for complex, high-dimensional problems.

In the 50-dimensional case, as shown in Figure 3, the advantage of IWMA is further strengthened. The radar chart demonstrates that IWMA consistently reaches the outer boundary across the majority of functions, indicating more reliable convergence compared to its competitors. The histogram–line plot provides a quantitative comparison: IWMA achieves the best average rank of 1.44, far ahead of WMA with 2.89 and GWO with 3.56, while IVY, L-SHADE, and DBO occupy the middle tier with average ranks of 4.17, 4.31, and 5.10, respectively. In contrast, PSO and GTO perform the worst, with average ranks of 7.31 and 6.79. These results clearly demonstrate that IWMA maintains outstanding global search capability and robustness in high-dimensional optimization tasks. Overall, whether on low- or high-dimensional test functions, IWMA exhibits strong adaptability, efficient search ability, and reliable global optimization performance, making it a consistently superior choice for solving complex optimization problems.

To balance representativeness and conciseness, eight representative functions from the CEC2017 benchmark set are selected for comparison under different dimensional settings. In the 30-dimensional scenario, the chosen functions include unimodal functions F1 and F3, multimodal functions F5 and F6, hybrid functions F11 and F17, and composite functions F21 and F23, thereby covering diverse problem types. In the 50-dimensional scenario, the selected functions are F3, F4, F17, F24, F25, F27, F29, and F30, which are characterized by higher complexity and thus better reflect the challenges of high-dimensional optimization. This selection ensures that both simple and complex landscapes are considered, allowing a comprehensive assessment of the algorithm’s scalability, convergence stability, and adaptability. Such a design not only encompasses various categories of functions but also highlights the performance differences of IWMA across different dimensions and complexities, providing an intuitive and rigorous basis for subsequent performance analysis.

**Figure 4:**
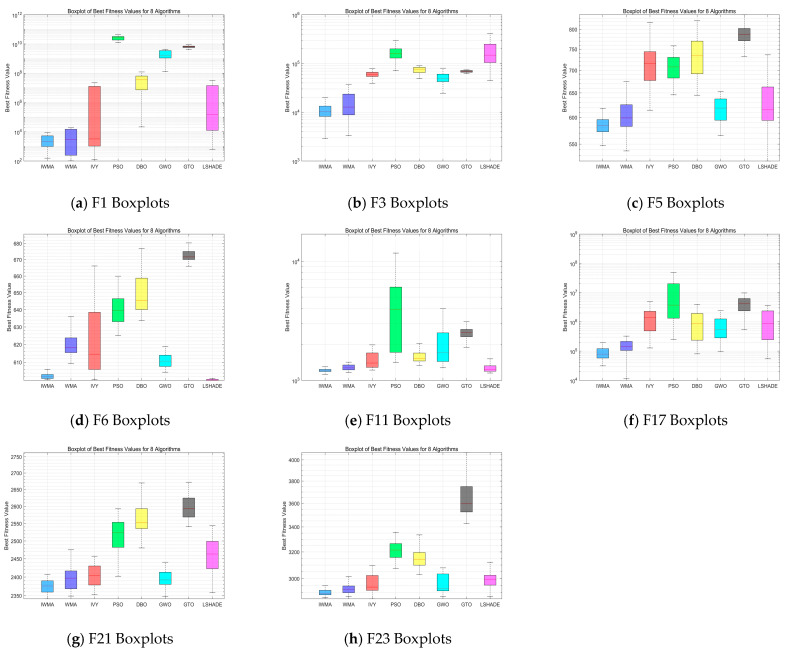
Boxplots of Representative CEC2017 Functions (30D).

Figure 4 presents the boxplot results of IWMA and seven comparative algorithms on the 30-dimensional CEC2017 test functions. As shown, IWMA not only achieves lower median values on most functions but also exhibits significantly narrower box ranges, indicating superior optimization accuracy and result stability compared to the other algorithms. Particularly on unimodal and hybrid functions, IWMA demonstrates higher concentration and fewer outliers, suggesting a good balance between convergence speed and stability. In contrast, some algorithms, such as WMA and LSHADE, although performing close to IWMA on certain low-dimensional unimodal functions, generally show higher medians and wider distributions, reflecting weaker stability.

**Figure 5:**
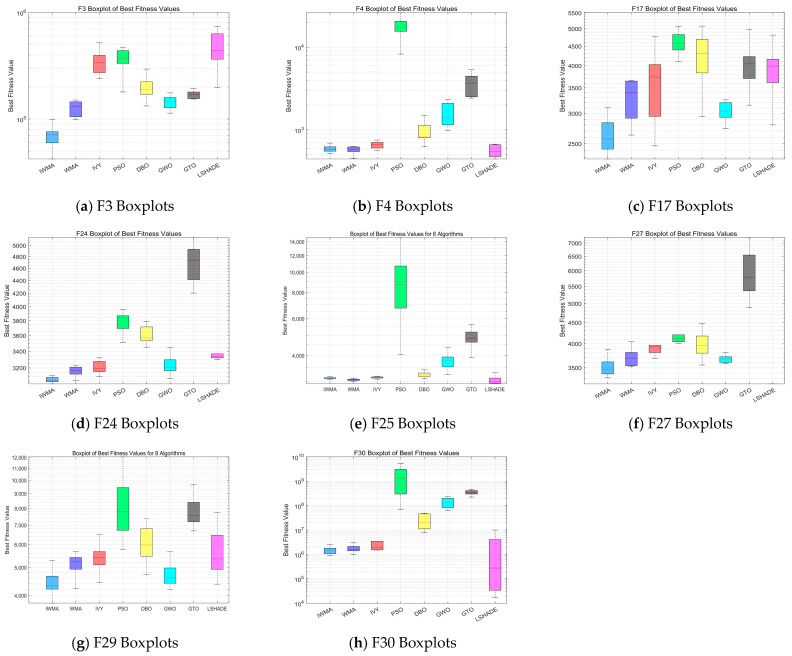
Boxplots of representative CEC2017 functions (50D).

Figure 5 shows the boxplot comparison in the 50-dimensional scenario. With the increase in dimensionality, problem complexity rises significantly, and some comparative algorithms exhibit unstable convergence and scattered results in high-dimensional environments. IWMA, however, continues to maintain lower medians and tighter distributions. Its advantages are particularly pronounced in complex hybrid and composite functions, highlighting its robustness and global search capability in high-dimensional optimization problems. Although WMA and LSHADE can still achieve performance close to IWMA on a few functions, their overall convergence accuracy and stability remain inferior.

In summary, IWMA demonstrates clear advantages in both 30-dimensional and 50-dimensional tests, further validating its applicability and competitiveness in complex optimization problems across different dimensions, The horizontal lines represent the median values of multiple runs for each algorithm.

Overall, by integrating the results from both 30-dimensional and 50-dimensional experiments, it is evident that IWMA consistently excels in terms of solution quality, stability, and extreme-case performance. Its advantages become even more pronounced as the problem dimensionality increases, confirming the algorithm’s scalability, adaptability, and competitiveness. Based on these promising results, we further conduct the Wilcoxon test comparison to statistically quantify the performance differences between IWMA and the comparative algorithms.

### 4.3. Wilcoxon Test Comparison

To establish rigorous statistical validation of the experimental findings, the Wilcoxon signed-rank test was implemented to assess the statistical significance of performance disparities between IWMA and eight comparative algorithms: PSO, DBO, GWO, GTO, WMA, IVY, and LSHADE. This non-parametric statistical test evaluates pairwise algorithmic performance differences, where p-values below the significance threshold of 0.05 indicate statistically significant performance disparities, whereas p-values exceeding this threshold suggest statistically insignificant differences.

Table 4 and Table 5 present the comprehensive Wilcoxon test results for 30-dimensional and 50-dimensional CEC2017 benchmark functions, respectively. Each tabulated value represents the computed *p*-value derived from pairwise comparisons between IWMA and the corresponding comparative algorithm across individual test functions, thereby facilitating systematic assessment of performance differentials. Statistically significant differences (*p* < 0.05) are denoted in bold typeface to provide clear visual identification of algorithmically meaningful performance distinctions across the benchmark suite.

As shown in Table 4 and Table 5, the Wilcoxon signed-rank test provides strong statistical evidence of IWMA’s superiority over the comparative algorithms on both the 30D and 50D CEC2017 benchmark functions. Most pairwise comparisons yield *p*-values well below 0.05, indicating that IWMA’s performance improvements are statistically significant rather than due to random variation. In particular, IWMA consistently outperforms PSO, DBO, GWO, GTO, and LSHADE, while also showing significant advantages over the stronger baselines WMA and IVY in the majority of cases. The number of non-significant results decreases as dimensionality increases from 30D to 50D, further highlighting IWMA’s stability and robustness in high-dimensional search spaces. These statistical findings complement earlier analyses based on mean values, boxplots, and radar charts, providing comprehensive validation of IWMA’s effectiveness. Moreover, the Wilcoxon test confirms that these improvements are consistent across different function types, including unimodal, multimodal, hybrid, and composition functions, demonstrating IWMA’s adaptability and reliability in diverse optimization landscapes. Collectively, the combination of numerical, graphical, and statistical analyses provides a solid foundation for considering IWMA as a competitive and robust algorithm for high-dimensional optimization tasks.

### 4.4. Computational Time Comparison

Beyond solution quality and stability performance, computational efficiency constitutes a critical dimension for evaluating the comprehensive performance of optimization algorithms, particularly within high-dimensional optimization contexts. Building upon the preceding performance analysis of IWMA against seven comparative algorithms across 30-dimensional and 50-dimensional CEC2017 test functions, the average execution times of each algorithm were systematically recorded and analyzed. Table 6 and Table 7 present the computational runtime statistics for IWMA under 30-dimensional and 50-dimensional scenarios, respectively, providing essential benchmarks for practical algorithmic implementation and deployment considerations.

**Figure 6:**
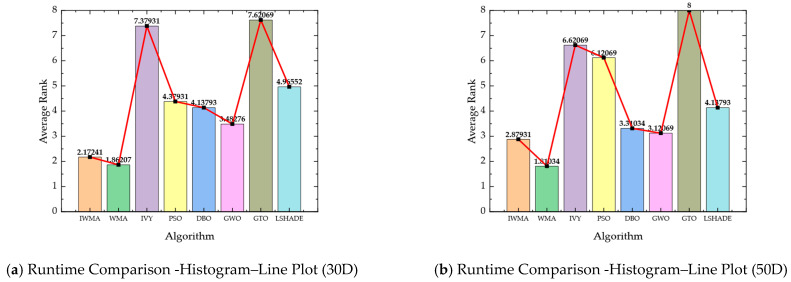
Runtime Comparison.

As shown in Table 6 and Table 7, together with the histogram–line plots in Figure 6. IWMA exhibits nearly identical computational times to the baseline WMA. The average overhead is approximately 7% in 30D and 12% in 50D, while the corresponding median values are even smaller (6% and 3%, respectively). This confirms that the three proposed strategies—circle chaotic initialization, the three-layer cooperative search framework, and the adaptive re-exploration mechanism—introduce no additional complexity burden. IWMA therefore maintains the same overall computational order O(P·D·T) as WMA.

From a comparative perspective, IWMA requires significantly less runtime than IVY and GTO, while remaining at a comparable level to PSO, DBO, GWO, and L-SHADE. As illustrated by the histogram–line plots, IWMA consistently ranks close to WMA, highlighting its lightweight nature, whereas IVY and GTO incur the highest computational costs. These findings confirm that the improved convergence of IWMA stems from the synergistic interplay of its strategies rather than from increased time complexity.

### 4.5. Ablation Experiments

To systematically investigate the individual contributions of each enhancement strategy within the IWMA framework, ablation experiments were conducted. The proposed IWMA algorithm incorporates three key strategies: (1) circle chaotic initialization strategy for enhancing population diversity, (2) three-layer cooperative search strategy for balancing global exploration and local exploitation, and (3) dynamic adaptive mechanism combined with elite re-exploration strategy for stabilizing convergence.

Multiple algorithm variants were constructed by selectively including or excluding each strategy to evaluate their respective impacts on performance and to examine potential synergistic effects. Table 8 summarizes the strategy combinations for each variant, where “O” denotes inclusion of a given strategy and “X” denotes exclusion. For example, IWMA1 only includes the first strategy, while IWMA12 includes both the first and second strategies. The full IWMA integrates all three strategies, representing the complete algorithm design.

**Figure 7:**
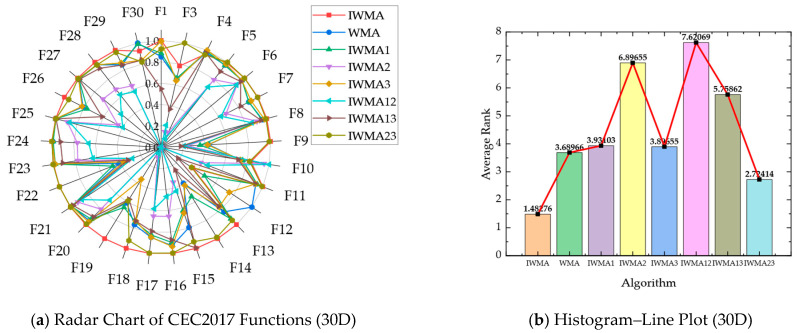
Performance comparison and ablation study at 30D.

The subsequent Table 9 and Table 10 present the detailed numerical results of the ablation experiments conducted on the 30D and 50D CEC2017 benchmark functions, respectively. These tables provide a systematic comparison of each variant in terms of convergence accuracy, solution stability, and robustness, facilitating a comprehensive assessment of individual strategy contributions. Moreover, the synergistic effects of integrating multiple strategies within the full IWMA framework are highlighted, demonstrating how the combined mechanisms enhance both exploration and exploitation. Complementing the numerical analysis, radar and histogram visualizations are provided to intuitively illustrate performance improvements, offering an interpretable depiction of how each component collectively reinforces the overall efficacy of the proposed algorithm. Table 9 and Figure 7 show IWMA’s superior performance on 30-dimensional CEC2017 problems, with an average Friedman ranking of 1.49, consistently outperforming all ablation variants. IWMA23 ranks 2.74, highlighting the importance of Circle chaotic initialization in enhancing population diversity, improving search coverage, and providing a solid foundation for high-dimensional optimization.

**Figure 8:**
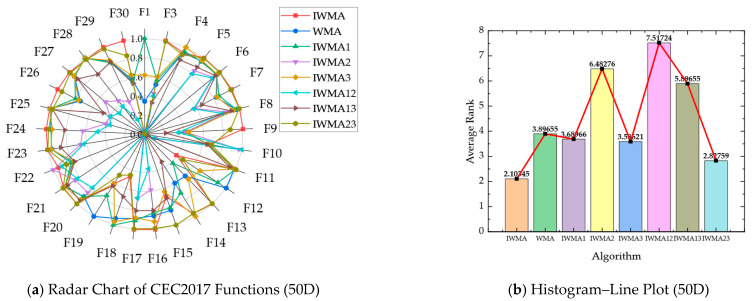
Performance comparison and ablation study at 50D.

Similarly, Table 10 and Figure 8 confirm IWMA’s dominance on 50-dimensional problems, with an average ranking of 2.10 compared to 2.64 for IWMA23. These results further highlight the pivotal contribution of Circle chaotic initialization in establishing a robust and efficient algorithmic framework for high-dimensional optimization tasks.

Component-wise performance analysis elucidates dimension-dependent behavioral characteristics across individual strategies. Circle chaotic initialization manifests contrasting efficacy patterns, yielding an average ranking of 3.92 in 30-dimensional instances (marginally inferior to WMA’s 3.66), while achieving substantial improvement to 3.66 in 50-dimensional scenarios (surpassing WMA’s 3.90). This phenomenon indicates that quasi-uniform distribution properties become increasingly critical for preventing premature convergence within high-dimensional search manifolds. The adaptive re-exploration mechanism demonstrates consistent robustness across dimensional scales, securing rankings of 3.86 and 3.26 for 30-dimensional and 50-dimensional problems, respectively, thereby validating its indispensable role in facilitating local optima evasion within complex multimodal and composite function topologies. Conversely, the three-layer cooperative search framework exhibits suboptimal standalone performance, achieving rankings of 6.99 and 6.45 across respective dimensional contexts. The hybrid configurations IWMA12 and IWMA13 similarly demonstrate inferior performance, frequently underperforming the baseline WMA. Nevertheless, synergistic integration with the adaptive mechanism precipitates substantial performance enhancement, revealing pronounced complementary effects.

The comprehensive ablation analysis conclusively establishes that IWMA’s algorithmic superiority emanates from the orchestrated integration of three complementary strategies. Circle chaotic initialization furnishes diversified and spatially well-distributed initial population configurations, the three-layer cooperative architecture maintains dynamic exploration-exploitation equilibrium, and the adaptive re-exploration mechanism optimizes both global escape mechanisms and local convergence refinement processes. This hierarchical integration enables IWMA to consistently achieve reduced error magnitudes, minimized solution variance, and optimal performance rankings across both dimensional scales, thereby quantitatively corroborating the instrumental role of Circle chaotic initialization in amplifying overall optimization efficacy.

### 4.6. Performance Comparison of IWMA and Migration-Inspired Algorithms

Subsequent to the comprehensive benchmarking against classical optimization paradigms and rigorous statistical validation, this investigation extends to evaluating IWMA’s performance against cutting-edge migration-inspired metaheuristic algorithms. The migration-based optimization paradigm has undergone substantial algorithmic evolution, yielding several distinguished methodologies including WMA, ALA, GGO, WaoA, and SOA, which collectively represent the current state-of-the-art in bio-inspired migration mechanisms for complex optimization scenarios.

**Figure 9:**
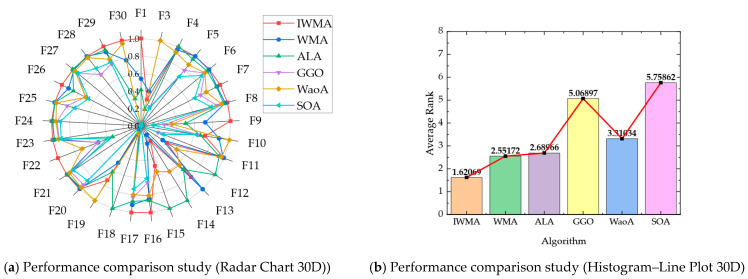
Performance comparison study at 30D.

**Figure 10:**
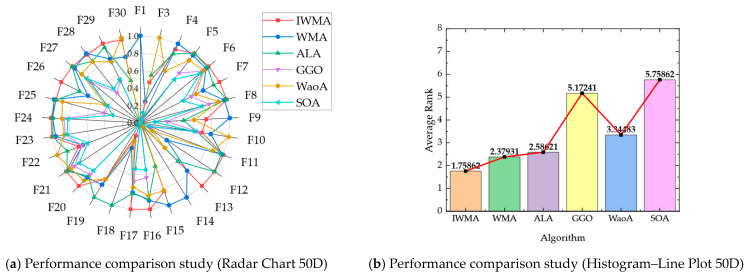
Performance comparison study at 50D.

Empirical assessments are systematically conducted utilizing the CEC2017 benchmark suite across dual-dimensional configurations of 30-dimensional and 50-dimensional optimization landscapes. The comparative analysis employs sophisticated visualization methodologies, incorporating radar chart representations to delineate function-specific performance characteristics and integrated histogram-line graphical analyses to quantify average ranking distributions across the algorithmic ensemble. This multi-faceted analytical approach facilitates comprehensive performance differentiation and establishes definitive algorithmic superiority within the migration-based optimization domain, as illustrated in Figure 9 and Figure 10.

As illustrated in Figure 9, the comparative evaluation against contemporary migration-based algorithms on 30-dimensional CEC2017 benchmark functions demonstrates IWMA’s pronounced algorithmic superiority. The radar chart visualization reveals that IWMA consistently maintains proximity to the optimal center across the majority of test functions, exhibiting superior convergence characteristics compared to alternative migration-inspired methodologies. Quantitative analysis indicates that IWMA achieves the optimal average ranking of 1.62, with WMA securing second position at 2.55, followed by ALA at 2.69, WaoA at 3.31, GGO at 5.07, and SOA at 5.76.

Figure 10 presents the algorithmic performance under high-dimensional optimization scenarios. In 50-dimensional problems, IWMA maintains its leading position with an average ranking of 1.76, while WMA achieves 2.38. Notably, as dimensionality increases, the performance hierarchy remains stable with ALA at 2.59, WaoA at 3.34, GGO at 5.17, and SOA at 5.76. The radar chart demonstrates that IWMA sustains robust convergence performance in high-dimensional environments, validating the algorithm’s dimensional scalability characteristics.

The consistent cross-dimensional results empirically validate the selection of WMA as the primary baseline comparator, as it consistently ranks second across both dimensional configurations, establishing its authoritative position within the migration-based optimization paradigm. IWMA demonstrates substantial performance improvements over WMA, achieving 36.5% and 26.1% ranking enhancements in 30-dimensional and 50-dimensional scenarios, respectively. This sustained cross-dimensional superiority conclusively validates the effectiveness of the proposed Circle chaotic initialization, three-layer cooperative search framework, and dynamic adaptive mechanisms. The stable performance advantages across varying problem complexities indicate that the enhancement strategies not only augment optimization capabilities but also ensure robust performance across diverse computational challenges.

This chapter establishes a comprehensive experimental evaluation framework that rigorously demonstrates IWMA’s algorithmic excellence across multifaceted performance criteria. Extensive empirical investigations conducted on the CEC2017 benchmark suite reveal IWMA’s consistent superiority over both classical optimization paradigms and contemporary migration-based metaheuristic methodologies across 30-dimensional and 50-dimensional optimization landscapes. Statistical validation through Wilcoxon signed-rank analysis substantiates the significance and robustness of the observed performance improvements, while computational complexity assessment confirms that IWMA’s sophisticated enhancement mechanisms deliver exceptional optimization efficacy without compromising computational tractability.

The ablation study provides critical insights into the intricate synergistic relationships among three fundamental components: circle chaotic initialization, three-layer cooperative search architecture, and dynamic adaptive mechanism integrated with elite re-exploration strategy. These components demonstrate complementary interactions that collectively amplify algorithmic performance beyond their individual contributions. Comparative benchmarking against cutting-edge migration-inspired algorithms further validates the algorithmic design rationale and empirically justifies the strategic selection of WMA as the foundational baseline.

IWMA’s sustained competitive advantages across heterogeneous function topologies, scalable dimensional complexities, and rigorous benchmarking protocols establish its position as a premier optimization methodology within the contemporary algorithmic landscape. The comprehensive experimental validation presented herein provides a robust theoretical foundation for IWMA’s deployment in practical engineering optimization scenarios, particularly demonstrating strong empirical evidence for its effectiveness in addressing complex multi−constrained optimization challenges requiring sophisticated solution strategies.

## 5. Multi−UAV Cooperative 3D Path Planning

In complex spatial environments, path planning plays a vital role in UAV mission execution, as it directly affects both flight safety and operational efficiency. Moreover, it must account for multiple mission-specific constraints, including environmental hazards, resource limitations, and task requirements. To address these challenges, this study develops a multi-UAV three-dimensional path planning model to verify the applicability and advantages of the proposed algorithm in cooperative mission scenarios. In particular, the model emphasizes realistic operational factors such as obstacle avoidance, threat region constraints, and formation maintenance, ensuring that the generated trajectories are not only mathematically optimized but also practical and feasible for real-world deployment.

### 5.1. Multi-UAV Path Planning Modeling

In multi-UAV path planning problems, several constraints must be considered to ensure that the generated paths meet practical application requirements. These include flight energy consumption, threat avoidance, flight altitude, path smoothness, time window limitations, and collision avoidance in space. Based on these requirements, this study designs a comprehensive objective function to optimize UAV flight paths. The objective function consists of multiple sub-objectives, which are weighted and summed according to task demands to minimize the overall cost of path planning.

#### 5.1.1. Path Energy Cost

The total energy consumption of a flight path is an important metric in path planning, representing the total energy expended by a UAV traveling from the start point to the destination. This cost is computed by considering both the horizontal distances and the vertical altitude differences between successive waypoints. The formulation is as follows:

(14) $$f_{1}=\sum_{i=1}^{N}\sum_{k=1}^{M-1}\left(\left\|p_{i,k+1}-p_{i,k}\right\|+3\times \left|z_{i,k+1}-z_{i,k}\right|\right)$$

In Equation (14), $p_{i,k}$ denotes the *k*, the waypoint of ${UAV}_{i}$. The term $\left\|p_{i,k+1}-p_{i,k}\right\|$ represents the Euclidean distance between two consecutive waypoints, while $\left|z_{i,k+1}-z_{i,k}\right|$ represents the vertical altitude change. Since vertical flight typically requires more energy, a weighting factor of 3 is applied.

#### 5.1.2. Path Threat Cost

Threat regions are divided into two types: the single-UAV path-segment model $f_{2}$ for evaluating risks between trajectory segments and obstacles, and the multi-UAV waypoint threat-sphere model $f_{3}$ for assessing waypoint-level risks in formations. The following sections provide a detailed description of each model.

**Figure 11:**
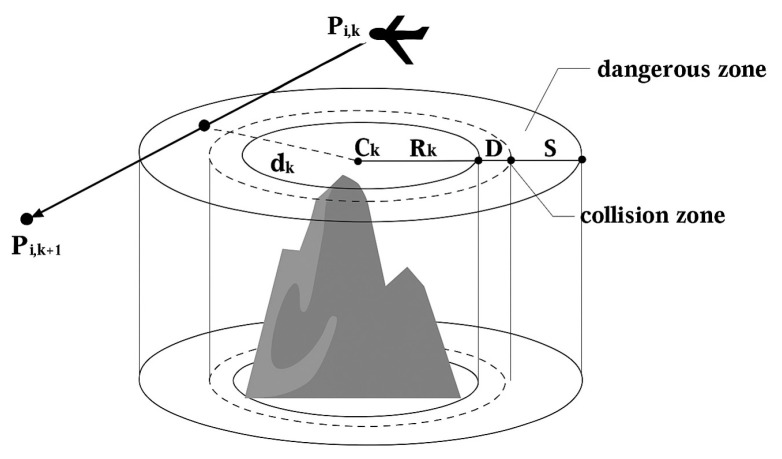
Illustration of threat region division.

In complex flight environments, UAVs must avoid collisions with static obstacles or airborne threats to ensure flight safety. These obstacles and threats are typically represented by cylindrical or spherical regions that define safety constraints. Assume there are K obstacles in the environment, each represented by a cylindrical region. The horizontal projection of obstacle k has center coordinates $C_{k}$ and radius $R_{k}$ (*k* = 1, 2, …, *K*) Each UAV has a safety radius D and must maintain a safety margin S from obstacles. The division of threat regions is illustrated in Figure 11.

When a UAV’s path segment $P_{i,k+1}P_{i,k}$ passes through the projection of an obstacle, the minimum distance from the path segment to the obstacle center is denoted as $d_{k}$. Based on this distance, the threat cost function $B_{k}$ can be defined as follows:

(15) $$B_{k}\left(P_{i,k+1}P_{i,k}\right)=\left\{\begin{array}{c} 0\;\;\;\;\;\;\;\;\;\;\;\;\;\;\;\;\;\;\;\;\;\;\;\;\;\;\;\;\;ifd_{k}>R_{k}+S+D\;\;\;\;\;\;\;\;\;\;\;\;\;\;\;\;\;\;\;\\ \frac{R_{k}+S+D-d_{k}}{S}\;\;\;\;\;\;\;\;\;\;\;\;\;ifD+R_{k}<d_{k}\leq R_{k}+S+D\;\\ 1{\;\;\;\;\;\;\;\;\;\;\;\;\;\;\;\;\;\;\;\;\;\;\;\;\;\;\;\;\;ifd}_{k}\leq R_{k}+D\;\;\;\;\;\;\;\;\;\;\;\;\;\;\;\;\;\;\;\;\;\;\;\;\;\;\;\; \end{array}\right.$$

In Equation (15), $B_{k}\left(P_{i,k+1}P_{i,k}\right)$ denotes the threat cost of ${UAV}_{i}$ on path segment ($P_{i,k+1}P_{i,k}$) with respect to obstacle k. Here, $d_{k}$ is the minimum distance from the path segment to the obstacle center, $R_{k}$ is the obstacle radius, *D* is the UAV safety radius, and *S* is the safety buffer distance.

(1) If $d_{k}\leq R_{k}+D$, the UAV has entered the collision region and is considered destroyed, thus $B_{k}$ = 1.
(2) If $D+R_{k}<d_{k}\leq R_{k}+S+D$, the UAV is in a danger zone but not in collision, and the threat cost decreases inversely with distance, expressed as $\frac{R_{k}+S+D-d_{k}}{S}$.
(3) If $d_{k}>R_{k}+S+D$, the UAV is outside the obstacle’s influence, and the threat cost is set to 0.

In summary, the threat cost of an entire flight path under all obstacles can be obtained by cumulatively summing the threat costs of each path segment in different threat regions, as shown in Equation (16):

(16) $$f_{2}=\sum_{j=1}^{n-1}\sum_{k=1}^{K}B_{k}\left(P_{i,k+1}P_{i,k}\right)$$

For ${UAV}_{i}$ at waypoint $p_{i,k}$, if it enters the *j*-the threat sphere, the corresponding distance is denoted as $d_{i,k}^{j}$. The cost function takes different forms depending on the region.

For Region 1, let the constant be $p_{31}$, and the corresponding cost is given by $c_{i,k}^{j}=\frac{p_{31}}{{{(d}_{i,k}^{j})}^{4}}$. In Region 2, if the sphere radius is $R_{a,j}$, the cost is calculated as $c_{i,k}^{j}=\frac{{{(R}_{a,j})}^{4}}{{{{{(R}_{a,j})}^{4}+(d}_{i,k}^{j})}^{4}}$. The total threat cost of ${UAV}_{i}$ is obtained by accumulating all costs across the threat regions, expressed as $f_{t,i}=\sum_{k=1}^{m_{j}}\sum_{j\in T_{i}(k)}c_{i,k}^{j}$, where $T_{i}(k)$ denotes the set of threat regions in which $\;{UAV}_{i}$ is located at waypoint *k*. Extending this to the formation level, the total threat cost can be written as Equation (17):

(17) $$f_{3}=\sum_{i=1}^{N}f_{t,i}$$

Table 11 compares the main characteristics of the single UAV path segment threat model and the multi-UAV waypoint threat sphere model. The former evaluates threats for individual UAVs at the path segment level, using 2D obstacle projections, while the latter assesses threats at UAV waypoints within a 3D threat sphere framework and aggregates the cost across all UAVs in the formation. Both models capture the principle that threat cost increases as UAVs approach obstacles or threat regions, but they differ in granularity and application scope.

This threat cost model effectively characterizes the spatial relationship between UAVs and surrounding obstacles. As the flight path approaches an obstacle, the cost rises significantly, guiding the optimization process to adaptively avoid potential threats. This ensures that flight missions are completed while strictly satisfying safety constraints.

#### 5.1.3. Path Altitude Cost

In complex environments, UAV flight altitude is typically restricted within a certain range. For example, in reconnaissance missions, the UAV should not exceed the maximum altitude determined by camera resolution. At the same time, to avoid ground-based weapons and unnecessary altitude fluctuations due to terrain, the UAV should not fly below a specified minimum altitude.

**Figure 12:**
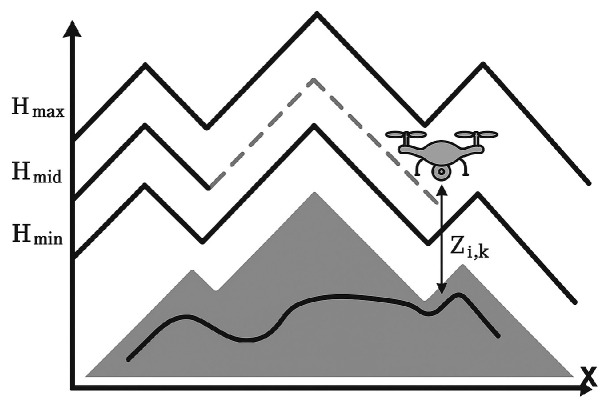
Flight altitude illustration.

The maximum and minimum altitudes are denoted as $H_{max}$ and $H_{min}$, respectively. The reference altitude $H_{mid}$ is defined in Equation (18):

(18) $$H_{mid}=\frac{H_{max}+H_{min}}{2}$$

which represents the ideal flight altitude of ${UAV}_{i}$, as illustrated in Figure 12.

During path optimization, an altitude penalty function $h_{i}(k)$ is introduced. If the waypoint altitude $z_{i,k}$ lies within the allowable range $\left[H_{min},H_{max}\right]$, the cost is defined as the relative deviation from the reference altitude. Otherwise, the cost is set to 1, as in Equation (19):

(19) $$h_{i}(k)=\left\{\begin{array}{ll} \left|\frac{z_{i,k}-H_{mid}}{H_{mid}}\right| & {ifH}_{min}{<z}_{i,k}{<H}_{max}\\ 1 & \mathrm{otherwise} \end{array}\right.$$

By summing the costs across all nodes along the flight path, the overall altitude constraint model can be expressed as Equation (20):

(20) $$f_{4}=\sum_{i=1}^{N}\sum_{k=1}^{m_{i}}h_{i}(k)$$

#### 5.1.4. Path Smoothness Cost

To ensure the continuity and feasibility of flight trajectories, UAV path planning must limit sharp maneuvers in both the horizontal and vertical directions. For this purpose, the horizontal turning angle and climbing angle are introduced as smoothness metrics.

The horizontal turning angle $∆\emptyset_{i}(k)$ measures the degree of turning between consecutive waypoints on the horizontal plane. It is calculated as the angle between vectors $P_{i,k}^{\prime }P_{i,k+1}^{\prime }$ and $P_{i,k+1}^{\prime }P_{i,k+2}^{\prime }$, as shown in Equation (21):

(21) $$∆\emptyset_{i}\left(k\right)=acrtan\left(\frac{\left\|P_{i,k}^{\prime }P_{i,k+1}^{\prime }\times P_{i,k+1}^{\prime }P_{i,k+2}^{\prime }\right\|}{P_{i,k}^{\prime }P_{i,k+1}^{\prime }\cdot P_{i,k+1}^{\prime }P_{i,k+2}^{\prime }}\right)$$

**Figure 13:**
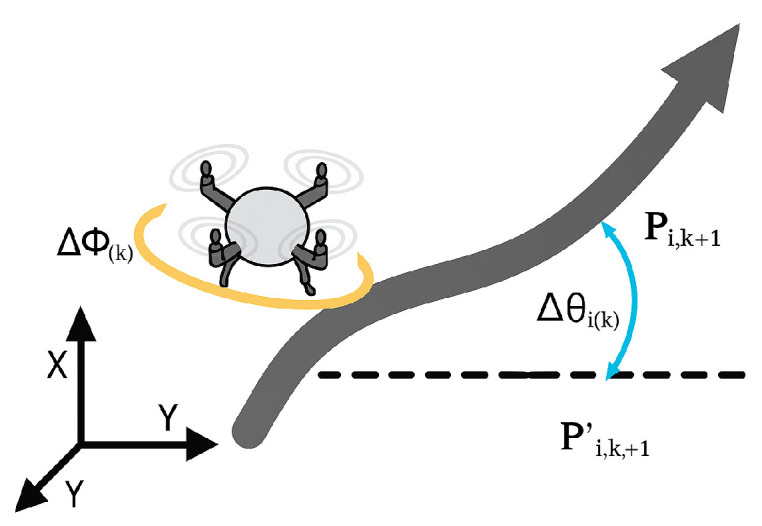
Path Smoothness Cost.

As illustrated in Figure 13, the climbing angle $∆\theta_{i}(k)$ reflects the inclination of the flight segment $P_{i,k}^{\prime }P_{i,k+1}^{\prime }$. It can be computed based on the altitude difference and horizontal projection length, as expressed in Equation (22):

(22) $$∆\theta_{i}\left(k\right)=arctan(\frac{∆z_{k}}{P_{i,k}^{\prime }P_{i,k+1}^{\prime }})$$

Based on this, an indicator function $a_{i}(k)$ is defined to determine whether ${UAV}_{i}$ exceeds the thresholds ${\;\phi }_{max}$ or $\theta_{max}$ at ${waypoint}_{k}$:

(23) $$a_{i}\left(k\right)=\left\{\begin{array}{l} 1\mathrm{if}\left|\emptyset_{i}\left(k\right)\right|>\phi_{max}or\left|∆\theta_{i}\left(k\right)\right|>\theta_{max}\\ 0\;\;\;\;\;\;\;\;\;\;\;\;\;\;\;\;\;\;\;\;\;\;\;\;\mathrm{otherwise} \end{array}\right.$$

In Equation (23), if the horizontal turning angle or climb/descent angle at a waypoint exceeds the preset threshold, the point is considered to violate the constraints. By aggregating the results across all waypoints, the single-UAV angle constraint indicator can be obtained as Equation (24):

(24) $$A_{i}=\left\{\begin{array}{l} 1,if\exists k\mathrm{makes}a_{i}\left(k\right)=1\\ 0,\;\mathrm{otherwise} \end{array}\right.$$

That is, if any waypoint of ${UAV}_{i}$ violates the turning angle constraints, the overall indicator $A_{i}$ is set to 1; otherwise, it is 0. Based on this definition, the angle constraint of all UAVs can be incorporated into the overall cost function. The path smoothness cost is expressed as Equation (25):

(25) $$f_{5}=\sum_{i=1}^{N}A_{i}$$

This modeling approach not only strictly enforces individual UAV turning and climb/descent constraints but also effectively prevents excessively steep or sharp-turning trajectories when aggregated across the UAV fleet. Compared to single-point constraints, this overall cost function better balances group flight coordination and stability during optimization, thereby improving trajectory smoothness and global safety, and providing a more reasonable evaluation for subsequent path planning.

#### 5.1.5. Time Synchronization Constraint

For ${UAV}_{i}$, the total flight time can be calculated as the ratio of the path length to the average speed, as shown in Equation (26):

(26) $$t_{i}=\frac{L_{i}}{v_{i}}$$

To ensure coordinated timing among multiple UAVs, a time synchronization cost function is introduced:

(27) $$f_{6}=\sum_{i=1}^{N}p_{4}\cdot \left|t_{i}-t_{c}\right|$$

In Equation (27), $p_{4}$ is the weight for time synchronization, and $t_{c}$ represents the preset mission coordination time. This constraint ensures that all UAVs maintain temporal coordination after path planning.

#### 5.1.6. Spatial Coordination and Collision Cost

**Figure 14:**
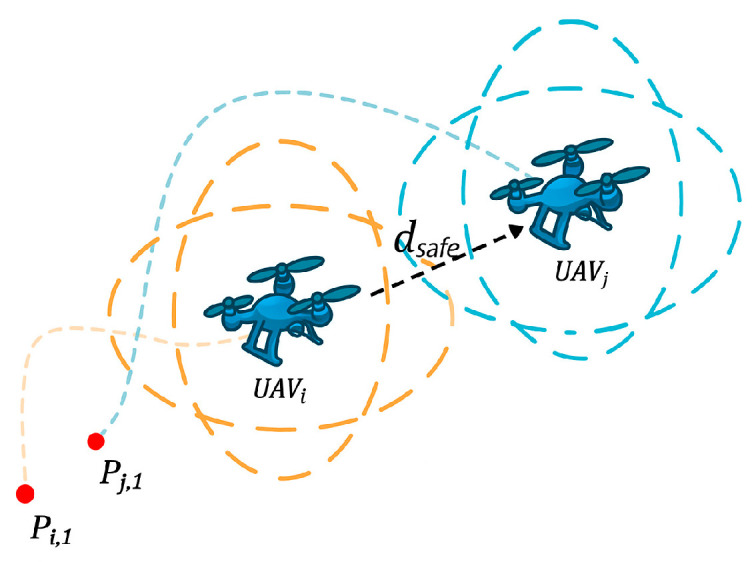
Safety distance diagram.

As shown in Figure 14, if the distance between any two UAVs within the same time interval is less than the safety threshold $d_{safe}$, it is considered a potential collision event, denoted by $C_{ij}$. Based on this, the spatial coordination cost is defined as Equation (28):

(28) $$f_{7}=p_{5}\cdot \sum_{1\leq i\leq j\leq N}C_{ij}$$

Here, $p_{5}$ is the spatial coordination or collision penalty factor. This metric is used to quantify the degree to which multiple UAVs maintain safe distances in space.

#### 5.1.7. Minimum Path Segment Constraint

Let the length of a certain path segment of ${UAV}_{i}\;$ be $l_{i,j}=\left\|p_{i,k+1}-p_{i,k}\right\|$. If $l_{i,j}$ < $L_{min}$, where $L_{min}$ is the predefined minimum allowable segment length, the path segment does not satisfy the feasibility requirement. Its penalty term can be expressed as Equation (29):

(29) $$t_{i}\left(k\right)=\left\{\begin{array}{c} 1,l_{i,j}<L_{min}\\ 0,\mathrm{otherwise} \end{array}\right.$$

(30) $$f_{8}=p_{5}\cdot \sum_{i=1}^{N}\sum_{k=1}^{m_{i}-1}t_{i}\left(k\right)$$

This constraint prevents excessively short path segments, ensuring the rationality and stability of the planned path. In summary, the path planning evaluation function for each UAV can be expressed as in Equation (31):

(31) $$F_{all}=\lambda_{1}f_{1}+\lambda_{2}f_{2}+\lambda_{3}f_{3}+\lambda_{4}f_{4}+\lambda_{5}f_{5}+\lambda_{6}f_{6}+\lambda_{7}f_{7}+\lambda_{8}f_{8}$$

Here, $\lambda_{1}$, $\lambda_{2}$, $\lambda_{3}$, $\lambda_{4}$, $\lambda_{5}$, $\lambda_{6}$, $\lambda_{7}$, and $\lambda_{8}$ are the weighting coefficients for each cost component and can be set according to mission requirements and the dimensional differences of each component. Thus, the multi-UAV 3D path planning problem is transformed into an optimization problem with the comprehensive cost function *F* as the objective.

### 5.2. Multi-UAV Path Simulation Experiment

In this experiment, two types of terrain maps and three threat layouts were designed, resulting in six application scenarios to comprehensively evaluate the performance of the algorithms. The UAV formation consists of four vehicles, with each trajectory represented by 15 control points. These scenarios serve as test platforms to compare the path-planning effectiveness of the improved IWMA against seven representative algorithms.

**Figure 15:**
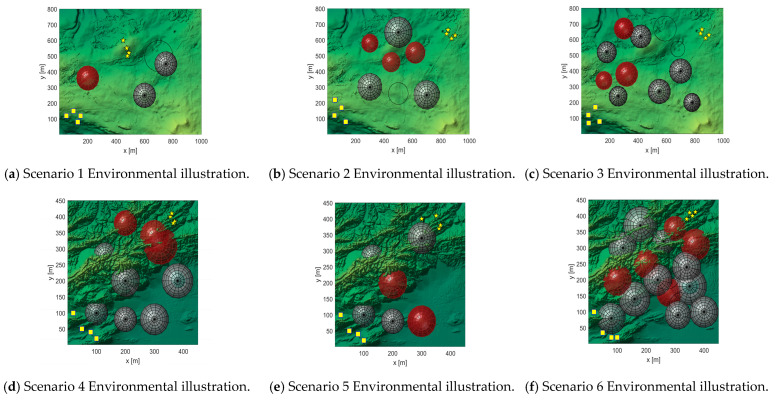
Environmental illustration.

The experimental parameters are configured as follows: the population size is set to 100, and the maximum number of iterations is 200. The fitness function is composed of multiple cost components with weights of 1, 1, 20, 10, 5, 3, 8, and 5. To enhance trajectory safety and formation coordination, greater emphasis is placed on safety-related components such as threat avoidance, collision prevention, and time synchronization. This weighting strategy improves both the robustness of the planned paths and the coordination of the UAV formation. For each application scenario, all eight algorithms are executed independently 10 times to ensure the statistical reliability of the results. The overall layout of the experimental scenarios is illustrated in Figure 15.

In Figure 15, the red and black circles represent radar threats and ground obstacles, respectively, which the UAVs must avoid. The yellow squares indicate the start positions, while the yellow pentagrams denote the goal positions of the UAVs.

Across the six experimental scenarios, the proposed IWMA algorithm consistently outperforms all benchmark methods. In Scenario 1, IWMA achieves the lowest mean fitness of 2277.09 and the best optimum of 2250.84, surpassing WMA (2353.19), GWO (2418.44), and IVY (2478.61), while PSO and DBO exceed 2800. Its convergence rapidly progresses toward high-quality regions, in contrast to the slower or oscillatory behavior of the competitors. In Scenario 2, despite increased task difficulty, IWMA maintains the lowest mean (2471.19) and optimum (2463.70), with a faster, smoother convergence than rivals. Scenario 3 further demonstrates IWMA’s robustness under high complexity, achieving a mean of 2756.66 and an optimum of 2721.93, while most baselines stagnate or oscillate. In the lower-cost Scenarios 4 and 5, IWMA shows a pronounced advantage, reaching means of 627.86 and 629.75 and optima of 610.21, consistently outperforming WMA, GWO, DBO, PSO, IVY, and SHADE with rapid, stable convergence. Finally, in the most complex Scenario 6, IWMA secures the lowest mean (750.54) and optimum (713.33), descending quickly in early iterations and maintaining near-stability, unlike its competitors. Overall, IWMA demonstrates superior speed, stability, and accuracy, effectively balancing exploration and exploitation, enabling rapid convergence in simple tasks and sustained high-quality performance in complex, constrained environments, highlighting its potential for UAV trajectory planning and other demanding optimization problems.

According to the statistical results in Table 12, IWMA consistently outperforms the seven competing algorithms across all six scenarios in terms of best, worst, and mean fitness values. In Scenario 1, IWMA achieves an average fitness of 2277.09, corresponding to improvements of 3.2% over WMA, 9.2% over IVY, and up to 19.0% over PSO. In Scenario 2, the mean fitness of IWMA decreases to 2471.19, surpassing PSO by 26.2%, SHADE by 18.3%, and WMA by 3.9%.

The superiority of IWMA becomes more evident in Scenario 3, where it obtains a mean fitness of 2756.66, markedly outperforming WMA (22.4% reduction), PSO (28.4% reduction), and DBO (27.6% reduction). In Scenario 4, IWMA secures an average fitness of 627.86, delivering 8.3% improvement over WMA, 49.0% over IVY, and 49.1% over GTO, thereby demonstrating excellent robustness in relatively low-dimensional yet highly constrained cases.

In Scenario 5, IWMA further achieves a mean fitness of 629.75, representing enhancements of 17.2% compared to WMA, 42.6% compared to IVY, and 29.6% compared to PSO. Finally, in Scenario 6, IWMA attains a mean of 750.54, yielding substantial improvements of 32.1% over WMA, 59.0% over GTO, and 50.3% over SHADE, confirming its adaptability and efficiency in highly complex multi-constraint environments.

**Figure 16:**
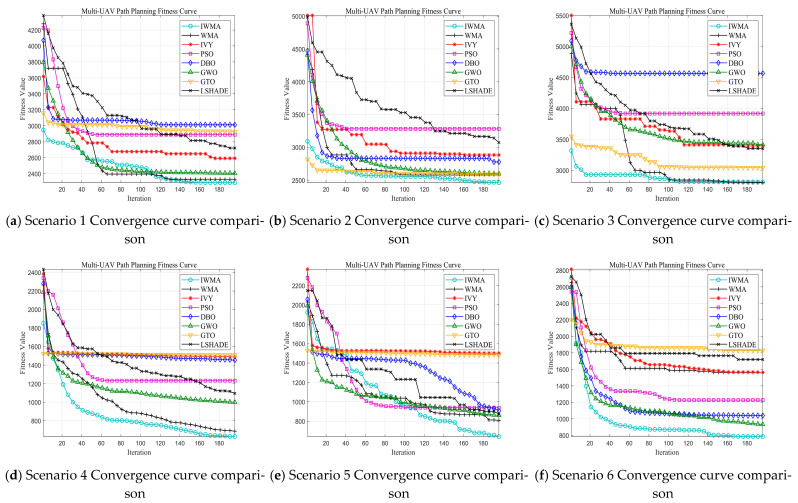
Convergence curve comparison.

Furthermore, the convergence profiles in Figure 16 indicate that IWMA not only converges more rapidly but also exhibits greater stability compared with WMA, IVY, PSO, DBO, GWO, GTO, and SHADE, which frequently suffer from premature convergence or stagnation in local optima. This superior convergence behavior is consistent across different benchmark functions and mission scenarios, demonstrating IWMA’s robust search capability in both simple and complex optimization landscapes. Collectively, these results provide strong evidence that IWMA effectively balances global exploration and local exploitation, enabling it to consistently deliver high-quality and reliable solutions across diverse and challenging UAV path planning scenarios, while maintaining stability even under stringent environmental constraints.

**Figure 17:**
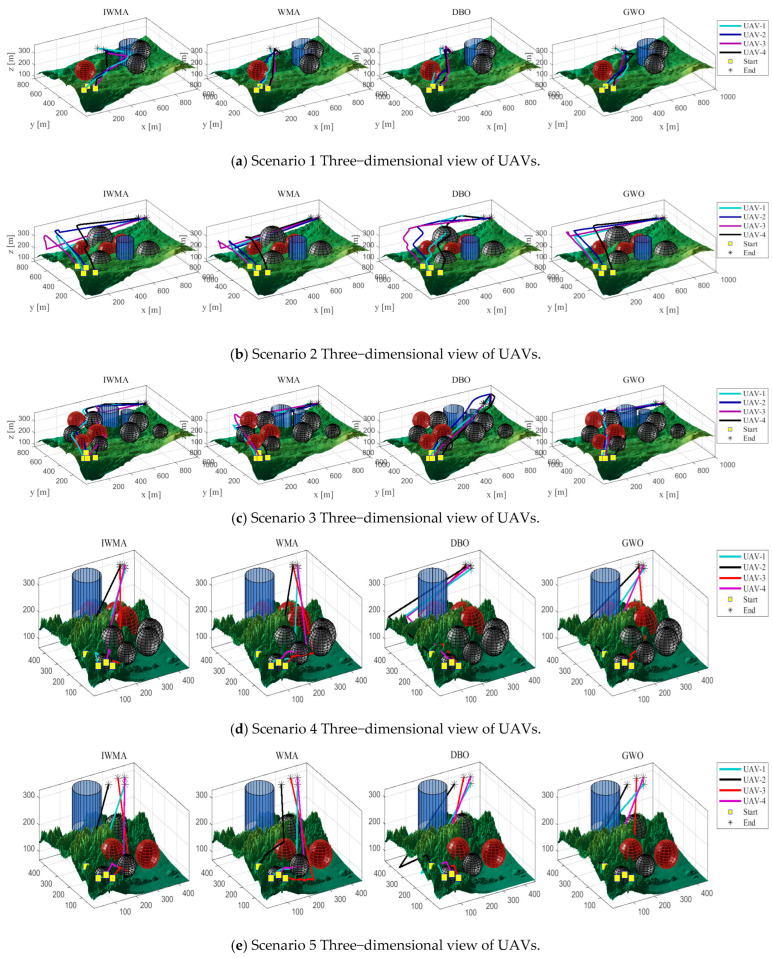

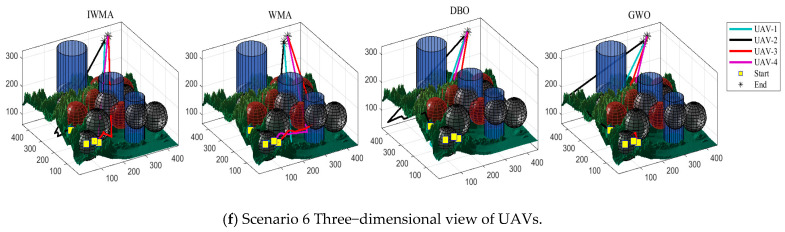
Three−dimensional view of UAVs.

**Figure 18:**
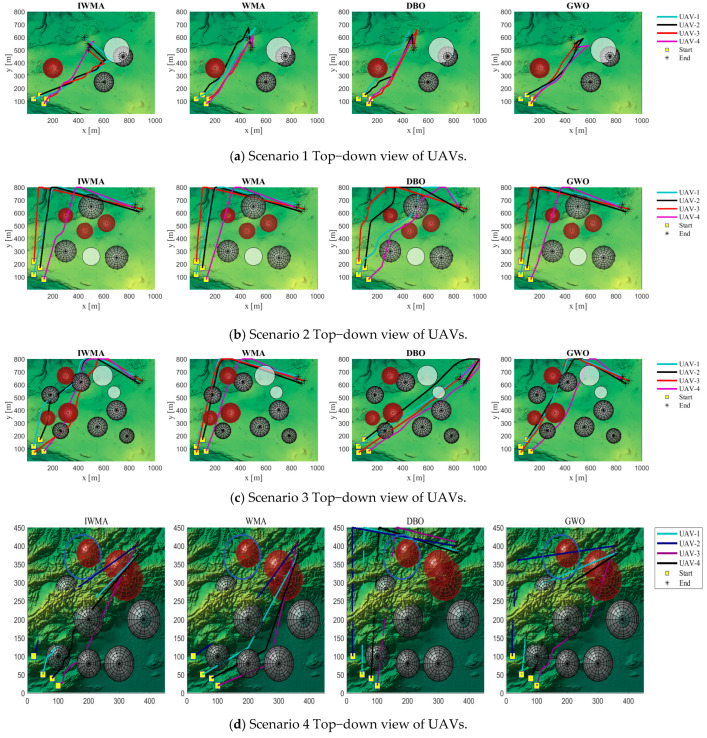

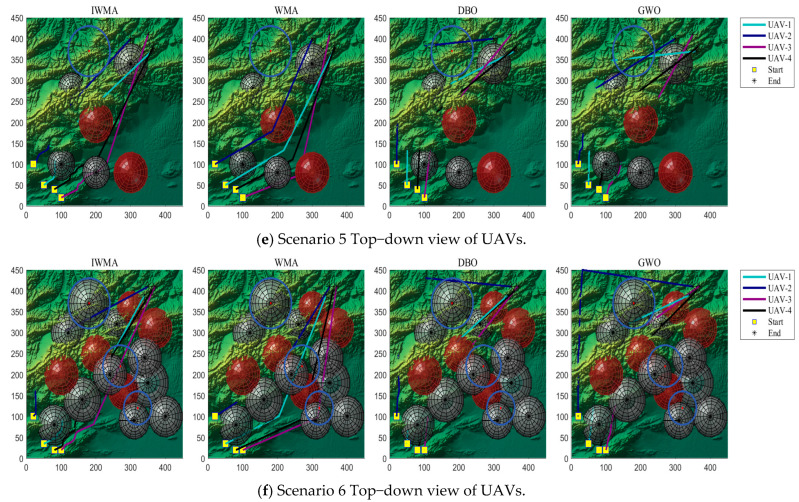
Top−down view of UAVs.

**Figure 19:**
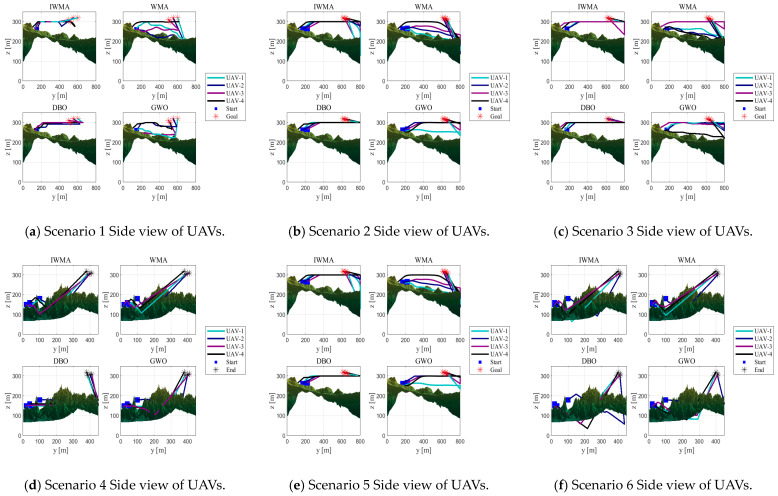
Side view of UAVs.

To visualize and evaluate the performance of the proposed method, four representative algorithms—IWMA, WMA, DBO, and GWO—were selected for comparison of three-dimensional trajectories across six typical UAV mission scenarios, covering a range of environmental complexities and obstacle distributions. IWMA and WMA share the same underlying framework, allowing a direct and quantitative assessment of IWMA’s improvements in path optimization and collision avoidance, including reduced path length, smoother turning, and higher obstacle-avoidance success rates. This comparison also facilitates a detailed evaluation of IWMA’s global search capability, local optimization efficiency, and robustness under varying task complexities and environmental constraints. Meanwhile, DBO and GWO, as widely used Swarm Intelligence Algorithms, serve as benchmarks to illustrate typical performance differences in UAV trajectory planning. Figure 17, Figure 18 and Figure 19 depict the three-dimensional trajectories, top views, and side views, providing comprehensive comparisons of path length, turning frequency, trajectory smoothness, energy consumption, obstacle-avoidance performance, and overall trajectory feasibility. Overall, IWMA consistently produces trajectories that are shorter, smoother, and safer, while demonstrating strong adaptability to dynamic and constrained environments. In contrast, the other algorithms exhibit limitations such as excessive detours, redundant or overlapping segments, abrupt turns, and insufficient safety margins, potentially reducing operational efficiency and compromising mission execution. This visualization thus offers an intuitive and quantitative means of assessing algorithm performance across multiple UAV trajectory planning scenarios, highlighting the practical advantages and robustness of the proposed IWMA approach.

A closer examination of Figure 17, Figure 18 and Figure 19 shows that IWMA performs particularly well in high-threat scenarios 3 and 6. Its trajectories effectively avoid dangerous regions while remaining compact and smooth, demonstrating strong global optimization capability and robustness. In comparison, WMA exhibits limited obstacle-avoidance ability, with local paths often skirting threat boundaries and thereby reducing safety. DBO generates pronounced detours, resulting in redundant flight paths and increased travel distance. GWO, although capable of bypassing threats, produces trajectories with overly frequent and abrupt turns, leading to reduced smoothness and greater flight instability. The convergence curves provide further evidence. IWMA rapidly approaches near-optimal solutions in most scenarios and maintains stable refinement in later iterations, whereas the competing algorithms converge more slowly or are prone to premature stagnation. These results collectively confirm the superior performance and reliability of IWMA in complex three-dimensional environments, The green areas in the figures represent vegetation in the mountainous terrain.

## 6. Conclusions

In this study, we propose an Improved Whale Migration Algorithm (IWMA) that incorporates three tailored strategies: Circle chaotic mapping to enhance population diversity at initialization, a three-layer cooperative search structure to balance global exploration and local exploitation, and a t-distribution-based adaptive control mechanism to dynamically adjust the search process. Theoretical analysis confirms that these enhancements preserve the original WMA’s time complexity, introducing no additional computational burden.

Extensive evaluations on the CEC2017 benchmark suite demonstrate the superiority of IWMA. Across 29 test functions in 30 and 50 dimensions, IWMA consistently achieves better results than seven state-of-the-art algorithms (WMA, IVY, PSO, DBO, GWO, GTO, and LSHADE) in terms of best, mean, and standard deviation indicators. Convergence curves, boxplots, and ablation studies further validate the individual and combined effectiveness of the three strategies, highlighting their complementary roles. Moreover, comparisons with other migration-based optimizers (ALA, GGO, WaoA, and SOA) confirm IWMA’s incremental improvements and reinforce the rationale for choosing WMA as the baseline.

Finally, in multi-UAV three-dimensional path planning tasks across six complex environments, IWMA produces safer, smoother, and more energy-efficient trajectories than the competing algorithms. These results demonstrate its strong practicality and robustness. Looking forward, future work will explore its extension to dynamic environments, large-scale UAV swarms, and integration with domain-specific path planning frameworks, further expanding its potential in real-world engineering applications.

## Figures and Tables

**Table 1:** Experimental parameter settings.

Algorithm Parameter	Algorithm Parameter
IWMA	$N_{L}=PopSize/3$
WMA	$N_{L}=PopSize/2$
IVY	*r* = 0.8, β = 1.5
PSO	$c_{1}$ = 3.49445, $c_{2}$ = 3.49445, w = 0.5, $V_{max}$ = 5, $V_{min}$ = 5
DBO	$P_{-}percent$ = 0.2, α = 0.3, β = 0.1, r = 0.9
GWO	A = 1.5,
GTO	α = 0.5, β = 1.5, γ = 0.5
LSHADE	H = 6, P = 0.1, F_initial = 0.5, CR_initial = 0.5

**Table 2:** Optimization results on 30−D F1–F30 benchmarks.

F		IWMA	WMA	IVY	PSO	DBO	GWO	GTO	LSHADE
	Best	**1.02 × 10^2^**	1.57 × 10^2^	1.23 × 10^2^	1.21 × 10^10^	2.14 × 10^4^	1.25 × 10^8^	4.03 × 10^9^	6.28 × 10^2^
F1	Avg	**4.01 × 10^3^**	6.59 × 10^3^	1.41 × 10^8^	2.68 × 10^10^	4.23 × 10^7^	2.61 × 10^9^	6.69 × 10^9^	1.56 × 10^8^
	Std	**4.58 × 10^2^**	7.55 × 10^3^	4.22 × 10^8^	8.34 × 10^9^	3.51 × 10^7^	2.29 × 10^9^	1.54 × 10^9^	8.05 × 10^8^
	Best	**2.86 × 10^3^**	3.29 × 10^3^	3.93 × 10^4^	7.08 × 10^4^	4.91 × 10^4^	2.46 × 10^4^	6.18 × 10^4^	4.48 × 10^4^
F3	Avg	**1.08 × 10^4^**	1.73 × 10^4^	5.96 × 10^4^	1.72 × 10^5^	7.38 × 10^4^	5.15 × 10^4^	6.90 × 10^4^	1.75 × 10^5^
	Std	**3.98 × 10^3^**	1.07 × 10^4^	9.19 × 10^3^	6.04 × 10^4^	1.22 × 10^4^	1.36 × 10^4^	3.32 × 10^3^	9.47 × 10^4^
	Best	**4.04 × 10^2^**	4.25 × 10^2^	4.68 × 10^2^	1.68 × 10^3^	5.07 × 10^2^	5.08 × 10^2^	8.49 × 10^2^	4.26 × 10^2^
F4	Avg	4.87 × 10^2^	4.85 × 10^2^	5.15 × 10^2^	4.978 × 10^3^	5.88 × 10^2^	6.29 × 10^2^	1.26 × 10^3^	**4.52 × 10^2^**
	Std	**1.94 × 10^1^**	2.41 × 10^1^	3.99 × 10^1^	2.99 × 10^3^	7.29 × 10^1^	1.18 × 10^2^	3.05 × 10^2^	3.32 × 10^1^
	Best	**5.49 × 10^2^**	5.71 × 10^2^	6.20 × 10^2^	6.45 × 10^2^	6.46 × 10^2^	5.65 × 10^2^	7.22 × 10^2^	5.51 × 10^2^
F5	Avg	**5.86 × 10^2^**	6.28 × 10^2^	7.23 × 10^2^	7.29 × 10^2^	7.40 × 10^2^	6.06 × 10^2^	7.86 × 10^2^	6.42 × 10^2^
	Std	**2.59 × 10^1^**	4.18 × 10^1^	5.01 × 10^1^	4.45 × 10^1^	5.79 × 10^1^	2.68 × 10^1^	2.86 × 10^1^	6.62 × 10^1^
	Best	**6.00 × 10^2^**	6.09 × 10^2^	6.00 × 10^2^	6.25 × 10^2^	6.34 × 10^2^	6.04 × 10^2^	6.66 × 10^2^	6.01 × 10^2^
F6	Avg	**6.01 × 10^2^**	6.19 × 10^2^	6.23 × 10^2^	6.41 × 10^2^	6.51 × 10^2^	6.11 × 10^2^	6.73 × 10^2^	6.02 × 10^2^
	Std	**1.86 × 10^0^**	6.86 × 10^0^	1.94 × 10^1^	9.12 × 10^0^	1.34 × 10^1^	3.98 × 10^0^	4.14 × 10^0^	1.92 × 10^0^
	Best	**7.84 × 10^2^**	8.85 × 10^2^	9.41 × 10^2^	8.06 × 10^2^	8.31 × 10^2^	8.32 × 10^2^	1.16 × 10^3^	7.93 × 10^2^
F7	Avg	**8.31 × 10^2^**	8.85 × 10^2^	1.21 × 10^3^	1.08 × 10^3^	1.01 × 10^3^	8.86 × 10^2^	1.32 × 10^3^	8.79 × 10^2^
	Std	**3.75 × 10^1^**	4.66 × 10^1^	1.13 × 10^2^	1.27 × 10^2^	8.74 × 10^1^	3.81 × 10^1^	6.14 × 10^1^	5.23 × 10^1^
	Best	**8.51 × 10^2^**	8.44 × 10^2^	8.75 × 10^2^	9.09 × 10^2^	9.18 × 10^2^	8.63 × 10^2^	9.71 × 10^2^	8.53 × 10^2^
F8	Avg	**8.74 × 10^2^**	8.93 × 10^2^	9.54 × 10^2^	9.81 × 10^2^	1.03 × 10^3^	9.11 × 10^2^	9.96 × 10^2^	9.47 × 10^2^
	Std	**1.22 × 10^1^**	3.08 × 10^1^	3.12 × 10^1^	4.39 × 10^1^	5.06 × 10^1^	4.03 × 10^1^	1.44 × 10^1^	5.65 × 10^1^
	Best	**9.01 × 10^2^**	1.12 × 10^3^	4.34 × 10^3^	2.37 × 10^3^	1.79 × 10^3^	1.21 × 10^3^	6.89 × 10^3^	9.51 × 10^2^
F9	Avg	1.36 × 10^3^	3.22 × 10^3^	5.25 × 10^3^	7.78 × 10^3^	5.39 × 10^3^	2.44 × 10^3^	8.01 × 10^3^	**1.24 × 10^3^**
	Std	6.52 × 10^2^	1.55 × 10^3^	**3.94 × 10^2^**	4.37 × 10^3^	2.00 × 10^3^	8.62 × 10^2^	7.01 × 10^2^	9.47 × 10^2^
	Best	4.25 × 10^3^	6.00 × 10^3^	**4.19 × 10^3^**	4.69 × 10^3^	4.57 × 10^3^	3.47 × 10^3^	5.21 × 10^3^	5.63 × 10^3^
F10	Avg	6.45 × 10^3^	7.11 × 10^3^	5.38 × 10^3^	5.85 × 10^3^	5.80 × 10^3^	**5.02 × 10^3^**	6.95 × 10^3^	7.64 × 10^3^
	Std	1.15 × 10^3^	**4.78 × 10^2^**	6.56 × 10^2^	6.39 × 10^2^	8.80 × 10^2^	1.15 × 10^3^	1.06 × 10^3^	9.28 × 10^2^
	Best	**1.12 × 10^3^**	1.17 × 10^3^	1.22 × 10^3^	1.42 × 10^3^	1.34 × 10^3^	1.28 × 10^3^	1.89 × 10^3^	1.16 × 10^3^
F11	Avg	**1.21 × 10^3^**	1.28 × 10^3^	1.68 × 10^3^	4.46 × 10^3^	1.59 × 10^3^	2.10 × 10^3^	2.52 × 10^3^	1.27 × 10^3^
	Std	**5.04 × 10^1^**	6.78 × 10^1^	6.72 × 10^2^	3.44 × 10^3^	1.71 × 10^2^	9.48 × 10^2^	3.47 × 10^2^	1.06 × 10^2^
	Best	5.66 × 10^7^	**1.87 × 10^4^**	4.02 × 10^5^	6.21 × 10^7^	1.58 × 10^5^	1.02 × 10^7^	1.72 × 10^8^	2.88 × 10^5^
F12	Avg	5.82 × 10^5^	**2.88 × 10^5^**	7.80 × 10^7^	2.58 × 10^9^	7.09 × 10^7^	1.01 × 10^8^	7.46 × 10^8^	4.35 × 10^7^
	Std	**3.95 × 10^5^**	5.88 × 10^5^	4.15 × 10^8^	1.56 × 10^9^	8.35 × 10^7^	1.09 × 10^8^	3.74 × 10^8^	8.24 × 10^7^
	Best	**1.60 × 10^3^**	3.52 × 10^3^	1.15 × 10^4^	2.14 × 10^5^	3.27 × 10^4^	2.40 × 10^4^	5.51 × 10^7^	2.93 × 10^3^
F13	Avg	**1.87 × 10^4^**	1.94 × 10^4^	7.90 × 10^6^	2.13 × 10^9^	3.32 × 10^6^	9.65 × 10^6^	2.28 × 10^8^	1.31 × 10^6^
	Std	**2.03 × 10^4^**	2.11 × 10^4^	3.92 × 10^7^	2.10 × 10^9^	9.85 × 10^6^	4.93 × 10^7^	1.76 × 10^8^	4.68 × 10^6^
	Best	**1.53 × 10^4^**	2.05 × 10^3^	1.24 × 10^4^	6.48 × 10^3^	8.96 × 10^3^	7.10 × 10^3^	1.76 × 10^5^	1.58 × 10^3^
F14	Avg	**5.36 × 10^3^**	1.66 × 10^4^	1.67 × 10^6^	1.11 × 10^6^	2.26 × 10^5^	5.76 × 10^5^	1.29 × 10^6^	2.94 × 10^4^
	Std	**3.68 × 10^3^**	1.21 × 10^4^	1.71 × 10^6^	2.14 × 10^6^	4.24 × 10^5^	6.15 × 10^5^	6.07 × 10^5^	4.71 × 10^4^
	Best	**1.92 × 10^3^**	2.05 × 10^3^	2.62 × 10^3^	2.23 × 10^4^	6.25 × 10^3^	1.31 × 10^4^	7.07 × 10^4^	4.25 × 10^3^
F15	Avg	**7.43 × 10^3^**	1.34 × 10^4^	1.01 × 10^7^	1.18 × 10^8^	5.95 × 10^4^	5.489 × 10^6^	2.72 × 10^5^	1.01 × 10^5^
	Std	**6.47 × 10^3^**	1.15 × 10^4^	3.86 × 10^7^	3.61 × 10^8^	4.59 × 10^4^	1.23 × 10^7^	1.54 × 10^5^	1.99 × 10^5^
	Best	**1.79 × 10^3^**	1.88 × 10^3^	2.39 × 10^3^	2.64 × 10^3^	2.68 × 10^3^	2.18 × 10^3^	3.39 × 10^3^	2.45 × 10^3^
F16	Avg	**2.38 × 10^3^**	2.52 × 10^3^	3.03 × 10^3^	3.61 × 10^3^	3.33 × 10^3^	2.54 × 10^3^	4.03 × 10^3^	3.22 × 10^3^
	Std	3.38 × 10^2^	3.79 × 10^2^	2.98 × 10^2^	5.25 × 10^2^	3.76 × 10^2^	**2.47 × 10^2^**	3.87 × 10^2^	3.77 × 10^2^
	Best	**1.75 × 10^3^**	1.82 × 10^3^	2.20 × 10^3^	2.20 × 10^3^	1.96 × 10^3^	1.81 × 10^3^	2.18 × 10^3^	1.86 × 10^3^
F17	Avg	**1.95 × 10^3^**	2.23 × 10^3^	2.74 × 10^3^	2.74 × 10^3^	2.55 × 10^3^	2.07 × 10^3^	2.76 × 10^3^	2.35 × 10^3^
	Std	**1.51 × 10^2^**	2.37 × 10^2^	3.35 × 10^2^	2.97 × 10^2^	2.73 × 10^2^	1.71 × 10^2^	2.84 × 10^2^	2.65 × 10^2^
	Best	**1.12 × 10^4^**	1.13 × 10^4^	1.28 × 10^5^	2.51 × 10^5^	8.03 × 10^4^	9.64 × 10^4^	5.41 × 10^4^	5.44 × 10^4^
F18	Avg	**1.22 × 10^5^**	1.75 × 10^5^	2.10 × 10^6^	1.42 × 10^7^	2.16 × 10^6^	1.29 × 10^6^	4.26 × 10^6^	1.74 × 10^6^
	Std	**1.19 × 10^5^**	1.69 × 10^5^	2.62 × 10^6^	2.46 × 10^7^	3.34 × 10^6^	2.18 × 10^6^	2.42 × 10^6^	2.29 × 10^6^
	Best	**2.08 × 10^3^**	2.10 × 10^3^	2.32 × 10^3^	1.29 × 10^5^	1.46 × 10^4^	5.32 × 10^3^	5.02 × 10^5^	2.06 × 10^3^
F19	Avg	**6.30 × 10^3^**	1.86 × 10^4^	3.87 × 10^6^	1.33 × 10^8^	1.49 × 10^6^	4.74 × 10^6^	2.29 × 10^6^	6.69 × 10^4^
	Std	**4.58 × 10^3^**	1.77 × 10^4^	1.96 × 10^7^	2.55 × 10^8^	3.23 × 10^6^	9.99 × 10^6^	1.33 × 10^6^	1.24 × 10^5^
	Best	**2.15 × 10^3^**	2.16 × 10^3^	2.37 × 10^3^	2.39 × 10^3^	2.19 × 10^3^	2.25 × 10^3^	2.37 × 10^3^	2.08 × 10^3^
F20	Avg	**2.31 × 10^3^**	2.35 × 10^3^	2.74 × 10^3^	2.67 × 10^3^	2.72 × 10^3^	2.42 × 10^3^	2.85 × 10^3^	2.59 × 10^3^
	Std	1.35 × 10^2^	1.39 × 10^2^	1.75 × 10^2^	1.38 × 10^2^	2.33 × 10^2^	**1.32 × 10^2^**	2.05 × 10^2^	2.69 × 10^2^
	Best	**2.34 × 10^3^**	2.35 × 10^3^	2.35 × 10^3^	2.40 × 10^3^	2.48 × 10^3^	2.35 × 10^3^	2.54 × 10^3^	2.35 × 10^3^
F21	Avg	**2.37 × 10^3^**	2.39 × 10^3^	2.41 × 10^3^	2.52 × 10^3^	2.56 × 10^3^	2.41 × 10^3^	2.60 × 10^3^	2.46 × 10^3^
	Std	**1.91 × 10^1^**	3.56 × 10^1^	3.27 × 10^1^	5.15 × 10^1^	6.25 × 10^1^	4.49 × 10^1^	4.68 × 10^1^	6.25 × 10^1^
	Best	**2.30 × 10^3^**	2.30 × 10^3^	2.30 × 10^3^	5.33 × 10^3^	2.32 × 10^3^	2.51 × 10^3^	3.74 × 10^3^	2.70 × 10^3^
F22	Avg	**2.30 × 10^3^**	5.18 × 10^3^	4.69 × 10^3^	7.56 × 10^4^	4.88 × 10^3^	5.54 × 10^3^	7.57 × 10^3^	7.83 × 10^3^
	Std	**2.95 × 10^0^**	2.88 × 10^3^	2.38 × 10^3^	8.58 × 10^2^	2.35 × 10^3^	2.19 × 10^3^	1.18 × 10^3^	2.51 × 10^3^
	Best	**2.69 × 10^3^**	2.71 × 10^3^	2.74 × 10^3^	2.90 × 10^3^	2.81 × 10^3^	2.72 × 10^3^	3.17 × 10^3^	2.72 × 10^3^
F23	Avg	**2.73 × 10^3^**	2.77 × 10^3^	2.83 × 10^3^	3.03 × 10^3^	2.98 × 10^3^	2.79 × 10^3^	3.37 × 10^3^	2.82 × 10^3^
	Std	**2.25 × 10^1^**	3.83 × 10^1^	9.11 × 10^1^	8.53 × 10^1^	9.13 × 10^1^	6.23 × 10^1^	1.29 × 10^2^	5.59 × 10^1^
	Best	**2.86 × 10^3^**	2.87 × 10^3^	2.87 × 10^3^	3.08 × 10^3^	3.03 × 10^3^	2.87 × 10^3^	3.43 × 10^3^	2.87 × 10^3^
F24	Avg	**2.90 × 10^3^**	2.92 × 10^3^	2.98 × 10^3^	3.21 × 10^3^	3.15 × 10^3^	2.96 × 10^3^	3.64 × 10^3^	2.99 × 10^3^
	Std	**2.89 × 10^1^**	3.57 × 10^1^	1.28 × 10^2^	8.91 × 10^1^	7.46 × 10^1^	6.41 × 10^1^	1.65 × 10^2^	5.74 × 10^1^
	Best	**2.88 × 10^3^**	2.88 × 10^3^	2.88 × 10^3^	3.09 × 10^3^	2.88 × 10^3^	2.92 × 10^3^	3.09 × 10^3^	2.88 × 10^3^
F25	Avg	**2.89 × 10^3^**	2.90 × 10^3^	2.92 × 10^3^	3.73 × 10^3^	2.95 × 10^3^	3.00 × 10^3^	3.14 × 10^3^	2.90 × 10^3^
	Std	1.86 × 10^1^	2.04 × 10^1^	2.01 × 10^1^	7.23 × 10^2^	4.72 × 10^1^	3.93 × 10^1^	3.15 × 10^1^	**1.12 × 10^1^**
	Best	**2.80 × 10^3^**	2.80 × 10^3^	4.38 × 10^3^	4.39 × 10^3^	5.52 × 10^3^	4.26 × 10^3^	4.09 × 10^3^	4.01 × 10^3^
F26	Avg	**4.30 × 10^3^**	5.20 × 10^3^	7.22 × 10^3^	7.68 × 10^3^	6.83 × 10^3^	4.99 × 10^3^	7.59 × 10^3^	5.07 × 10^3^
	Std	**8.05 × 10^2^**	6.55 × 10^2^	8.89 × 10^2^	1.23 × 10^3^	6.47 × 10^2^	3.42 × 10^2^	1.16 × 10^3^	5.17 × 10^2^
	Best	**3.20 × 10^3^**	3.22 × 10^3^	3.25 × 10^3^	3.26 × 10^3^	3.25 × 10^3^	3.23 × 10^3^	3.45 × 10^3^	3.21 × 10^3^
F27	Avg	**3.20 × 10^3^**	3.27 × 10^3^	3.38 × 10^3^	3.43 × 10^3^	3.34 × 10^3^	3.27 × 10^3^	3.85 × 10^3^	3.23 × 10^3^
	Std	**2.13 × 10^1^**	4.24 × 10^1^	1.18 × 10^2^	1.21 × 10^2^	8.02 × 10^1^	2.71 × 10^1^	2.90 × 10^2^	2.85 × 10^1^
	Best	**3.19 × 10^3^**	3.10 × 10^3^	3.22 × 10^3^	3.87 × 10^3^	3.29 × 10^3^	3.33 × 10^3^	3.51 × 10^3^	3.29 × 10^3^
F28	Avg	**3.22 × 10^3^**	3.23 × 10^3^	3.28 × 10^3^	6.25 × 10^3^	3.58 × 10^3^	3.46 × 10^3^	3.71 × 10^3^	3.30 × 10^3^
	Std	1.73 × 10^1^	3.05 × 10^1^	2.93 × 10^1^	1.46 × 10^3^	6.24 × 10^2^	9.19 × 10^1^	9.22 × 10^1^	**2.16 × 10^0^**
	Best	**3.41 × 10^3^**	3.65 × 10^3^	3.84 × 10^3^	3.73 × 10^3^	3.87 × 10^3^	3.48 × 10^3^	4.87 × 10^3^	3.28 × 10^3^
F29	Avg	**3.63 × 10^3^**	4.08 × 10^3^	4.48 × 10^3^	4.77 × 10^3^	4.49 × 10^3^	3.90 × 10^3^	5.38 × 10^3^	4.11 × 10^3^
	Std	**1.86 × 10^2^**	2.99 × 10^2^	2.92 × 10^2^	7.48 × 10^2^	3.65 × 10^2^	2.20 × 10^2^	2.94 × 10^2^	4.89 × 10^2^
	Best	**3.08 × 10^3^**	6.33 × 10^3^	1.55 × 10^4^	3.96 × 10^5^	1.55 × 10^4^	1.20 × 10^6^	4.12 × 10^6^	3.35 × 10^3^
F30	Avg	**1.44 × 10^4^**	1.60 × 10^4^	6.95 × 10^4^	1.17 × 10^8^	3.77 × 10^6^	1.05 × 10^7^	2.29 × 10^7^	4.59 × 10^4^
	Std	**7.33 × 10^3^**	1.21 × 10^4^	4.82 × 10^4^	3.49 × 10^8^	5.51 × 10^6^	9.62 × 10^6^	1.14 × 10^7^	1.27 × 10^5^

**Table 3:** Optimization results on 50−D F1–F30 benchmarks.

F		IWMA	WMA	IVY	PSO	DBO	GWO	GTO	LSHADE
	Best	4.27 × 10^5^	**6.21 × 10^2^**	4.94 × 10^3^	6.77 × 10^10^	3.72 × 10^8^	5.92 × 10^9^	2.21 × 10^10^	6.61 × 10^2^
F1	Avg	2.08 × 10^6^	2.56 × 10^5^	**2.74 × 10^4^**	8.31 × 10^10^	9.15 × 10^8^	1.07 × 10^10^	2.70 × 10^10^	1.04 × 10^10^
	Std	2.55 × 10^6^	7.69 × 10^5^	**3.04 × 10^5^**	1.18 × 10^10^	5.06 × 10^8^	3.72 × 10^9^	4.41 × 10^9^	3.22 × 10^10^
	Best	**6.09 × 10^4^**	8.45 × 10^4^	1.93 × 10^5^	3.10 × 10^5^	1.70 × 10^5^	1.25 × 10^5^	1.45 × 10^5^	1.53 × 10^5^
F3	Avg	**8.98 × 10^4^**	1.42. × 10^5^	2.86 × 10^5^	4.24 × 10^5^	2.29 × 10^5^	1.50 × 10^5^	1.71 × 10^5^	4.01 × 10^5^
	Std	**1.97 × 10^4^**	3.13 × 10^4^	4.64 × 10^4^	7.03 × 10^4^	4.10 × 10^4^	1.28 × 10^4^	1.59 × 10^4^	2.17 × 10^4^
	Best	**4.14 × 10^2^**	4.55 × 10^2^	5.65 × 10^2^	8.27 × 10^3^	6.27 × 10^2^	9.93 × 10^2^	2.43 × 10^3^	4.45 × 10^2^
F4	Avg	5.92 × 10^2^	**5.69 × 10^2^**	6.56 × 10^2^	1.71 × 10^4^	9.47 × 10^2^	1.81 × 10^3^	3.74 × 10^3^	4.01 × 10^5^
	Std	**5.03 × 10^1^**	5.58 × 10^1^	6.56 × 10^1^	5.24 × 10^3^	2.78 × 10^2^	1.12 × 10^3^	1.08 × 10^3^	6.63 × 10^3^
	Best	**6.52 × 10^2^**	6.69 × 10^2^	7.90 × 10^2^	7.90 × 10^2^	8.00 × 10^2^	6.72 × 10^2^	8.96 × 10^2^	7.15 × 10^2^
F5	Avg	**7.00 × 10^2^**	7.36 × 10^2^	8.59 × 10^2^	1.00 × 10^3^	9.78 × 10^2^	7.36 × 10^2^	7.46 × 10^2^	8.35 × 10^2^
	Std	4.25 × 10^1^	7.62 × 10^1^	4.62 × 10^1^	1.03 × 10^2^	1.32 × 10^2^	3.55 × 10^1^	**2.47 × 10^1^**	7.84 × 10^1^
	Best	**6.08 × 10^2^**	6.29 × 10^2^	6.21 × 10^2^	6.31 × 10^2^	6.46 × 10^2^	6.19 × 10^2^	6.76 × 10^2^	6.00 × 10^2^
F6	Avg	6.13 × 10^2^	6.41 × 10^2^	6.48 × 10^2^	6.48 × 10^2^	6.66 × 10^2^	6.23 × 10^2^	6.83 × 10^2^	**6.03 × 10^2^**
	Std	**3.21 × 10^0^**	1.18 × 10^1^	1.32 × 10^1^	7.75 × 10^0^	1.39 × 10^1^	4.00 × 10^0^	4.37 × 10^0^	3.43 × 10^0^
	Best	**9.13 × 10^2^**	1.01 × 10^3^	1.53 × 10^3^	1.51 × 10^3^	1.01 × 10^3^	1.04 × 10^3^	1.75 × 10^3^	9.66 × 10^2^
F7	Avg	**1.00 × 10^3^**	1.23 × 10^3^	1.69 × 10^3^	1.89 × 10^3^	1.29 × 10^3^	1.15 × 10^3^	1.85 × 10^3^	1.07 × 10^3^
	Std	7.94 × 10^1^	1.19 × 10^2^	8.29 × 10^1^	3.47 × 10^2^	1.70 × 10^2^	7.47 × 10^1^	**4.55 × 10^1^**	8.22 × 10^1^
	Best	**9.83 × 10^2^**	9.90 × 10^2^	1.12 × 10^3^	1.24 × 10^3^	1.13 × 10^3^	9.98 × 10^2^	1.26 × 10^3^	9.02 × 10^2^
F8	Avg	**1.02 × 10^3^**	1.04 × 10^3^	1.20 × 10^3^	1.32 × 10^3^	1.30 × 10^3^	1.05 × 10^3^	1.30 × 10^3^	1.10 × 10^3^
	Std	**3.77 × 10^1^**	3.93 × 10^1^	3.52 × 10^1^	7.79 × 10^1^	1.07 × 10^1^	3.12 × 10^1^	2.55 × 10^1^	1.03 × 10^1^
	Best	**1.88 × 10^3^**	7.95 × 10^3^	1.08 × 10^4^	1.13 × 10^4^	9.07 × 10^3^	6.65 × 10^3^	2.48 × 10^4^	1.08 × 10^3^
F9	Avg	6.16 × 10^3^	1.38 × 10^4^	1.28 × 10^4^	2.63 × 10^4^	2.03 × 10^4^	1.27 × 10^4^	2.82 × 10^4^	**4.50 × 10^3^**
	Std	3.93 × 10^3^	3.73 × 10^3^	**1.13 × 10^3^**	9.40 × 10^3^	8.69 × 10^3^	4.81 × 10^3^	2.45 × 10^3^	7.16 × 10^3^
	Best	1.12 × 10^4^	1.24 × 10^4^	6.71 × 10^3^	8.71 × 10^3^	8.46 × 10^3^	**6.31 × 10^3^**	9.78 × 10^3^	9.12 × 10^3^
F10	Avg	1.27 × 10^4^	1.31 × 10^4^	**8.63 × 10^3^**	1.00 × 10^4^	1.09 × 10^4^	9.02 × 10^3^	1.16 × 10^4^	1.33 × 10^4^
	Std	9.65 × 10^2^	**3.55 × 10^2^**	1.35 × 10^3^	9.20 × 10^2^	2.17 × 10^3^	2.92 × 10^3^	1.61 × 10^3^	2.37 × 10^3^
	Best	**1.24 × 10^3^**	1.35 × 10^3^	1.32 × 10^3^	2.60 × 10^3^	1.80 × 10^3^	4.31 × 10^3^	7.33 × 10^3^	2.01 × 10^3^
F11	Avg	**1.34 × 10^3^**	1.40 × 10^3^	2.31 × 10^3^	1.07 × 10^4^	2.90 × 10^3^	6.06 × 10^3^	8.93 × 10^3^	4.43 × 10^3^
	Std	9.23 × 10^1^	**4.20 × 10^1^**	6.79 × 10^2^	8.92 × 10^3^	1.68 × 10^3^	1.60 × 10^3^	1.15 × 10^3^	3.60 × 10^3^
	Best	**5.98 × 10^5^**	1.93 × 10^6^	5.85 × 10^6^	1.53 × 10^10^	8.92 × 10^7^	8.48 × 10^7^	5.02 × 10^9^	3.65 × 10^7^
F12	Avg	5.49 × 10^6^	**4.48 × 10^6^**	1.87 × 10^7^	2.99 × 10^10^	4.56 × 10^8^	1.61 × 10^9^	8.98 × 10^9^	7.95 × 10^8^
	Std	5.05 × 10^6^	**2.20 × 10^6^**	1.08 × 10^7^	1.14 × 10^10^	3.47 × 10^8^	1.67 × 10^9^	2.41 × 10^9^	1.41 × 10^9^
	Best	**1.36 × 10^3^**	4.67 × 10^3^	7.31 × 10^3^	5.35 × 10^9^	5.37 × 10^5^	1.93 × 10^7^	1.27 × 10^9^	2.98 × 10^3^
F13	Avg	**1.19 × 10^3^**	1.82. × 10^4^	6.08 × 10^8^	1.07 × 10^10^	3.86 × 10^7^	1.65 × 10^8^	2.85 × 10^9^	6.54 × 10^7^
	Std	**6.27 × 10^3^**	1.41 × 10^4^	1.90 × 10^9^	3.94 × 10^9^	3.00 × 10^7^	1.61 × 10^8^	1.55 × 10^9^	2.06 × 10^8^
	Best	**1.42 × 10^3^**	1.71 × 10^4^	6.49 × 10^5^	1.01 × 10^6^	1.27 × 10^5^	9.74 × 10^4^	4.95 × 10^6^	1.76 × 10^5^
F14	Avg	**1.06 × 10^5^**	7.50 × 10^4^	3.40 × 10^6^	1.26 × 10^7^	2.98 × 10^6^	2.41 × 10^6^	9.38 × 10^6^	2.12 × 10^6^
	Std	8.21 × 10^4^	**4.96 × 10^4^**	2.77 × 10^6^	1.87 × 10^7^	4.02 × 10^6^	3.65 × 10^6^	3.46 × 10^6^	1.91 × 10^6^
	Best	**3.43 × 10^3^**	1.95 × 10^3^	8.26 × 10^3^	6.00 × 10^4^	1.33 × 10^5^	3.63 × 10^4^	1.27 × 10^7^	2.32 × 10^4^
F15	Avg	**1.21 × 10^4^**	1.54 × 10^4^	8.05 × 10^6^	6.58 × 10^8^	1.78 × 10^6^	7.27 × 10^7^	556 × 10^7^	3.02 × 10^6^
	Std	**5.55 × 10^3^**	9.83 × 10^3^	2.54 × 10^7^	9.00 × 10^8^	4.10 × 10^6^	1.91 × 10^8^	3.60 × 10^7^	7.58 × 10^6^
	Best	**2.08 × 10^3^**	2.55 × 10^3^	3.65 × 10^3^	4.53 × 10^3^	4.07 × 10^3^	2.42 × 10^3^	4.02 × 10^3^	2.78 × 10^3^
F16	Avg	**2.73 × 10^3^**	3.41 × 10^3^	3.99 × 10^3^	5.51 × 10^3^	4.78 × 10^3^	3.19 × 10^3^	4.64 × 10^3^	4.73 × 10^3^
	Std	**3.72 × 10^2^**	6.25 × 10^2^	2.54 × 10^2^	9.00 × 10^2^	4.10 × 10^2^	1.91 × 10^2^	3.60 × 10^2^	7.58 × 10^2^
	Best	**2.28 × 10^3^**	6.32 × 10^3^	2.47 × 10^3^	4.11 × 10^3^	2.94 × 10^3^	2.74 × 10^3^	3.15 × 10^3^	2.80 × 10^3^
F17	Avg	**2.64 × 10^3^**	3.28 × 10^3^	3.59 × 10^3^	4.67 × 10^3^	4.18 × 10^3^	3.03 × 10^3^	4.03 × 10^3^	3.87 × 10^3^
	Std	**2.92 × 10^2^**	3.64 × 10^2^	7.26 × 10^2^	4.06 × 10^2^	6.82 × 10^2^	1.75 × 10^2^	5.12 × 10^2^	5.70 × 10^2^
	Best	**1.27 × 10^5^**	1.31 × 10^5^	9.55 × 10^5^	1.55 × 10^6^	7.12 × 10^5^	1.19 × 10^6^	8.68 × 10^6^	5.14 × 10^5^
F18	Avg	5.47 × 10^5^	**2.92 × 10^5^**	3.87 × 10^6^	3.68 × 10^7^	9.49 × 10^6^	6.32 × 10^6^	1.99 × 10^7^	1.14 × 10^7^
	Std	5.31 × 10^5^	**1.43 × 10^5^**	2.96 × 10^6^	3.54 × 10^7^	7.29 × 10^6^	6.82 × 10^6^	7.95 × 10^6^	8.72 × 10^6^
	Best	**4.72 × 10^3^**	2.16 × 10^3^	1.60 × 10^4^	1.77 × 10^5^	5.70 × 10^3^	1.83 × 10^5^	1.95 × 10^6^	6.12 × 10^3^
F19	Avg	2.21 × 10^4^	**1.66 × 10^4^**	2.86 × 10^6^	3.68 × 10^7^	9.49 × 10^6^	6.32 × 10^6^	1.99 × 10^7^	1.14 × 10^7^
	Std	**1.36 × 10^4^**	1.44 × 10^4^	8.78 × 10^6^	1.35 × 10^9^	4.95 × 10^6^	1.11 × 10^6^	1.25 × 10^7^	1.01 × 10^5^
	Best	**2.35 × 10^3^**	3.23 × 10^3^	2.91 × 10^3^	3.14 × 10^3^	3.17 × 10^3^	2.77 × 10^3^	3.55 × 10^3^	3.02 × 10^3^
F20	Avg	**2.90 × 10^3^**	3.40 × 10^3^	3.57 × 10^3^	3.54 × 10^3^	3.76 × 10^3^	3.23 × 10^3^	3.78 × 10^3^	3.87 × 10^3^
	Std	4.51 × 10^2^	**1.45 × 10^2^**	3.89 × 10^2^	3.17 × 10^2^	3.50 × 10^2^	4.11 × 10^2^	1.63 × 10^2^	4.64 × 10^2^
	Best	**2.41 × 10^3^**	2.48 × 10^3^	2.51 × 10^3^	2.71 × 10^3^	2.74 × 10^3^	2.45 × 10^3^	2.83 × 10^3^	2.58 × 10^3^
F21	Avg	**2.48 × 10^3^**	2.56 × 10^3^	2.57 × 10^3^	2.82 × 10^3^	2.82 × 10^3^	2.56 × 10^3^	2.93 × 10^3^	2.68 × 10^3^
	Std	4.82 × 10^1^	1.00 × 10^2^	**4.45 × 10^1^**	7.21 × 10^1^	6.93 × 10^1^	7.84 × 10^1^	9.03 × 10^1^	7.00 × 10^1^
	Best	**7.49 × 10^3^**	1.41 × 10^4^	8.52 × 10^3^	1.13 × 10^4^	9.31 × 10^3^	8.22 × 10^3^	1.26 × 10^4^	1.27 × 10^4^
F22	Avg	1.23 × 10^4^	1.48 × 10^4^	**1.01 × 10^4^**	1.31 × 10^4^	1.08 × 10^4^	1.03 × 10^4^	1.44 × 10^4^	1.56 × 10^4^
	Std	2.92 × 10^3^	6.44 × 10^2^	1.11 × 10^3^	**1.03 × 10^3^**	1.13 × 10^3^	1.96 × 10^3^	1.33 × 10^3^	1.64 × 10^3^
	Best	**2.84 × 10^3^**	2.95 × 10^3^	2.89 × 10^3^	3.48 × 10^3^	3.33 × 10^3^	2.90 × 10^3^	3.97 × 10^3^	2.85 × 10^3^
F23	Avg	**2.94 × 10^3^**	3.08 × 10^3^	3.04 × 10^3^	3.64 × 10^3^	3.51 × 10^3^	3.03 × 10^3^	4.28 × 10^3^	3.02 × 10^3^
	Std	**7.56 × 10^1^**	7.58 × 10^1^	1.02 × 10^2^	1.34 × 10^2^	1.20 × 10^2^	9.88 × 10^1^	2.35 × 10^2^	1.32 × 10^2^
	Best	**3.02 × 10^3^**	3.05 × 10^3^	3.12 × 10^3^	5.27 × 10^3^	3.10 × 10^3^	3.44 × 10^3^	4.14 × 10^3^	3.03 × 10^3^
F24	Avg	**3.06 × 10^3^**	3.16 × 10^3^	3.21 × 10^3^	3.79 × 10^3^	3.61 × 10^3^	3.23 × 10^3^	4.70 × 10^3^	3.36 × 10^3^
	Std	**3.42 × 10^1^**	5.66 × 10^1^	7.48 × 10^1^	1.32 × 10^2^	1.08 × 10^2^	1.12 × 10^2^	3.10 × 10^2^	9.56 × 10^1^
	Best	**3.03 × 10^3^**	3.06 × 10^3^	3.12 × 10^3^	5.27 × 10^3^	3.10 × 10^3^	3.44 × 10^3^	4.14 × 10^3^	2.93 × 10^3^
F25	Avg	3.13 × 10^3^	**3.07 × 10^3^**	3.15 × 10^3^	9.34 × 10^3^	3.86 × 10^3^	3.72 × 10^3^	5.21 × 10^3^	3.10 × 10^3^
	Std	3.88 × 10^1^	**2.82 × 10^1^**	4.63 × 10^1^	2.40 × 10^3^	1.89 × 10^3^	2.00 × 10^2^	5.17 × 10^2^	3.52 × 10^2^
	Best	**2.94 × 10^3^**	6.23 × 10^3^	8.52 × 10^3^	1.28 × 10^4^	7.24 × 10^3^	6.55 × 10^3^	1.02 × 10^4^	5.56 × 10^3^
F26	Avg	**5.86 × 10^3^**	7.36 × 10^3^	1.11 × 10^4^	1.44 × 10^4^	1.01 × 10^4^	7.00 × 10^3^	1.20 × 10^4^	7.15 × 10^3^
	Std	**2.79 × 10^2^**	8.47 × 10^2^	1.32 × 10^3^	9.62 × 10^2^	1.99 × 10^3^	3.24 × 10^2^	9.02 × 10^2^	8.96 × 10^2^
	Best	3.31 × 10^3^	3.52 × 10^3^	3.68 × 10^3^	3.67 × 10^3^	3.56 × 10^3^	3.58 × 10^3^	4.89 × 10^3^	**3.20 × 10^3^**
F27	Avg	3.53 × 10^3^	3.70 × 10^3^	3.98 × 10^3^	4.17 × 10^3^	3.99 × 10^3^	3.68 × 10^3^	5.96 × 10^3^	**3.20 × 10^3^**
	Std	1.81 × 10^2^	1.68 × 10^2^	2.84 × 10^2^	2.96 × 10^2^	2.87 × 10^2^	7.46 × 10^1^	7.65 × 10^1^	**1.57 × 10^0^**
	Best	**3.26 × 10^3^**	3.37 × 10^3^	3.41 × 10^3^	9.30 × 10^3^	3.40 × 10^3^	4.16 × 10^3^	5.12 × 10^3^	3.30 × 10^3^
F28	Avg	**3.20 × 10^3^**	3.70 × 10^3^	3.98 × 10^3^	4.17 × 10^3^	3.99 × 10^3^	3.68 × 10^3^	5.96 × 10^3^	3.30 × 10^3^
	Std	**3.78 × 10^1^**	1.97 × 10^1^	7.62 × 10^1^	1.32 × 10^3^	1.40 × 10^3^	4.65 × 10^2^	3.80 × 10^2^	2.97 × 10^4^
	Best	**3.95 × 10^3^**	4.49 × 10^3^	4.76 × 10^3^	6.14 × 10^3^	4.97 × 10^3^	4.34 × 10^3^	6.20 × 10^3^	4.63 × 10^3^
F29	Avg	**4.27 × 10^3^**	5.09 × 10^3^	5.37 × 10^3^	8.12 × 10^3^	6.15 × 10^3^	4.75 × 10^3^	7.84 × 10^3^	5.51 × 10^3^
	Std	**2.66 × 10^2^**	3.23 × 10^2^	3.50 × 10^2^	1.68 × 10^3^	6.14 × 10^2^	2.74 × 10^2^	1.06 × 10^3^	9.86 × 10^2^
	Best	**1.71 × 10^4^**	9.68 × 10^5^	1.51 × 10^6^	7.11 × 10^7^	7.94 × 10^6^	6.22 × 10^7^	2.24 × 10^8^	1.76 × 10^4^
F30	Avg	**1.50 × 10^6^**	179 × 10^6^	4.47 × 10^6^	1.96 × 10^9^	2.66 × 10^7^	1.43 × 10^8^	3.54 × 10^8^	2.47 × 10^6^
	Std	**5.12 × 10^5^**	6.71 × 10^5^	5.83 × 10^6^	2.06 × 10^9^	1.59 × 10^7^	6.12 × 10^7^	7.70 × 10^7^	3.95 × 10^6^

**Table 4:** Wilcoxon *p*-values on 30D CEC2017 functions.

F	WMA	IVY	PSO	DBO	GWO	GTO	LSHAED
F1	4.28 × 10^−1^	**7.21 × 10^−3^**	**1.73 × 10^−6^**	**1.73 × 10^−6^**	**1.73 × 10^−6^**	**1.73 × 10^−6^**	**1.73 × 10^−6^**
F3	**6.05 × 10^−3^**	**1.73 × 10^−6^**	**1.73 × 10^−6^**	**1.73 × 10^−6^**	**1.73 × 10^−6^**	**1.73 × 10^−6^**	**1.73 × 10^−6^**
F4	7.50 × 10^−1^	**1.19 × 10^−3^**	**1.73 × 10^−6^**	**2.17 × 10^−6^**	**1.73 × 10^−6^**	**1.73 × 10^−6^**	**3.07 × 10^−4^**
F5	5.44 × 10^−1^	**1.73 × 10^−6^**	**1.73 × 10^−6^**	**1.73 × 10^−6^**	**5.31 × 10^−5^**	**1.73 × 10^−6^**	**4.73 × 10^−6^**
F6	**1.73 × 10^−6^**	**3.51 × 10^−6^**	**1.73 × 10^−6^**	**1.73 × 10^−6^**	**1.73 × 10^−6^**	**1.73 × 10^−6^**	**3.88 × 10^−4^**
F7	**1.64 × 10^−5^**	**1.73 × 10^−6^**	**1.73 × 10^−6^**	**2.12 × 10^−6^**	**3.41 × 10^−5^**	**1.73 × 10^−6^**	**2.59 × 10^−3^**
F8	**6.84 × 10^−3^**	**2.13 × 10^−6^**	**1.73 × 10^−6^**	**1.73 × 10^−6^**	**3.11 × 10^−5^**	**1.73 × 10^−6^**	**2.60 × 10^−6^**
F9	**2.16 × 10^−5^**	**1.73 × 10^−6^**	**1.73 × 10^−6^**	**1.73 × 10^−6^**	**6.89 × 10^−5^**	**1.73 × 10^−6^**	**1.48 × 10^−2^**
F10	**3.61 × 10^−3^**	**7.12 × 10^−4^**	**2.70 × 10^−2^**	**2.85 × 10^−2^**	**8.94 × 10^−4^**	1.65 × 10^−1^	**7.16 × 10^−4^**
F11	**4.54 × 10^−4^**	**2.60 × 10^−6^**	**1.73 × 10^−6^**	**1.92 × 10^−6^**	**1.73 × 10^−6^**	**1.73 × 10^−6^**	**2.07 × 10^−2^**
F12	**7.16 × 10^−4^**	**1.92 × 10^−6^**	**1.73 × 10^−6^**	**1.92 × 10^−6^**	**1.73 × 10^−6^**	**1.73 × 10^−6^**	**1.92 × 10^−6^**
F13	9.26 × 10^−1^	**1.29 × 10^−3^**	**1.73 × 10^−6^**	**2.35 × 10^−6^**	**2.35 × 10^−6^**	**1.73 × 10^−6^**	**5.79 × 10^−5^**
F14	**4.86 × 10^−5^**	**1.73 × 10^−6^**	**1.92 × 10^−6^**	**1.73 × 10^−6^**	**1.73 × 10^−6^**	**1.73 × 10^−6^**	**2.70 × 10^−2^**
F15	**1.66 × 10^−2^**	7.87 × 10^−2^	**1.73 × 10^−6^**	**4.23 × 10^−6^**	**1.73 × 10^−6^**	**1.73 × 10^−6^**	**1.79 × 10^−5^**
F16	2.37 × 10^−1^	**3.82 × 10^−6^**	**1.92 × 10^−6^**	**2.13 × 10^−6^**	**2.30 × 10^−2^**	**1.73 × 10^−6^**	**6.98 × 10^−6^**
F17	**1.97 × 10^−3^**	**1.73 × 10^−6^**	**1.73 × 10^−6^**	**1.73 × 10^−6^**	**1.04 × 10^−2^**	**1.92 × 10^−6^**	**5.75 × 10^−6^**
F18	**1.18 × 10^−2^**	**3.88 × 10^−6^**	**1.73 × 10^−6^**	**1.73 × 10^−6^**	**1.13 × 10^−5^**	**1.92 × 10^−6^**	**7.69 × 10^−6^**
F19	**2.42 × 10^−3^**	**1.59 × 10^−3^**	**1.73 × 10^−6^**	**1.73 × 10^−6^**	**2.35 × 10^−6^**	**1.73 × 10^−6^**	**3.88 × 10^−4^**
F20	3.39 × 10^−1^	**1.73 × 10^−6^**	**1.92 × 10^−6^**	**4.73 × 10^−6^**	**1.59 × 10^−3^**	**2.13 × 10^−6^**	**4.87 × 10^−5^**
F21	**7.27 × 10^−3^**	**2.05 × 10^−4^**	**1.73 × 10^−6^**	**1.73 × 10^−6^**	**1.38 × 10^−3^**	**1.73 × 10^−6^**	**2.88 × 10^−6^**
F22	**6.64 × 10^−4^**	**2.23 × 10^−4^**	**1.73 × 10^−6^**	**1.73 × 10^−6^**	**1.73 × 10^−6^**	**1.73 × 10^−6^**	**1.73 × 10^−6^**
F23	**1.48 × 10^−4^**	**2.63 × 10^−6^**	**1.73 × 10^−6^**	**1.73 × 10^−6^**	**5.31 × 10^−5^**	**1.73 × 10^−6^**	**3.18 × 10^−6^**
F24	**8.22 × 10^−3^**	**6.89 × 10^−5^**	**1.73 × 10^−6^**	**1.73 × 10^−6^**	**1.15 × 10^−4^**	**1.73 × 10^−6^**	**2.88 × 10^−6^**
F25	9.75 × 10^−1^	**8.75 × 10^−4^**	**1.73 × 10^−6^**	**3.41 × 10^−5^**	**1.73 × 10^−6^**	**1.73 × 10^−6^**	**7.87 × 10^−6^**
F26	**2.59 × 10^−5^**	**1.92 × 10^−6^**	**1.92 × 10^−6^**	**1.73 × 10^−6^**	**8.18 × 10^−5^**	**1.92 × 10^−6^**	**4.39 × 10^−3^**
F27	**7.71 × 10^−4^**	**1.73 × 10^−6^**	**1.73 × 10^−6^**	**2.53 × 10^−6^**	**8.47 × 10^−6^**	**1.73 × 10^−6^**	**1.73 × 10^−6^**
F28	5.44 × 10^−1^	**1.73 × 10^−6^**	**1.73 × 10^−6^**	**1.73 × 10^−6^**	**1.73 × 10^−6^**	**1.73 × 10^−6^**	**1.73 × 10^−6^**
F29	**3.18 × 10^−6^**	**1.92 × 10^−6^**	**1.92 × 10^−6^**	**1.73 × 10^−6^**	**9.51 × 10^−6^**	**1.73 × 10^−6^**	**8.18 × 10^−5^**
F30	**7.97 × 10^−1^**	**1.92 × 10^−6^**	**1.73 × 10^−6^**	**1.73 × 10^−6^**	**1.73 × 10^−6^**	**1.73 × 10^−6^**	**1.75 × 10^−2^**

**Table 5:** Wilcoxon *p*-values on 50D CEC2017 functions.

F	WMA	IVY	PSO	DBO	GWO	GTO	LSHAED
F1	**1.73 × 10^−6^**	**2.56 × 10^−2^**	**1.73 × 10^−6^**	**1.73 × 10^−6^**	**1.73 × 10^−6^**	**1.73 × 10^−6^**	**4.86 × 10^−5^**
F3	**3.51 × 10^−6^**	**1.73 × 10^−6^**	**1.73 × 10^−6^**	**1.73 × 10^−6^**	**5.22 × 10^−6^**	**1.73 × 10^−6^**	**1.73 × 10^−6^**
F4	**8.73 × 10^−3^**	**5.67 × 10^−3^**	**1.73 × 10^−6^**	**3.18 × 10^−6^**	**1.73 × 10^−6^**	**1.73 × 10^−6^**	6.56 × 10^−2^
F5	5.86 × 10^−1^	**1.73 × 10^−6^**	**1.73 × 10^−6^**	**1.73 × 10^−6^**	**3.00 × 10^−2^**	**1.73 × 10^−6^**	**3.52 × 10^−6^**
F6	**1.73 × 10^−6^**	**2.35 × 10^−6^**	**1.73 × 10^−6^**	**1.73 × 10^−6^**	**2.35 × 10^−6^**	**1.73 × 10^−6^**	1.16 × 10^−1^
F7	**4.73 × 10^−6^**	**1.73 × 10^−6^**	**1.73 × 10^−6^**	**1.73 × 10^−6^**	**6.32 × 10^−5^**	**1.73 × 10^−6^**	**8.22 × 10^−3^**
F8	**1.57 × 10^−2^**	**1.73 × 10^−6^**	**1.73 × 10^−6^**	**1.73 × 10^−6^**	**4.89 × 10^−4^**	**1.73 × 10^−6^**	**4.29 × 10^−6^**
F9	**8.92 × 10^−5^**	**1.73 × 10^−6^**	**1.73 × 10^−6^**	**1.73 × 10^−6^**	3.34 × 10^−1^	**1.73 × 10^−6^**	6.87 × 10^−2^
F10	**1.73 × 10^−6^**	**1.73 × 10^−6^**	**1.73 × 10^−6^**	**1.73 × 10^−6^**	**1.73 × 10^−6^**	**1.73 × 10^−6^**	**1.73 × 10^−6^**
F11	7.97 × 10^−1^	**1.73 × 10^−6^**	**1.73 × 10^−6^**	**1.73 × 10^−6^**	**1.73 × 10^−6^**	**1.73 × 10^−6^**	**1.73 × 10^−6^**
F12	**1.13 × 10^−5^**	**6.03 × 10^−3^**	**1.73 × 10^−6^**	**1.73 × 10^−6^**	**1.73 × 10^−6^**	**1.73 × 10^−6^**	**1.73 × 10^−6^**
F13	5.19 × 10^−2^	**9.31 × 10^−6^**	**1.73 × 10^−6^**	**1.73 × 10^−6^**	**1.73 × 10^−6^**	**1.73 × 10^−6^**	**2.37 × 10^−6^**
F14	**3.88 × 10^−6^**	**1.73 × 10^−6^**	**1.73 × 10^−6^**	**2.13 × 10^−6^**	**1.93 × 10^−6^**	**1.73 × 10^−6^**	**2.63 × 10^−6^**
F15	9.10 × 10^−1^	**1.73 × 10^−6^**	**1.73 × 10^−6^**	**1.92 × 10^−6^**	**1.73 × 10^−6^**	**1.73 × 10^−6^**	**1.73 × 10^−6^**
F16	**1.60 × 10^−4^**	**7.69 × 10^−6^**	**1.73 × 10^−6^**	**1.73 × 10^−6^**	**1.85 × 10^−2^**	**1.73 × 10^−6^**	**2.60 × 10^−6^**
F17	**1.23 × 10^−6^**	**2.63 × 10^−6^**	**1.73 × 10^−6^**	**1.92 × 10^−6^**	**2.43 × 10^−2^**	**1.73 × 10^−6^**	**4.28 × 10^−6^**
F18	7.49 × 10^−1^	**1.73 × 10^−6^**	**1.73 × 10^−6^**	**1.73 × 10^−6^**	**1.73 × 10^−6^**	**1.73 × 10^−6^**	**1.73 × 10^−6^**
F19	5.43 × 10^−1^	**2.16 × 10^−5^**	**1.73 × 10^−6^**	**2.13 × 10^−6^**	**1.73 × 10^−6^**	**1.73 × 10^−6^**	**4.13 × 10^−3^**
F20	**1.39 × 10^−2^**	**1.49 × 10^−5^**	**3.13 × 10^−5^**	**8.43 × 10^−6^**	4.73 × 10^−1^	**9.31 × 10^−6^**	**2.87 × 10^−6^**
F21	**1.60 × 10^−4^**	**7.53 × 10^−5^**	**1.73 × 10^−6^**	**1.73 × 10^−6^**	**6.39 × 10^−6^**	**1.73 × 10^−6^**	**2.60 × 10^−6^**
F22	3.63 × 10^−1^	**1.13 × 10^−3^**	**1.17 × 10^−2^**	7.83 × 10^−2^	**6.13 × 10^−4^**	1.35 × 10^−1^	**2.07 × 10^−2^**
F23	**3.83 × 10^−6^**	**1.73 × 10^−5^**	**1.73 × 10^−6^**	**1.73 × 10^−6^**	**1.73 × 10^−6^**	**1.46 × 10^−5^**	**1.73 × 10^−6^**
F24	**1.48 × 10^−3^**	**5.73 × 10^−5^**	**1.73 × 10^−6^**	**1.73 × 10^−6^**	**7.73 × 10^−6^**	**1.73 × 10^−6^**	**3.88 × 10^−6^**
F25	**4.49 × 10^−5^**	**2.43 × 10^−3^**	**1.73 × 10^−6^**	**1.92 × 10^−6^**	**1.73 × 10^−6^**	**1.73 × 10^−6^**	**9.27 × 10^−3^**
F26	**4.94 × 10^−2^**	**1.73 × 10^−6^**	**1.73 × 10^−6^**	**1.05 × 10^−4^**	2.36 × 10^−1^	**2.35 × 10^−6^**	1.31 × 10^−1^
F27	**1.73 × 10^−6^**	**3.58 × 10^−4^**	**1.73 × 10^−6^**	**1.73 × 10^−6^**	**1.73 × 10^−6^**	**1.73 × 10^−6^**	**1.73 × 10^−6^**
F28	**7.69 × 10^−6^**	**3.58 × 10^−4^**	**1.73 × 10^−6^**	**1.73 × 10^−6^**	**1.73 × 10^−6^**	**1.73 × 10^−6^**	**2.87 × 10^−6^**
F29	**2.63 × 10^−6^**	**1.93 × 10^−6^**	**1.73 × 10^−6^**	**1.73 × 10^−6^**	**1.47 × 10^−4^**	**1.73 × 10^−6^**	**3.51 × 10^−6^**
F30	**2.83 × 10^−5^**	**2.87 × 10^−6^**	**1.73 × 10^−6^**	**1.73 × 10^−6^**	**1.73 × 10^−6^**	**1.73 × 10^−6^**	8.22 × 10^−2^

**Table 6:** Running time of algorithms on 30D CEC2017.

F	IWMA	WMA	IVY	PSO	DBO	GWO	GTO	LSHADE
F1	**3.24 × 10^−1^**	3.71 × 10^−1^	8.41 × 10^−1^	**4.23 × 10^−1^**	3.50 × 10^−1^	3.58 × 10^−1^	6.15 × 10^−1^	5.69 × 10^−1^
F3	**3.29 × 10^−1^**	3.91 × 10^−1^	7.48 × 10^−1^	5.23 × 10^−1^	3.82 × 10^−1^	3.33 × 10^−1^	6.23 × 10^−1^	5.55 × 10^−1^
F4	**3.78 × 10^−1^**	4.42 × 10^−1^	8.59 × 10^−1^	4.79 × 10^−1^	382 × 10^−1^	3.85 × 10^−1^	6.81 × 10^−1^	6.17 × 10^−1^
F5	**4.22 × 10^−1^**	4.81 × 10^−1^	9.11 × 10^−1^	5.37 × 10^−1^	4.39 × 10^−1^	4.36 × 10^−1^	8.48 × 10^−1^	6.51 × 10^−1^
F6	**5.14 × 10^−1^**	5.18 × 10^−1^	0.89 × 10^−1^	5.91 × 10^−1^	5.24 × 10^−1^	5.43 × 10^−1^	1.17 × 10^0^	6.33 × 10^−1^
F7	**4.01 × 10^−1^**	4.23 × 10^−1^	8.07 × 10^−1^	5.08 × 10^−1^	4.26 × 10^−1^	4.24 × 10^−1^	7.77 × 10^−1^	6.01 × 10^−1^
F8	**3.77 × 10^−1^**	4.16 × 10^−1^	7.80 × 10^−1^	4.97 × 10^−1^	3.90 × 10^−1^	3.86 × 10^−1^	9.67 × 10^−1^	5.78 × 10^−1^
F9	**3.81 × 10^−1^**	4.03 × 10^−1^	7.57 × 10^−1^	4.93 × 10^−1^	3.90 × 10^−1^	3.97 × 10^−1^	7.89 × 10^−1^	5.76 × 10^−1^
F10	4.16 × 10^−1^	4.39 × 10^−1^	7.53 × 10^−1^	4.56 × 10^−1^	3.73 × 10^−1^	**3.72 × 10^−1^**	6.85 × 10^−1^	5.41 × 10^−1^
F11	**3.64 × 10^−1^**	4.27 × 10^−1^	7.53 × 10^−1^	4.56 × 10^−1^	3.73 × 10^−1^	3.72 × 10^−1^	6.85 × 10^−1^	5.08 × 10^−1^
F12	**3.78 × 10^−1^**	4.12 × 10^−1^	7.66 × 10^−1^	5.23 × 10^−1^	3.87 × 10^−1^	3.80 × 10^−1^	7.52 × 10^−1^	5.47 × 10^−1^
F13	4.15 × 10^−1^	**3.88 × 10^−1^**	7.67 × 10^−1^	4.82 × 10^−1^	4.24 × 10^−1^	2.33 × 10^−1^	7.19 × 10^−1^	5.66 × 10^−1^
F14	4.23 × 10^−1^	**3.92 × 10^−1^**	8.27 × 10^−1^	6.90 × 10^−1^	4.66 × 10^−1^	4.50 × 10^−1^	9.11 × 10^−1^	5.98 × 10^−1^
F15	3.68 × 10^−1^	**3.24 × 10^−1^**	7.84 × 10^−1^	5.63 × 10^−1^	5.12 × 10^−1^	3.35 × 10^−1^	7.43 × 10^−1^	6.03 × 10^−1^
F16	**4.27 × 10^−1^**	4.36 × 10^−1^	9.12 × 10^−1^	5.66 × 10^−1^	4.42 × 10^−1^	4.33 × 10^−1^	8.16 × 10^−1^	6.60 × 10^−1^
F17	5.01 × 10^−1^	**4.98 × 10^−1^**	8.57 × 10^−1^	6.02 × 10^−1^	5.10 × 10^−1^	5.14 × 10^−1^	1.12 × 10^0^	6.93 × 10^−1^
F18	**4.03 × 10^−1^**	4.29 × 10^−1^	8.15 × 10^−1^	5.32 × 10^−1^	4.01 × 10^−1^	4.12 × 10^−1^	8.72 × 10^−1^	5.68 × 10^−1^
F19	9.90 × 10^−1^	**7.20 × 10^−1^**	1.48 × 10^0^	1.23 × 10^0^	1.18 × 10^0^	1.14 × 10^0^	3.09 × 10^0^	1.02 × 10^0^
F20	5.72 × 10^−1^	**4.20 × 10^−1^**	1.24 × 10^0^	7.76 × 10^−1^	6.20 × 10^−1^	6.79 × 10^−1^	1.26 × 10^0^	6.13 × 10^−1^
F21	5.70 × 10^−1^	**4.70 × 10^−1^**	1.04 × 10^−1^	9.59 × 10^−1^	8.84 × 10^−1^	5.90 × 10^−1^	1.71 × 10^0^	6.36 × 10^−1^
F22	6.21 × 10^−1^	**4.99 × 10^−1^**	1.01 × 10^0^	7.22 × 10^−1^	9.76 × 10^−1^	8.18 × 10^−1^	1.57 × 10^0^	7.23 × 10^−1^
F23	7.62 × 10^−1^	**6.68 × 10^−1^**	1.02 × 10^0^	8.07 × 10^−1^	8.55 × 10^−1^	7.59 × 10^−1^	2.02 × 10^0^	9.16 × 10^−1^
F24	8.27 × 10^−1^	**7.33 × 10^−1^**	1.27 × 10^0^	7.81 × 10^−1^	1.02 × 10^0^	8.83 × 10^−1^	2.16 × 10^0^	8.64 × 10^−1^
F25	7.29 × 10^−1^	**6.87 × 10^−1^**	1.06 × 10^0^	6.62 × 10^−1^	8.40 × 10^−1^	7.48 × 10^−1^	1.80 × 10^0^	7.58 × 10^−1^
F26	8.42 × 10^−1^	**7.02 × 10^−1^**	1.12 × 10^0^	8.17 × 10^−1^	8.90 × 10^−1^	8.78 × 10^−1^	2.19 × 10^0^	7.76 × 10^−1^
F27	9.16 × 10^−1^	8.73 × 10^−1^	1.22 × 10^0^	9.44 × 10^−1^	1.11 × 10^0^	1.02 × 10^0^	2.49 × 10^0^	**8.52 × 10^−1^**
F28	8.05 × 10^−1^	**7.20 × 10^−1^**	1.14 × 10^0^	7.48 × 10^−1^	9.10 × 10^−1^	8.70 × 10^−1^	2.03 × 10^0^	8.03 × 10^−1^
F29	6.96 × 10^−1^	**6.58 × 10^−1^**	1.06 × 10^0^	6.64 × 10^−1^	7.50 × 10^−1^	7.20 × 10^−1^	1.75 × 10^0^	7.81 × 10^−1^
F30	1.42 × 10^0^	1.22 × 10^0^	1.58 × 10^0^	1.27 × 10^0^	1.45 × 10^0^	1.55 × 10^0^	3.80 × 10^0^	**1.16 × 10^0^**

**Table 7:** Running time of algorithms on 50D CEC2017.

F	IWMA	WMA	IVY	PSO	DBO	GWO	GTO	LSHADE
F1	**4.46 × 10^−1^**	4.61 × 10^−1^	7.92 × 10^−1^	6.03 × 10^−1^	4.61 × 10^−1^	4.60 × 10^−1^	9.93 × 10^−1^	5.73 × 10^−1^
F3	4.73 × 10^−1^	**4.65 × 10^−1^**	8.93 × 10^−1^	8.39 × 10^−1^	4.94 × 10^−1^	4.84 × 10^−1^	1.04 × 10^0^	6.30 × 10^−1^
F4	**4.75 × 10^−1^**	4.80 × 10^−1^	8.79 × 10^−1^	8.19 × 10^−1^	4.98 × 10^−1^	5.22 × 10^−1^	1.1 × 10^0^	6.64 × 10^−1^
F5	5.68 × 10^−1^	5.67 × 10^−1^	9.17 × 10^−1^	6.98 × 10^−1^	**5.22 × 10^−1^**	5.83 × 10^−1^	1.32 × 10^0^	6.98 × 10^−1^
F6	7.75 × 10^−1^	**7.20 × 10^−1^**	1.12 × 10^0^	8.60 × 10^−1^	8.83 × 10^−1^	7.23 × 10^−1^	1.98 × 10^0^	7.69 × 10^−1^
F7	5.32 × 10^−1^	**5.29 × 10^−1^**	8.88 × 10^−1^	6.64 × 10^−1^	5.99 × 10^−1^	5.66 × 10^−1^	1.28 × 10^0^	6.26 × 10^−1^
F8	5.25 × 10^−1^	**5.12 × 10^−1^**	8.79 × 10^−1^	6.66 × 10^−1^	5.34 × 10^−1^	5.40 × 10^−1^	1.28 × 10^0^	6.24 × 10^−1^
F9	5.18 × 10^−1^	**4.83 × 10^−1^**	8.82 × 10^−1^	6.71 × 10^−1^	5.25 × 10^−1^	5.39 × 10^−1^	1.26 × 10^0^	6.24 × 10^−1^
F10	5.69 × 10^−1^	**5.51 × 10^−1^**	9.21 × 10^−1^	7.18 × 10^−1^	6.51 × 10^−1^	5.74 × 10^−1^	1.41 × 10^0^	6.50 × 10^−1^
F11	4.76 × 10^−1^	**4.23 × 10^−1^**	8.42 × 10^−1^	6.40 × 10^−1^	4.91 × 10^−1^	5.05 × 10^−1^	1.12 × 10^0^	6.01 × 10^−1^
F12	**5.18 × 10^−1^**	5.19 × 10^−1^	9.11 × 10^−1^	6.80 × 10^−1^	5.55 × 10^−1^	5.45 × 10^−1^	1.26 × 10^0^	6.31 × 10^−1^
F13	**6.62 × 10^−1^**	7.01 × 10^−1^	1.16 × 10^0^	7.92 × 10^−1^	6.93 × 10^−1^	6.96 × 10^−1^	1.49 × 10^0^	8.07 × 10^−1^
F14	7.17 × 10^−1^	7.21 × 10^−1^	**1.18 × 10^−1^**	7.63 × 10^−1^	7.60 × 10^−1^	7.11 × 10^−1^	1.71 × 10^0^	8.35 × 10^−1^
F15	**5.49 × 10^−1^**	5.55 × 10^−1^	9.76 × 10^−1^	6.60 × 10^−1^	5.53 × 10^−1^	5.90 × 10^−1^	1.24 × 10^0^	6.78 × 10^−1^
F16	**5.09 × 10^−1^**	5.11 × 10^−1^	8.95 × 10^−1^	6.56 × 10^−1^	5.92 × 10^−1^	5.29 × 10^−1^	1.21 × 10^0^	6.18 × 10^−1^
F17	**7.08 × 10^−1^**	7.09 × 10^−1^	1.04 × 10^0^	8.40 × 10^−1^	7.49 × 10^−1^	7.87 × 10^−1^	1.84 × 10^0^	7.86 × 10^−1^
F18	5.02 × 10^−1^	**4.91 × 10^−1^**	8.68 × 10^−1^	6.58 × 10^−1^	5.91 × 10^−1^	5.22 × 10^−1^	1.19 × 10^0^	6.13 × 10^−1^
F19	1.70 × 10^0^	1.23 × 10^0^	1.84 × 10^0^	1.64 × 10^0^	1.85 × 10^0^	1.49 × 10^0^	5.05 × 10^0^	**1.20 × 10^0^**
F20	7.78 × 10^−1^	7.98 × 10^−1^	1.12 × 10^0^	1.09 × 10^0^	7.95 × 10^−1^	**7.20 × 10^−1^**	2.04 × 10^0^	8.26 × 10^−1^
F21	1.01 × 10^0^	**9.81 × 10^−1^**	1.38 × 10^0^	1.4 × 10^0^	1.08 × 10^0^	1.05 × 10^0^	2.78 × 10^0^	1.05 × 10^0^
F22	1.11 × 10^0^	**8.83 × 10^−1^**	1.40 × 10^0^	1.01 × 10^0^	1.15 × 10^0^	1.15 × 10^0^	3.06 × 10^0^	1.11 × 10^0^
F23	1.30 × 10^0^	**7.82 × 10^−1^**	1.54 × 10^0^	1.59 × 10^0^	1.22 × 10^0^	1.12 × 10^0^	3.57 × 10^0^	9.51 × 10^−1^
F24	1.35 × 10^0^	1.12 × 10^0^	1.58 × 10^0^	1.63 × 10^0^	1.15 × 10^0^	1.28 × 10^0^	3.84 × 10^0^	**1.08 × 10** **^0^**
F25	1.31 × 10^0^	**1.02 × 10** **^0^**	1.55 × 10^0^	1.74 × 10^0^	1.23 × 10^0^	1.16 × 10^0^	3.65 × 10^0^	1.24 × 10^0^
F26	1.51 × 10^0^	1.13 × 10^0^	1.72 × 10^0^	**1.07 × 10** **^0^**	1.27 × 10^0^	1.57 × 10^0^	4.55 × 10^0^	1.53 × 10^0^
F27	1.81 × 10^0^	**1.23 × 10** **^0^**	2.02 × 10^0^	2.35 × 10^0^	1.42 × 10^0^	1.24 × 10^0^	5.20 × 10^0^	1.32 × 10^0^
F28	1.63 × 10^0^	**1.28 × 10** **^0^**	1.90 × 10^0^	2.07 × 10^0^	1.28 × 10^0^	1.34 × 10^0^	4.66 × 10^0^	1.47 × 10^0^
F29	1.22 × 10^0^	**1.09 × 10** **^0^**	1.47 × 10^0^	1.60 × 10^0^	1.12 × 10^0^	1.27 × 10^0^	3.35 × 10^0^	1.33 × 10^0^
F30	2.20 × 10^0^	1.85 × 10^0^	2.31 × 10^0^	2.60 × 10^0^	1.71 × 10^0^	1.88 × 10^0^	6.61 × 10^0^	**1.52 × 10** **^0^**

**Table 8:** Versions of various IWMA.

Algorithm	Key Component I	Key Component II	Key Component III
WMA	X	X	X
IWMA1	O	X	X
IWMA2	X	O	X
IWMA3	X	X	O
IWMA12	O	O	X
IWMA13	O	X	O
IWMA23	X	O	O
IWMA	O	O	O

**Table 9:** Ablation study results on CEC2017 benchmarks (30D).

F		IWMA	WMA	IWMA1	IWMA2	IWMA3	IWMA12	IWMA13	IWMA23
	Best	**1.64 × 10^2^**	1.68 × 10^2^	1.65 × 10^2^	8.17 × 10^6^	4.48 × 10^2^	2.44 × 10^10^	2.79 × 10^2^	1.66 × 10^2^
F1	Avg	**4.43 × 10^3^**	6.64 × 10^3^	5.68 × 10^3^	1.33 × 10^10^	4.69 × 10^3^	2.58 × 10^10^	8.76 × 10^3^	5.18 × 10^3^
	Std	**5.19 × 10^3^**	6.89 × 10^3^	6.36 × 10^3^	2.64 × 10^9^	5.45 × 10^3^	5.57 × 10^8^	7.18 × 10^3^	5.39 × 10^3^
	Best	5.01 × 10^3^	**2.28 × 10^3^**	4.86 × 10^3^	4.04 × 10^3^	5.74 × 10^3^	5.01 × 10^4^	5.73 × 10^3^	4.64 × 10^3^
F3	Avg	**1.20 × 10^4^**	1.66 × 10^4^	2.01 × 10^4^	7.49 × 10^4^	2.01 × 10^4^	5.17 × 10^4^	3.02 × 10^4^	1.21 × 10^4^
	Std	4.19 × 10^3^	8.88 × 10^3^	1.15 × 10^4^	1.98 × 10^4^	9.11 × 10^3^	**1.01 × 10^3^**	1.71 × 10^4^	4.23 × 10^3^
	Best	**4.06 × 10^2^**	**4.58 × 10^2^**	4.64 × 10^2^	1.65 × 10^3^	4.59 × 10^2^	4.61 × 10^3^	4.14 × 10^2^	4.26 × 10^2^
F4	Avg	**4.81 × 10^2^**	4.84 × 10^2^	4.87 × 10^2^	3.81 × 10^3^	4.88 × 10^2^	5.19 × 10^2^	4.82 × 10^2^	5.06 × 10^2^
	Std	2.72 × 10^1^	**1.36 × 10^1^**	1.57 × 10^1^	1.15 × 10^3^	2.07 × 10^1^	5.36 × 10^2^	3.18 × 10^1^	2.88 × 10^1^
	Best	5.55 × 10^2^	5.55 × 10^2^	5.66 × 10^2^	6.82 × 10^2^	**5.43 × 10^2^**	8.11 × 10^2^	5.81 × 10^2^	5.57 × 10^2^
F5	Avg	**5.89 × 10^2^**	6.26 × 10^2^	6.11 × 10^2^	7.39 × 10^2^	6.17 × 10^2^	8.21 × 10^2^	6.55 × 10^2^	5.91 × 10^2^
	Std	1.83 × 10^1^	5.35 × 10^1^	3.51 × 10^1^	3.09 × 10^1^	5.49 × 10^1^	3.47 × 10^0^	**6.46 × 10^1^**	2.14 × 10^1^
	Best	**6.00 × 10^2^**	6.12 × 10^2^	6.05 × 10^2^	6.49 × 10^2^	6.07 × 10^2^	6.65 × 10^2^	6.02 × 10^2^	6.01 × 10^2^
F6	Avg	**6.02 × 10^2^**	6.23 × 10^2^	6.21 × 10^2^	6.61 × 10^2^	6.19 × 10^2^	6.67 × 10^2^	6.43 × 10^2^	6.02 × 10^2^
	Std	**1.44 × 10^2^**	6.89 × 10^0^	6.03 × 10^0^	5.78 × 10^0^	6.06 × 10^0^	1.95 × 10^0^	1.95 × 10^1^	1.81 × 10^0^
	Best	**7.76 × 10^2^**	7.87 × 10^2^	8.08 × 10^2^	1.05 × 10^3^	8.09 × 10^2^	1.32 × 10^3^	8.17 × 10^2^	7.76 × 10^2^
F7	Avg	**8.31 × 10^2^**	9.09 × 10^2^	9.14 × 10^2^	1.24 × 10^3^	8.98 × 10^2^	1.34 × 10^3^	9.34 × 10^2^	8.35 × 10^2^
	Std	4.07 × 10^1^	4.99 × 10^1^	6.05 × 10^1^	1.11 × 10^2^	5.41 × 10^1^	**1.03 × 10^1^**	1.02 × 10^2^	3.72 × 10^1^
	Best	**8.43 × 10^2^**	8.47 × 10^2^	8.47 × 10^2^	9.01 × 10^2^	8.49 × 10^2^	9.95 × 10^2^	8.50 × 10^2^	8.51 × 10^2^
F8	Avg	**8.69 × 10^2^**	9.01 × 10^2^	8.90 × 10^2^	9.58 × 10^2^	8.88 × 10^2^	1.02 × 10^3^	9.69 × 10^2^	8.69 × 10^2^
	Std	1.74 × 10^1^	4.39 × 10^1^	2.70 × 10^1^	2.84 × 10^1^	3.01 × 10^1^	**9.94 × 10^0^**	9.54 × 10^1^	1.39 × 10^1^
	Best	**9.21 × 10^2^**	1.05 × 10^3^	1.41 × 10^3^	3.54 × 10^3^	1.15 × 10^3^	5.57 × 10^3^	1.12 × 10^3^	9.23 × 10^2^
F9	Avg	**1.21 × 10^3^**	2.67 × 10^3^	3.15 × 10^3^	5.07 × 10^3^	3.05 × 10^3^	5.77 × 10^3^	6.14 × 10^3^	1.22 × 10^3^
	Std	**3.70 × 10^2^**	1.04 × 10^3^	1.46 × 10^3^	6.47 × 10^2^	1.57 × 10^3^	1.79 × 10^2^	4.15 × 10^3^	3.74 × 10^2^
	Best	**3.43 × 10^3^**	5.83 × 10^3^	6.44 × 10^3^	3.78 × 10^3^	6.53 × 10^3^	4.79 × 10^3^	3.75 × 10^3^	3.74 × 10^3^
F10	Avg	6.65 × 10^3^	7.19 × 10^3^	7.22 × 10^3^	5.69 × 10^3^	7.18 × 10^3^	**5.31 × 10^3^**	5.89 × 10^3^	6.29 × 10^3^
	Std	1.26 × 10^3^	4.21 × 10^2^	3.57 × 10^2^	7.83 × 10^2^	3.37 × 10^2^	**3.09 × 10^2^**	8.57 × 10^2^	1.58 × 10^3^
	Best	**1.14 × 10^3^**	1.16 × 10^3^	1.16 × 10^3^	1.41 × 10^3^	1.17 × 10^3^	2.28 × 10^3^	1.19 × 10^3^	1.11 × 10^3^
F11	Avg	**1.20 × 10^3^**	1.29 × 10^3^	1.30 × 10^3^	2.47 × 10^3^	1.28 × 10^3^	2.63 × 10^3^	1.36 × 10^3^	1.22 × 10^3^
	Std	**3.43 × 10^1^**	7.90 × 10^1^	7.59 × 10^1^	1.02 × 10^3^	7.06 × 10^1^	9.82 × 10^1^	9.22 × 10^1^	6.26 × 10^1^
	Best	4.97 × 10^4^	1.74 × 10^4^	**1.36 × 10^4^**	2.03 × 10^8^	2.14 × 10^4^	5.33 × 10^4^	1.26 × 10^4^	7.85 × 10^4^
F12	Avg	4.34 × 10^5^	2.65 × 10^5^	**2.53 × 10^5^**	2.18 × 10^9^	2.91 × 10^5^	5.82 × 10^9^	1.86 × 10^5^	5.92 × 10^5^
	Std	4.73 × 10^5^	4.59 × 10^5^	**3.06 × 10^5^**	1.21 × 10^9^	3.96 × 10^5^	5.26 × 10^8^	3.46 × 10^5^	4.22 × 10^5^
	Best	2.14 × 10^3^	2.59 × 10^3^	3.16 × 10^3^	2.53 × 10^4^	1.92 × 10^3^	8.64 × 10^8^	**1.52 × 10^3^**	2.47 × 10^3^
F13	Avg	2.07 × 10^4^	3.01 × 10^4^	1.18 × 10^4^	3.75 × 10^8^	2.68 × 10^4^	2.10 × 10^9^	**7.27 × 10^3^**	2.22 × 10^4^
	Std	**1.68 × 10^4^**	2.04 × 10^4^	6.78 × 10^3^	4.90 × 10^8^	2.38 × 10^4^	1.81 × 10^9^	6.75 × 10^3^	2.23 × 10^4^
	Best	**1.59 × 10^3^**	1.60 × 10^3^	2.04 × 10^3^	1.98 × 10^3^	1.73 × 10^3^	1.87 × 10^3^	2.17 × 10^3^	1.76 × 10^3^
F14	Avg	**3.99 × 10^3^**	1.64 × 10^4^	1.32 × 10^4^	4.77 × 10^5^	1.55 × 10^4^	2.33 × 10^6^	2.12 × 10^4^	5.56 × 10^3^
	Std	**3.26 × 10^3^**	1.04 × 10^4^	1.01 × 10^4^	8.43 × 10^5^	1.22 × 10^4^	2.95 × 10^5^	1.33 × 10^4^	4.61 × 10^3^
	Best	**1.62 × 10^3^**	2.02 × 10^3^	1.82 × 10^3^	5.60 × 10^3^	2.18 × 10^3^	1.43 × 10^4^	1.69 × 10^3^	1.71 × 10^3^
F15	Avg	**6.97 × 10^3^**	1.11 × 10^4^	1.31 × 10^4^	1.97 × 10^4^	1.21 × 10^4^	1.77 × 10^4^	8.85 × 10^3^	8.36 × 10^3^
	Std	**1.02 × 10^4^**	1.05 × 10^4^	1.25 × 10^4^	7.76 × 10^3^	1.05 × 10^4^	1.74 × 10^3^	1.15 × 10^4^	5.88 × 10^3^
	Best	**1.61 × 10^3^**	1.87 × 10^3^	2.14 × 10^3^	2.66 × 10^3^	1.87 × 10^3^	4.86 × 10^3^	1.63 × 10^3^	1.71 × 10^3^
F16	Avg	**2.29 × 10^3^**	2.62 × 10^3^	2.55 × 10^3^	3.71 × 10^3^	2.53 × 10^3^	5.00 × 10^3^	2.44 × 10^3^	2.37 × 10^3^
	Std	4.02 × 10^2^	3.41 × 10^2^	2.68 × 10^2^	5.81 × 10^2^	3.46 × 10^2^	**7.64 × 10^1^**	2.79 × 10^2^	3.22 × 10^2^
	Best	**1.74 × 10^3^**	1.86 × 10^3^	1.85 × 10^3^	2.14 × 10^3^	1.82 × 10^3^	3.08 × 10^3^	1.82 × 10^3^	1.75 × 10^3^
F17	Avg	**1.93 × 10^3^**	2.14 × 10^3^	2.29 × 10^3^	2.93 × 10^3^	2.18 × 10^3^	3.28 × 10^3^	2.19 × 10^3^	1.94 × 10^3^
	Std	**1.66 × 10^2^**	2.07 × 10^2^	2.39 × 10^2^	3.19 × 10^2^	2.10 × 10^2^	8.65 × 10^2^	2.15 × 10^2^	1.71 × 10^2^
	Best	**2.22 × 10^4^**	2.16 × 10^4^	**2.03 × 10^4^**	7.33 × 10^4^	2.34 × 10^4^	1.48 × 10^6^	5.66 × 10^4^	2.78 × 10^4^
F18	Avg	**1.44 × 10^5^**	2.45 × 10^5^	1.93 × 10^5^	1.29 × 10^6^	2.02 × 10^5^	2.24 × 10^6^	2.45 × 10^5^	2.17 × 10^5^
	Std	**1.18 × 10^5^**	2.18 × 10^5^	1.46 × 10^5^	1.24 × 10^6^	2.03 × 10^5^	4.41 × 10^5^	2.71 × 10^5^	2.11 × 10^5^
	Best	**2.04 × 10^3^**	2.16 × 10^3^	2.07 × 10^3^	2.05 × 10^4^	2.09 × 10^3^	8.77 × 10^5^	2.04 × 10^3^	2.05 × 10^3^
F19	Avg	**9.13 × 10^3^**	1.34 × 10^4^	9.15 × 10^3^	1.14 × 10^6^	1.14 × 10^4^	1.19 × 10^6^	8.45 × 10^3^	1.27 × 10^4^
	Std	**9.14 × 10^3^**	1.60 × 10^4^	8.47 × 10^3^	4.93 × 10^5^	1.43 × 10^4^	1.48 × 10^5^	5.11 × 10^3^	1.08 × 10^4^
	Best	**2.04 × 10^3^**	2.13 × 10^3^	2.09 × 10^3^	2.29 × 10^3^	2.11 × 10^3^	3.18 × 10^3^	2.07 × 10^3^	2.11 × 10^3^
F20	Avg	**2.23 × 10^3^**	2.35 × 10^3^	2.42 × 10^3^	2.87 × 10^3^	2.39 × 10^3^	3.29 × 10^3^	2.54 × 10^3^	2.37 × 10^3^
	Std	1.98 × 10^2^	1.22 × 10^2^	1.80 × 10^2^	2.49 × 10^2^	1.58 × 10^2^	**1.02 × 10^2^**	2.16 × 10^2^	182 × 10^2^
	Best	**2.33 × 10^3^**	2.35 × 10^3^	2.35 × 10^3^	2.49 × 10^3^	2.35 × 10^3^	2.66 × 10^3^	2.21 × 10^3^	2.34 × 10^3^
F21	Avg	**2.36 × 10^3^**	2.39 × 10^3^	2.38 × 10^3^	2.58 × 10^3^	2.40 × 10^3^	2.70 × 10^3^	2.42 × 10^3^	2.38 × 10^3^
	Std	**1.90 × 10^1^**	3.66 × 10^1^	4.23 × 10^1^	5.38 × 10^1^	3.29 × 10^1^	4.45 × 10^1^	8.06 × 10^1^	2.58 × 10^1^
	Best	**2.30 × 10^3^**	2.30 × 10^3^	2.30 × 10^3^	5.94 × 10^3^	2.30 × 10^3^	7.92 × 10^3^	2.30 × 10^3^	2.30 × 10^3^
F22	Avg	**2.31 × 10^3^**	4.97 × 10^3^	5.37 × 10^3^	7.4 × 10^3^	4.72 × 10^3^	8.27 × 10^3^	4.91 × 10^3^	2.69 × 10^3^
	Std	**6.09 × 10^1^**	2.83 × 10^3^	3.12 × 10^3^	6.76 × 10^2^	3.02 × 10^3^	1.40 × 10^2^	2.16 × 10^3^	1.42 × 10^3^
	Best	**2.69 × 10^3^**	2.71 × 10^3^	2.70 × 10^3^	3.13 × 10^3^	2.71 × 10^3^	4.27 × 10^3^	2.73 × 10^3^	2.70 × 10^3^
F23	Avg	**2.73 × 10^3^**	2.77 × 10^3^	2.77 × 10^3^	3.58 × 10^3^	2.77 × 10^3^	4.32 × 10^3^	2.82 × 10^3^	2.74 × 10^3^
	Std	**2.61 × 10^1^**	3.56 × 10^1^	3.77 × 10^1^	2.18 × 10^2^	3.18 × 10^1^	2.23 × 10^1^	6.87 × 10^1^	2.64 × 10^1^
	Best	**2.85 × 10^3^**	2.86 × 10^3^	2.87 × 10^3^	3.46 × 10^3^	2.88 × 10^3^	4.37 × 10^3^	2.88 × 10^3^	2.86 × 10^3^
F24	Avg	**2.88 × 10^3^**	2.92 × 10^3^	2.94 × 10^3^	3.73 × 10^3^	2.93 × 10^3^	4.37 × 10^3^	2.97 × 10^3^	2.90 × 10^3^
	Std	2.14 × 10^1^	3.23 × 10^1^	4.87 × 10^1^	1.30 × 10^2^	3.78 × 10^1^	**1.47 × 10^0^**	5.77 × 10^1^	2.03 × 10^1^
	Best	**2.88 × 10^3^**	2.88 × 10^3^	2.89 × 10^3^	3.09 × 10^3^	2.86 × 10^3^	3.29 × 10^3^	2.89 × 10^3^	2.89 × 10^3^
F25	Avg	**2.89 × 10^3^**	2.90 × 10^3^	2.90 × 10^3^	3.27 × 10^3^	2.90 × 10^3^	3.34 × 10^3^	2.89 × 10^3^	2.89 × 10^3^
	Std	1.64 × 10^1^	1.71 × 10^1^	1.53 × 10^1^	9.47 × 10^1^	1.69 × 10^1^	6.38 × 10^1^	**6.87 × 10^0^**	1.62 × 10^1^
	Best	**2.80 × 10^3^**	2.80 × 10^3^	4.36 × 10^3^	7.98 × 10^3^	4.35 × 10^3^	9.86 × 10^3^	2.80 × 10^3^	2.81 × 10^3^
F26	Avg	**4.19 × 10^3^**	5.10 × 10^3^	5.18 × 10^3^	9.12 × 10^3^	5.11 × 10^3^	1.04 × 10^3^	4.55 × 10^3^	4.58 × 10^3^
	Std	7.64 × 10^2^	6.49 × 10^2^	5.20 × 10^2^	5.84 × 10^2^	4.96 × 10^2^	2.43 × 10^2^	**1.46 × 10^2^**	7.52 × 10^2^
	Best	**3.19 × 10^3^**	3.21 × 10^3^	3.23 × 10^3^	3.98 × 10^3^	3.23 × 10^3^	6.30 × 10^3^	3.21 × 10^3^	3.20 × 10^3^
F27	Avg	**3.24 × 10^3^**	3.26 × 10^3^	3.28 × 10^3^	4.59 × 10^3^	3.27 × 10^3^	6.74 × 10^3^	3.26 × 10^3^	3.25 × 10^3^
	Std	**2.14 × 10^1^**	2.78 × 10^1^	3.95 × 10^1^	3.38 × 10^2^	2.81 × 10^1^	2.09 × 10^2^	3.09 × 10^1^	2.23 × 10^1^
	Best	**3.09 × 10^3^**	3.10 × 10^3^	3.10 × 10^3^	3.93 × 10^3^	3.10 × 10^3^	4.92 × 10^3^	3.10 × 10^3^	3.19 × 10^3^
F28	Avg	**3.21 × 10^3^**	3.22 × 10^3^	3.23 × 10^3^	4.49 × 10^3^	3.23 × 10^3^	5.02 × 10^3^	3.23 × 10^3^	3.22 × 10^3^
	Std	**2.09 × 10^1^**	4.23 × 10^1^	3.66 × 10^1^	2.82 × 10^2^	4.45 × 10^1^	5.47 × 10^1^	6.24 × 10^1^	2.14 × 10^1^
	Best	**3.41 × 10^3^**	3.61 × 10^3^	3.39 × 10^3^	4.41 × 10^3^	3.61 × 10^3^	5.89 × 10^3^	3.72 × 10^3^	3.39 × 10^3^
F29	Avg	**3.65 × 10^3^**	4.12 × 10^3^	4.03 × 10^3^	5.57 × 10^3^	3.99 × 10^3^	6.14 × 10^3^	4.10 × 10^3^	3.65 × 10^3^
	Std	**1.42 × 10^2^**	2.51 × 10^2^	2.90 × 10^2^	5.09 × 10^2^	2.12 × 10^2^	1.49 × 10^2^	2.71 × 10^2^	1.44 × 10^2^
	Best	**4.19 × 10^3^**	6.16 × 10^3^	5.33 × 10^3^	3.72 × 10^5^	5.32 × 10^3^	4.11 × 10^8^	7.05 × 10^3^	6.75 × 10^3^
F30	Avg	**1.26 × 10^4^**	1.44 × 10^4^	8.70 × 10^4^	2.49 × 10^7^	1.28 × 10^4^	5.22 × 10^8^	1.27 × 10^4^	1.39 × 10^4^
	Std	**4.13 × 10^3^**	8.33 × 10^3^	4.08 × 10^5^	3.58 × 10^7^	5.72 × 10^3^	5.20 × 10^7^	4.21 × 10^3^	5.18 × 10^3^

**Table 10:** Ablation study results on CEC2017 benchmarks (50D).

F		IWMA	WMA	IWMA1	IWMA2	IWMA3	IWMA12	IWMA13	IWMA23
	Best	1.97 × 10^5^	**1.94 × 10^2^**	3.59 × 10^2^	3.74 × 10^10^	2.38 × 10^2^	5.99 × 10^10^	2.67 × 10^4^	2.51 × 10^5^
F1	Avg	2.07 × 10^6^	**2.80 × 10^4^**	9.61 × 10^3^	1.81 × 10^5^	1.29 × 10^5^	1.92 × 10^5^	2.35 × 10^5^	2.35 × 10^5^
	Std	3.27 × 10^6^	4.23 × 10^4^	**9.50 × 10^3^**	8.16 × 10^9^	3.17 × 10^4^	6.97 × 10^8^	5.14 × 10^8^	3.34 × 10^6^
	Best	**6.01 × 10^4^**	8.98 × 10^4^	8.67 × 10^4^	1.31 × 10^5^	8.55 × 10^4^	1.45 × 10^5^	1.34 × 10^4^	6.02 × 10^4^
F3	Avg	**7.87 × 10^4^**	1.48 × 10^5^	1.36 × 10^5^	1.81 × 10^5^	1.29 × 10^5^	1.92 × 10^5^	2.35 × 10^5^	7.93 × 10^4^
	Std	**1.17 × 10^4^**	3.80 × 10^4^	3.06 × 10^4^	3.84 × 10^4^	2.72 × 10^4^	2.59 × 10^4^	7.34 × 10^4^	1.30 × 10^4^
	Best	4.83 × 10^2^	4.85 × 10^2^	5.07 × 10^2^	5.40 × 10^3^	**4.16 × 10^2^**	1.55 × 10^4^	5.16 × 10^2^	4.78 × 10^2^
F4	Avg	5.79 × 10^2^	5.81 × 10^2^	5.66 × 10^2^	9.87 × 10^3^	**5.42 × 10^2^**	1.61 × 10^4^	6.35 × 10^2^	5.93 × 10^2^
	Std	5.35 × 10^1^	4.65 × 10^1^	**3.57 × 10^1^**	2.21 × 10^3^	5.60 × 10^1^	2.56 × 10^2^	8.56 × 10^1^	5.16 × 10^1^
	Best	**6.03 × 10^2^**	6.40 × 10^2^	6.24 × 10^2^	6.58 × 10^2^	6.23 × 10^2^	6.71 × 10^2^	6.40 × 10^2^	6.40 × 10^2^
F5	Avg	**7.07 × 10^2^**	7.39 × 10^2^	7.44 × 10^2^	8.48 × 10^2^	7.33 × 10^2^	8.92 × 10^2^	8.05 × 10^2^	7.11 × 10^2^
	Std	4.28 × 10^1^	6.13 × 10^1^	4.59 × 10^1^	4.18 × 10^1^	6.77 × 10^1^	**2.15 × 10^1^**	5.17 × 10^1^	4.04 × 10^1^
	Best	**6.03 × 10^2^**	6.25 × 10^2^	6.24 × 10^2^	6.58 × 10^2^	6.23 × 10^2^	6.71 × 10^2^	6.40 × 10^2^	6.07 × 10^2^
F6	Avg	**6.12 × 10^2^**	6.38 × 10^2^	6.37 × 10^2^	6.67 × 10^2^	6.33 × 10^2^	6.74 × 10^2^	6.48 × 10^2^	6.14 × 10^2^
	Std	4.99 × 10^0^	9.63 × 10^0^	9.25 × 10^0^	4.98 × 10^0^	5.14 × 10^0^	**4.17 × 10^0^**	6.57 × 10^0^	5.65 × 10^0^
	Best	**8.95 × 10^2^**	1.02 × 10^3^	1.02 × 10^3^	1.49 × 10^3^	1.04 × 10^3^	1.78 × 10^3^	1.32 × 10^3^	9.28 × 10^2^
F7	Avg	**1.01 × 10^3^**	1.23 × 10^3^	1.18 × 10^3^	1.78 × 10^3^	1.22 × 10^3^	1.79 × 10^3^	1.54 × 10^3^	1.02 × 10^3^
	Std	6.43 × 10^1^	1.32 × 10^2^	1.06 × 10^2^	1.44 × 10^2^	1.20 × 10^2^	**6.06 × 10^0^**	1.43 × 10^2^	7.55 × 10^1^
	Best	**9.47 × 10^2^**	9.71 × 10^2^	9.66 × 10^2^	1.10 × 10^3^	9.57 × 10^2^	1.22 × 10^3^	9.83 × 10^2^	9.50 × 10^2^
F8	Avg	**1.00 × 10^3^**	1.04 × 10^3^	1.04 × 10^3^	1.16 × 10^3^	1.07 × 10^3^	1.23 × 10^3^	1.10 × 10^3^	1.00 × 10^3^
	Std	3.41 × 10^1^	4.09 × 10^1^	5.66 × 10^1^	3.75 × 10^1^	9.33 × 10^1^	**1.66 × 10^0^**	6.12 × 10^1^	3.80 × 10^1^
	Best	**2.03 × 10^3^**	7.54 × 10^3^	7.57 × 10^3^	1.26 × 10^4^	6.88 × 10^3^	1.55 × 10^4^	1.51 × 10^4^	2.53 × 10^3^
F9	Avg	**6.16 × 10^3^**	1.49 × 10^4^	1.50 × 10^4^	1.48 × 10^4^	1.39 × 10^4^	1.81 × 10^4^	2.87 × 10^4^	7.07 × 10^3^
	Std	**1.22 × 10^3^**	5.24 × 10^3^	5.22 × 10^3^	1.51 × 10^3^	4.73 × 10^3^	1.86 × 10^3^	7.42 × 10^3^	2.83 × 10^3^
	Best	7.43 × 10^3^	1.24 × 10^4^	1.14 × 10^4^	6.90 × 10^3^	1.19 × 10^4^	7.98 × 10^3^	**6.17 × 10^3^**	1.19 × 10^4^
F10	Avg	1.30 × 10^4^	1.32 × 10^4^	1.30 × 10^4^	8.82 × 10^3^	1.32 × 10^4^	8.54 × 10^3^	1.33 × 10^4^	**1.24 × 10^4^**
	Std	**1.73 × 10^3^**	4.40 × 10^2^	5.98 × 10^2^	6.58 × 10^2^	4.93 × 10^2^	4.62 × 10^2^	3.70 × 10^3^	5.20 × 10^2^
	Best	**1.25 × 10^3^**	1.26 × 10^3^	1.29 × 10^3^	5.43 × 10^3^	1.29 × 10^3^	9.20 × 10^3^	1.34 × 10^3^	1.30 × 10^3^
F11	Avg	**1.37 × 10^3^**	1.41 × 10^3^	1.39 × 10^3^	1.20 × 10^4^	1.41 × 10^3^	1.32 × 10^4^	1.48 × 10^3^	1.38 × 10^3^
	Std	**4.95 × 10^1^**	7.08 × 10^1^	5.79 × 10^1^	3.31 × 10^3^	7.85 × 10^1^	3.92 × 10^3^	8.05 × 10^1^	5.07 × 10^1^
	Best	**4.09 × 10^5^**	6.67 × 10^5^	4.20 × 10^5^	1.33 × 10^10^	4.19 × 10^5^	4.98 × 10^10^	7.64 × 10^5^	1.46 × 10^6^
F12	Avg	7.06 × 10^6^	**2.75 × 10^6^**	3.06 × 10^6^	2.36 × 10^10^	4.04 × 10^6^	5.11 × 10^10^	1.15 × 10^8^	5.86 × 10^6^
	Std	**1.01 × 10^6^**	2.02 × 10^6^	1.92 × 10^6^	7.37 × 10^9^	3.42 × 10^6^	9.19 × 10^8^	4.34 × 10^8^	6.19 × 10^6^
	Best	**3.67 × 10^3^**	5.76 × 10^3^	4.54 × 10^3^	5.47 × 10^8^	4.92 × 10^3^	2.81 × 10^10^	6.10 × 10^3^	4.17 × 10^3^
F13	Avg	**1.00 × 10^4^**	1.67 × 10^4^	2.40 × 10^4^	6.61 × 10^9^	1.55 × 10^4^	2.92 × 10^10^	6.41 × 10^7^	1.01 × 10^4^
	Std	**6.34 × 10^3^**	1.00 × 10^4^	1.84 × 10^4^	4.14 × 10^9^	1.21 × 10^4^	7.50 × 10^8^	2.87 × 10^8^	9.27 × 10^3^
	Best	1.24 × 10^4^	**5.36 × 10^3^**	2.31 × 10^4^	1.14 × 10^5^	3.37 × 10^3^	6.63 × 10^7^	9.08 × 10^3^	1.60 × 10^4^
F14	Avg	6.86 × 10^4^	9.84 × 10^4^	8.17 × 10^4^	5.71 × 10^6^	**5.84 × 10^4^**	7.22 × 10^7^	1.77 × 10^5^	6.07 × 10^4^
	Std	**6.29 × 10^4^**	1.17 × 10^5^	6.39 × 10^4^	4.73 × 10^6^	6.88 × 10^4^	4.17 × 10^6^	2.15 × 10^5^	7.02 × 10^4^
	Best	**2.04 × 10^3^**	2.05 × 10^3^	2.06 × 10^3^	3.16 × 10^3^	2.28 × 10^3^	6.94 × 10^3^	2.77 × 10^3^	2.08 × 10^3^
F15	Avg	1.26 × 10^4^	1.04 × 10^4^	1.07 × 10^4^	4.46 × 10^7^	1.34 × 10^4^	8.98 × 10^8^	1.24 × 10^4^	**8.67 × 10^3^**
	Std	**6.50 × 10^3^**	6.59 × 10^3^	8.19 × 10^3^	1.04 × 10^8^	9.64 × 10^3^	7.89 × 10^7^	8.17 × 10^3^	8.28 × 10^3^
	Best	**2.25 × 10^3^**	2.81 × 10^3^	2.76 × 10^3^	3.32 × 10^3^	2.49 × 10^3^	7.59 × 10^3^	2.56 × 10^3^	2.26 × 10^3^
F16	Avg	**2.84 × 10^3^**	3.23 × 10^3^	3.45 × 10^3^	4.86 × 10^3^	3.10 × 10^3^	7.81 × 10^3^	3.54 × 10^3^	2.88 × 10^3^
	Std	**3.46 × 10^2^**	3.51 × 10^2^	4.79 × 10^2^	9.55 × 10^2^	2.55 × 10^2^	8.94 × 10^1^	5.90 × 10^2^	3.60 × 10^2^
	Best	**2.13 × 10^3^**	2.64 × 10^3^	2.42 × 10^3^	3.30 × 10^3^	2.42 × 10^3^	3.83 × 10^3^	2.94 × 10^3^	2.30 × 10^3^
F17	Avg	**2.81 × 10^3^**	3.18 × 10^3^	3.10 × 10^3^	4.17 × 10^3^	3.21 × 10^3^	4.20 × 10^3^	3.50 × 10^3^	2.83 × 10^3^
	Std	**2.38 × 10^2^**	3.58 × 10^2^	3.90 × 10^2^	4.37 × 10^2^	3.68 × 10^2^	3.78 × 10^2^	2.86 × 10^2^	3.12 × 10^2^
	Best	1.09 × 10^5^	9.14 × 10^4^	**7.29 × 10^4^**	5.37 × 10^5^	7.91 × 10^4^	3.69 × 10^7^	1.73 × 10^5^	1.08 × 10^5^
F18	Avg	7.21 × 10^5^	3.63 × 10^5^	**3.36 × 10^5^**	1.33 × 10^7^	3.49 × 10^5^	5.18 × 10^7^	4.89 × 10^5^	7.48 × 10^5^
	Std	6.12 × 10^5^	7.74 × 10^5^	**3.04 × 10^5^**	7.98 × 10^6^	1.77 × 10^5^	6.03 × 10^6^	3.32 × 10^5^	6.72 × 10^5^
	Best	3.24 × 10^3^	**2.10 × 10^3^**	2.89 × 10^3^	6.13 × 10^4^	2.31 × 10^3^	9.54 × 10^7^	2.26 × 10^3^	6.24 × 10^3^
F19	Avg	1.87 × 10^4^	**1.12 × 10^4^**	1.50 × 10^4^	9.17 × 10^5^	1.86 × 10^4^	2.20 × 10^8^	1.78 × 10^4^	2.25 × 10^4^
	Std	**1.00 × 10^4^**	1.13 × 10^4^	1.24 × 10^4^	7.44 × 10^5^	1.95 × 10^4^	1.47 × 10^8^	1.38 × 10^4^	1.23 × 10^4^
	Best	**2.23 × 10^3^**	2.45 × 10^3^	2.83 × 10^3^	2.66 × 10^3^	2.24 × 10^3^	3.63 × 10^3^	2.59 × 10^3^	2.29 × 10^3^
F20	Avg	**2.96 × 10^3^**	3.30 × 10^3^	3.29 × 10^3^	3.53 × 10^3^	3.14 × 10^3^	3.85 × 10^3^	3.09 × 10^3^	2.97 × 10^3^
	Std	3.95 × 10^2^	3.48 × 10^2^	2.41 × 10^2^	4.66 × 10^2^	4.15 × 10^2^	**1.94 × 10^2^**	4.58 × 10^2^	4.64 × 10^2^
	Best	**2.41 × 10^3^**	2.50 × 10^3^	2.74 × 10^3^	2.73 × 10^3^	2.46 × 10^3^	2.93 × 10^3^	2.51 × 10^3^	2.42 × 10^3^
F21	Avg	**2.49 × 10^3^**	2.54 × 10^3^	2.54 × 10^3^	2.91 × 10^3^	2.53 × 10^3^	3.00 × 10^3^	2.62 × 10^3^	2.50 × 10^3^
	Std	**3.92 × 10^1^**	5.05 × 10^1^	5.71 × 10^1^	1.04 × 10^2^	4.34 × 10^1^	4.98 × 10^1^	7.27 × 10^1^	6.61 × 10^1^
	Best	**2.60 × 10^3^**	1.27 × 10^4^	7.07 × 10^3^	9.60 × 10^3^	1.34 × 10^4^	1.06. × 10^4^	8.80 × 10^3^	2.61 × 10^3^
F22	Avg	1.19 × 10^4^	1.44 × 10^4^	1.40 × 10^4^	**1.12 × 10^4^**	1.46 × 10^4^	1.24 × 10^4^	1.47 × 10^4^	1.32 × 10^4^
	Std	3.71 × 10^3^	5.55 × 10^2^	1.74 × 10^3^	1.06 × 10^3^	**5.10 × 10^2^**	1.05 × 10^3^	2.91 × 10^3^	4.19 × 10^3^
	Best	**2.84 × 10^3^**	2.94 × 10^3^	2.89 × 10^3^	4.03 × 10^3^	2.97 × 10^3^	4.56 × 10^3^	3.16 × 10^3^	2.87 × 10^3^
F23	Avg	**2.93 × 10^3^**	3.06 × 10^3^	3.05 × 10^3^	4.44 × 10^3^	3.04 × 10^3^	4.67 × 10^3^	3.39 × 10^3^	2.94 × 10^3^
	Std	**5.48 × 10^1^**	7.31 × 10^1^	7.15 × 10^1^	2.49 × 10^2^	5.66 × 10^1^	5.54 × 10^1^	1.60 × 10^2^	5.58 × 10^1^
	Best	**3.02 × 10^3^**	3.03 × 10^3^	3.07 × 10^3^	4.31 × 10^3^	3.08 × 10^3^	6.21 × 10^3^	3.46 × 10^3^	3.03 × 10^3^
F24	Avg	**3.11 × 10^3^**	3.16 × 10^3^	3.17 × 10^3^	4.79 × 10^3^	3.20 × 10^3^	6.24 × 10^3^	3.71 × 10^3^	3.14 × 10^3^
	Std	**1.57 × 10^1^**	7.11 × 10^1^	7.46 × 10^1^	2.71 × 10^2^	8.02 × 10^1^	1.78 × 10^1^	1.43 × 10^2^	5.20 × 10^1^
	Best	**2.95 × 10^3^**	3.06 × 10^3^	3.05 × 10^3^	6.02 × 10^3^	2.96 × 10^3^	8.16 × 10^3^	3.02 × 10^3^	3.06 × 10^3^
F25	Avg	3.11 × 10^3^	3.05 × 10^3^	**3.04 × 10^3^**	7.37 × 10^3^	3.06 × 10^3^	8.27 × 10^3^	3.13 × 10^3^	3.12 × 10^3^
	Std	**3.23 × 10^1^**	3.44 × 10^1^	3.33 × 10^1^	8.56 × 10^2^	4.11 × 10^1^	8.12 × 10^1^	6.68 × 10^1^	3.49 × 10^1^
	Best	**2.91 × 10^3^**	5.70 × 10^3^	5.82 × 10^3^	1.19 × 10^4^	5.70 × 10^3^	1.35 × 10^4^	8.69 × 10^3^	2.93 × 10^3^
F26	Avg	**5.54 × 10^3^**	7.49 × 10^3^	6.97 × 10^3^	1.39 × 10^4^	7.31 × 10^3^	1.41 × 10^4^	1.17 × 10^4^	5.83 × 10^3^
	Std	2.46 × 10^3^	7.75 × 10^2^	5.97 × 10^2^	7.84 × 10^2^	7.39 × 10^2^	**2.92 × 10^2^**	1.65 × 10^3^	2.86 × 10^3^
	Best	**3.30 × 10^3^**	3.35 × 10^3^	3.52 × 10^3^	5.75 × 10^3^	3.47 × 10^3^	9.82 × 10^3^	3.37 × 10^3^	3.31 × 10^3^
F27	Avg	**3.45 × 10^3^**	3.69 × 10^3^	3.70 × 10^3^	6.59 × 10^3^	3.67 × 10^3^	1.02 × 10^4^	3.87 × 10^3^	3.49 × 10^3^
	Std	**9.75 × 10^1^**	2.07 × 10^2^	1.00 × 10^2^	4.57 × 10^2^	1.74 × 10^2^	2.02 × 10^2^	3.47 × 10^2^	1.84 × 10^2^
	Best	**3.21 × 10^3^**	3.26 × 10^3^	3.25 × 10^3^	6.44 × 10^3^	3.26 × 10^3^	8.89 × 10^3^	3.32 × 10^3^	3.31 × 10^3^
F28	Avg	3.38 × 10^3^	3.31 × 10^3^	**3.31 × 10^3^**	7.51 × 10^3^	3.31 × 10^3^	9.04 × 10^3^	4.21 × 10	3.38 × 10^3^
	Std	**2.21 × 10^1^**	2.22 × 10^1^	1.36 × 10^1^	5.63 × 10^2^	2.99 × 10^1^	8.80 × 10^1^	1.23 × 10^3^	4.25 × 10^1^
	Best	**3.64 × 10^3^**	3.84 × 10^3^	4.54 × 10^3^	7.89 × 10^3^	4.13 × 10^3^	2.24 × 10^4^	4.57 × 10^3^	3.62 × 10^3^
F29	Avg	**4.31 × 10^3^**	5.14 × 10^3^	5.24 × 10^3^	1.15 × 10^4^	5.08 × 10^3^	2.61 × 10^4^	5.18 × 10^4^	4.40 × 10^3^
	Std	**3.76 × 10^2^**	5.81 × 10^2^	4.56 × 10^2^	2.55 × 10^3^	4.39 × 10^2^	1.85 × 10^3^	4.59 × 10^2^	4.23 × 10^2^
	Best	**9.03 × 10^5^**	7.85 × 10^5^	9.48 × 10^5^	1.93 × 10^8^	8.01 × 10^5^	1.89 × 10^9^	9.59 × 10^5^	9.05 × 10^5^
F30	Avg	**1.22 × 10^6^**	2.09 × 10^6^	2.18 × 10^6^	3.96 × 10^8^	1.93 × 10^6^	2.17 × 10^9^	2.25 × 10^6^	1.46 × 10^6^
	Std	**4.15 × 10^5^**	1.28 × 10^6^	1.12 × 10^6^	2.57 × 10^8^	9.59 × 10^5^	1.75 × 10^8^	1.22 × 10^6^	5.62 × 10^5^

**Table 11:** Single vs. Multi−UAV Threat Features.

Feature	Single UAV Path Threat ($B/f_{2}$)	Multi-UAV Waypoint Threat ($c/f_{3}$)
**Target**	Single UAV, path segment, single obstacle	Single UAV waypoint, multi-UAV formation, threat spheres
**Distance metric**	Minimum distance from path segment to obstacle center $d_{k}$	Distance from UAV waypoint to threat sphere center $d_{i,k}^{j}$
**Cost function**	Piecewise: collision=1, danger zone linearly decreasing, safe = 0	Region−dependent formula, nonlinearly decreasing with distance
**Accumulation**	Sum over all path segments single UAV total cost $f_{2}$	Sum over all waypoints and threat spheres → single UAV cost $f_{t,i}$, formation total cost $f_{3}$
**Application level**	Single UAV path planning	Multi−UAV formation path planning
**Characteristics**	Local granularity, 2D obstacle projection	Waypoint granularity, 3D threat spheres, suitable for formation−level evaluation

**Table 12:** Comparison of fitness values of different algorithms.

Scenario		IWMA	WMA	IVY	PSO	DBO	GWO	GTO	LSHADE
	best	**2250.84**	2301.98	2415.48	2680.04	2921.34	2405.43	2731.94	2621.68
Scenario 1	worst	**2290.91**	2427.45	2592.26	2889.16	3020.76	2426.29	2926.01	2703.94
	mean	**2277.09**	2353.19	2478.61	2810.79	2985.05	2418.44	2817.26	2659.18
	best	**2463.70**	2551.20	2865.51	3146.70	2584.94	2597.88	2589.57	2971.75
Scenario 2	worst	**2481.03**	2587.88	2895.74	3617.13	2805.38	2636.74	2630.78	3077.15
	mean	**2471.19**	2572.28	2881.12	3348.77	2722.55	2614.21	2611.26	3024.02
	best	**2721.93**	2801.08	3222.29	3677.49	3092.87	2975.55	3025.92	3334.54
Scenario 3	worst	**2811.81**	4334.88	3671.35	4119.74	4264.51	3414.71	3063.96	3398.87
	mean	**2756.66**	3552.29	3404.11	3852.97	3807.31	3193.43	3039.89	3353.54
	best	**610.21**	642.92	1480.96	1177.29	1431.11	892.70	1499.99	819.40
Scenario 4	worst	**650.19**	713.20	1497.79	1325.76	1461.36	1100.04	1519.94	1561.37
	mean	**627.86**	684.49	1489.66	1232.63	1450.47	997.61	1509.68	1068.28
	best	**620.25**	699.96	1456.84	938.79	630.22	819.19	1481.37	722.07
Scenario 5	worst	**640.33**	808.99	1502.16	1196.46	891.26	859.83	1495.39	1087.64
	mean	**629.75**	761.59	1484.79	1097.41	727.59	833.49	1487.87	894.21
	best	**713.33**	870.13	1547.59	1226.47	779.09	930.71	1808.08	1366.89
Scenario 6	worst	**785.65**	1564.47	1563.11	1502.69	1038.75	1060.23	1854.89	1678.08
	mean	**750.54**	1105.55	1553.09	1396.76	930.84	989.34	1831.13	1509.55

## Data Availability

Available upon request.
